# A Novel Approach for Continuous Health Status Monitoring and Automatic Detection of Infection Incidences in People With Type 1 Diabetes Using Machine Learning Algorithms (Part 2): A Personalized Digital Infectious Disease Detection Mechanism

**DOI:** 10.2196/18912

**Published:** 2020-08-12

**Authors:** Ashenafi Zebene Woldaregay, Ilkka Kalervo Launonen, David Albers, Jorge Igual, Eirik Årsand, Gunnar Hartvigsen

**Affiliations:** 1 Department of Computer Science University of Tromsø – The Arctic University of Norway Tromsø Norway; 2 Department of Clinical Research University Hospital of North Norway Tromsø Norway; 3 Department of Pediatrics, Informatics and Data Science University of Colorado Aurora, CO United States; 4 Department of Biomedical Informatics Columbia University New York, NY United States; 5 Universidad Politecnica Valencia Valencia Spain

**Keywords:** type 1 diabetes, self-recorded health data, infection detection, decision support techniques, outbreak detection system, syndromic surveillance

## Abstract

**Background:**

Semisupervised and unsupervised anomaly detection methods have been widely used in various applications to detect anomalous objects from a given data set. Specifically, these methods are popular in the medical domain because of their suitability for applications where there is a lack of a sufficient data set for the other classes. Infection incidence often brings prolonged hyperglycemia and frequent insulin injections in people with type 1 diabetes, which are significant anomalies. Despite these potentials, there have been very few studies that focused on detecting infection incidences in individuals with type 1 diabetes using a dedicated personalized health model.

**Objective:**

This study aims to develop a personalized health model that can automatically detect the incidence of infection in people with type 1 diabetes using blood glucose levels and insulin-to-carbohydrate ratio as input variables. The model is expected to detect deviations from the norm because of infection incidences considering elevated blood glucose levels coupled with unusual changes in the insulin-to-carbohydrate ratio.

**Methods:**

Three groups of one-class classifiers were trained on target data sets (regular days) and tested on a data set containing both the target and the nontarget (infection days). For comparison, two unsupervised models were also tested. The data set consists of high-precision self-recorded data collected from three real subjects with type 1 diabetes incorporating blood glucose, insulin, diet, and events of infection. The models were evaluated on two groups of data: raw and filtered data and compared based on their performance, computational time, and number of samples required.

**Results:**

The one-class classifiers achieved excellent performance. In comparison, the unsupervised models suffered from performance degradation mainly because of the atypical nature of the data. Among the one-class classifiers, the boundary and domain-based method produced a better description of the data. Regarding the computational time, nearest neighbor, support vector data description, and self-organizing map took considerable training time, which typically increased as the sample size increased, and only local outlier factor and connectivity-based outlier factor took considerable testing time.

**Conclusions:**

We demonstrated the applicability of one-class classifiers and unsupervised models for the detection of infection incidence in people with type 1 diabetes. In this patient group, detecting infection can provide an opportunity to devise tailored services and also to detect potential public health threats. The proposed approaches achieved excellent performance; in particular, the boundary and domain-based method performed better. Among the respective groups, particular models such as one-class support vector machine, K-nearest neighbor, and K-means achieved excellent performance in all the sample sizes and infection cases. Overall, we foresee that the results could encourage researchers to examine beyond the presented features into other additional features of the self-recorded data, for example, continuous glucose monitoring features and physical activity data, on a large scale.

## Introduction

Anomaly or novelty detection problem involves identifying the anomalous or novel instances, which exhibit different characteristics, from the rest of the data set and has been widely used in various applications including machine fault and sensor failure detection, prevention of credit card or identity fraud, health and medical diagnostics and monitoring, cyber-intrusion detection, and others [1-7]. The term anomaly was precisely coined by Hawkins [8] as “observations that deviate much from the other observations so as to arouse suspicions that it could be generated by a different process.” Anomalousness is usually described as point, contextual, and collective, depending on how the degree of anomaly is computed [1,7,9]. On the basis of the necessity of having labeled data instances for the respective class, the anomaly detection problem can be approached as supervised, semisupervised, and unsupervised [3,7,9-11]. Supervised anomaly detection, for example, multiclass classification, requires labeled data instances for both the target and the nontarget (anomaly) classes. This characteristic makes it impractical for tasks where there is difficulty in either finding enough samples for the anomaly class, that is, poorly sampled and unbalanced data, or demarcating boundaries of the anomaly class [7,10,12]. Moreover, anomalies could also evolve over time, and what is known today might not be valid through time, making the characterization of anomalies class more challenging. In this case, semisupervised anomaly detection, that is, one-class classification, is preferred given that it only requires characterizing what is believed to be normal (target data instances) to detect the abnormal (nontarget data instances) [7]. Under certain circumstances, for example, medical domain, obtaining and demarcating the anomalous (nontarget) data instances can become very difficult, expensive, and time consuming, if not impossible [7,13]. For instance, assume a health diagnostic and monitoring system that detects health changes in an individual by tracking the individual’s physiological parameters, where the current health status is examined based on a set of parameters, and raises a notification alarm when the individual health deteriorates [12]. In such a system, it becomes feasible to rely on a method that can be trained using only the regular or normal day measurements (target days) so as to detect deviation from normality [12,14]. This is because demarcating the exact boundaries between normal and abnormal health conditions is very challenging given that each pathogen has a different effect on the individual physiology. The one-class classifiers–based anomaly detection methods can be roughly grouped into 3 main groups: boundary and domain-based, density-based, and reconstruction-based methods based on how their internal function is defined and the approach used for minimization [3,10,12,13,15,16]. These models take into account different characteristics of the data set, and depending on the data set under consideration, these models could achieve different generalization performance, overfitting, and bias [12]. Unlike supervised and semisupervised anomaly detection methods, unsupervised methods do not require labeled instances to detect the anomaly (nontarget) instances because they rely on the entire data set to determine the anomalies and can be another possible alternative to semisupervised anomaly detection methods [7,10,12]*.* One of the drawbacks of unsupervised methods is that they require significant amount of data to achieve comparable performance. Both semisupervised and unsupervised methods have been used in various applications to detect anomalous instances [1,7,10,16]. In particular, these methods have been popular in the medical domain owing to their suitability for such applications, where there is lack of a sufficient data set for the other classes [13]. Accordingly, considering the difficulty and expense of obtaining enough sample data sets for the infection days from people with type 1 diabetes, a one-class classifier and unsupervised models are proposed for detecting infection incidence in people with type 1 diabetes.

Type 1 diabetes, also known as insulin-dependent diabetes, is a chronic disease of blood glucose regulation (hemostasis), and is caused by the lack of insulin secretion from pancreatic cells [17,18]. In people with type 1 diabetes, the incidence of infection often results in hyperglycemia and frequent insulin injection [19-26]. Infection-induced anomalies are characterized by violation of the norm of blood glucose dynamics, where blood glucose remains elevated despite taking a higher amount of insulin injection with less carbohydrate consumption [19]. Despite these potentials, there have been very few studies that focused on detecting infection incidence in individuals with type 1 diabetes using a dedicated personalized health model. Therefore, the objective of this study was to develop an algorithm, that is, a personalized health model that can automatically detect the incidence of infection in people with type 1 diabetes using blood glucose levels and insulin-to-carbohydrate ratio as input variables. For this, a one-class classifier and unsupervised models are proposed. The model is expected to detect any deviations from the norm because of infection incidences considering elevated blood glucose level (hyperglycemia incidences) coupled with unusual changes in the insulin-to-carbohydrate ratio, that is, frequent insulin injections and unusual reduction in the amount of carbohydrate intake [19]. Three groups of one-class classifiers and two unsupervised density-based models were explored. A detailed theoretical description of the proposed models is given in Multimedia Appendix 1 [1,7-16,27-37]. The anomaly detection problem studied in this paper can be regarded as a contextual anomaly, where the ratio of insulin-to-carbohydrate is the context and the average blood glucose level is the behavioral attribute. This is mainly because of the fact that elevated blood glucose levels do not always signify being anomalies without looking at the context of the ratio of insulin-to-carbohydrate in this case. Throughout the paper, the term object is used to describe a feature vector incorporating the number of parameters under consideration. For example, an object X can define a specific event of an individual blood glucose dynamics at a specified time index *k* and is represented by a feature vector X*_k_*=(x***_k,1_***, x***_k,2_***), where x***_k,1_*** represents the ratio of total insulin-to-total carbohydrate and x***_k,2_*** represents the average blood glucose level in a specific time-bin (interval) around *k*.

## Methods

**Figure 1:**
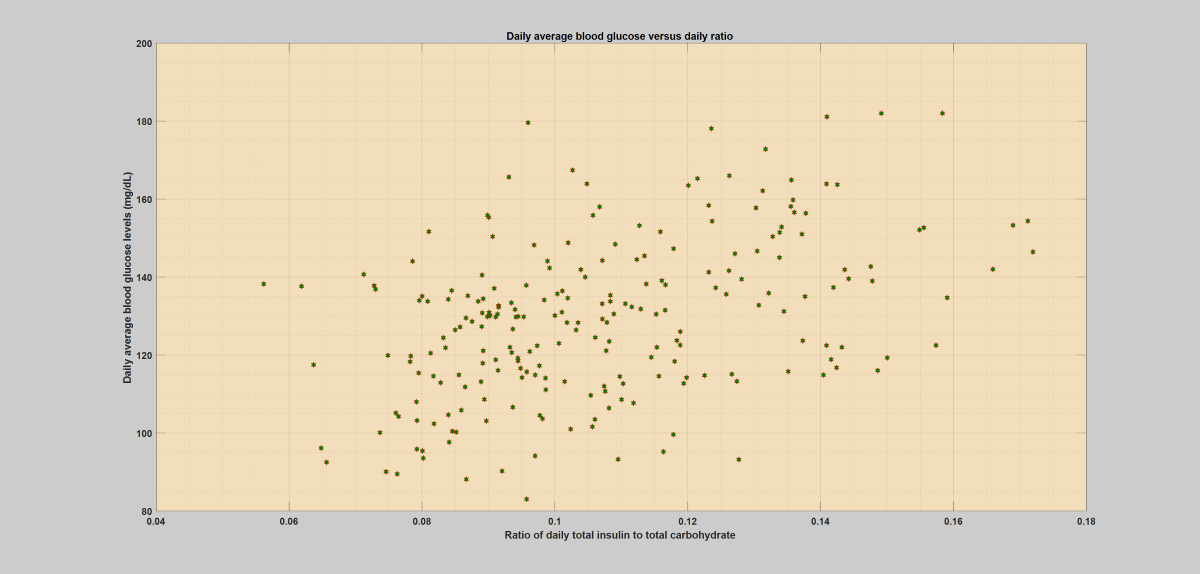
Daily scatter plot of average blood glucose levels versus total insulin (bolus) to total carbohydrate ratio for a specific regular or normal patient year without any infection incidences.

**Figure 2:**
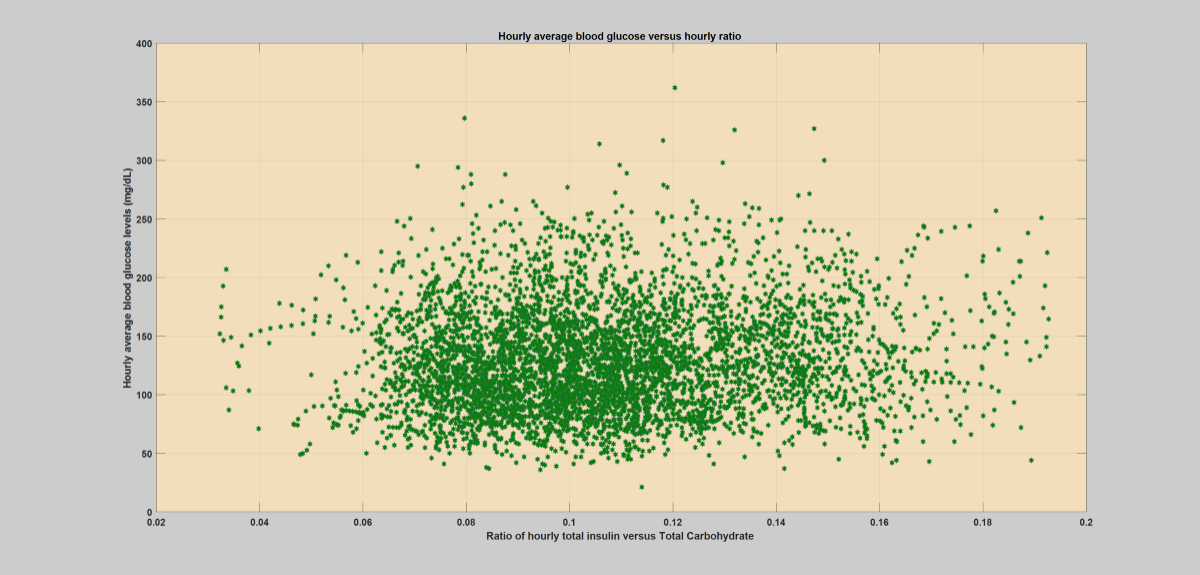
Hourly scatter plot of average blood glucose levels versus total insulin (bolus) to total carbohydrate ratio for a specific regular or normal patient year without any infection incidences.

**Figure 3:**
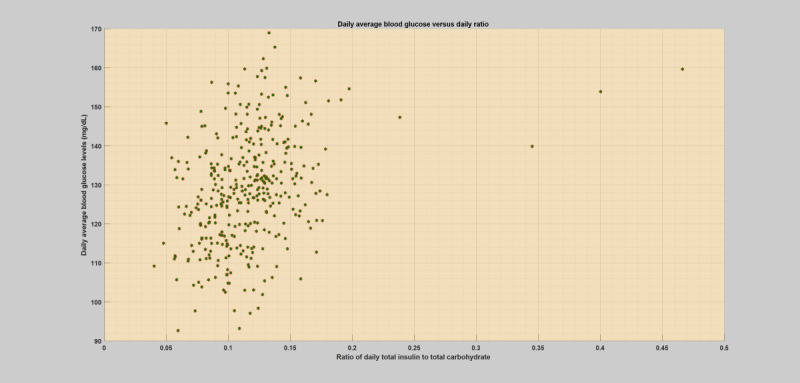
Daily scatter plot of average blood glucose levels versus total insulin (bolus) to total carbohydrate ratio for a specific patient year with an infection incidence (flu).

**Figure 4:**
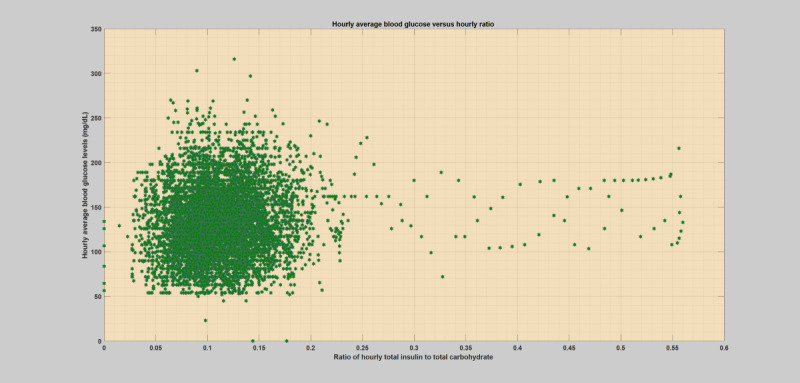
Hourly scatter plot of average blood glucose levels versus total insulin (bolus) to total carbohydrate ratio for a specific patient year with an infection incidence (flu).

A group of one-class classifiers and unsupervised models were tested and compared. The one-class classifier incorporates 3 groups: boundary and domain-based, density-based, and reconstruction-based methods. The boundary and domain-based method contains support vector data description (SVDD) [27], one-class support vector machine (V-SVM) [28], incremental support vector machine [29], nearest neighbor (NN) [12], and minimum spanning tree (MST) [15]. Density-based method includes normal Gaussian [32], minimum covariance Gaussian [38], mixture of Gaussian (MOG) [32], Parzen [39], naïve Parzen [32], K-nearest neighbor (KNN) [12,30], and local outlier factor (LOF) [31]. The reconstruction-based method includes principal component analysis (PCA) [12,32], K-means [32], self-organizing maps (SOM) [12,32], and auto-encoder networks [12]. In addition, the unsupervised models were also tested, including the LOF [31,33] and the connectivity-based outlier factor (COF) [33,34]. The input variables, average blood glucose levels and ratio of total insulin (bolus) to total carbohydrate, used in training and testing of the models were selected in accordance with the description provided by Woldaregay et al [19], and the ratio was calculated by dividing the total insulin with the total carbohydrate within a specified time-bin. The data set consists of high-precision self-recorded data collected from 3 real subjects (2 males and 1 female; average age 34 [SD 13.2] years) with type 1 diabetes. It incorporates blood glucose levels, insulin, carbohydrate information, and self-reported infections cases of influenza (flu) and, mild and light common cold without fever, as shown in Table 1. Exemplar data depicting the model’s input features for 2 specific patient years with and without infection are shown in Figures 1-4, and a more detailed description of the input features for 10-patient years with and without infection incidences can be found in Multimedia Appendix 2 [12,19]. The data were resampled and imputed in accordance with the description provided by Woldaregay et al [19], and the preprocessed data were smoothed using a moving average filter of 2 days’ (48 hours) window size to remove short-term and small-scale features [19,40,41]. Feature scaling was carried out using min-max scaling [42] to normalize the data between 0 and 1, which is important to ensure that larger parameters do not dominate the smaller ones. The data sets are labeled as target and nontarget data sets, where the target data sets include all the self-recorded normal period of the year and the nontarget data set includes only the self-reported infection periods when the individual was sick. Accordingly, the one-class classifiers were trained using only the target data sets containing the regular or normal period of the year and tested using both the target and the nontarget (infection period) data sets. For the unsupervised models, all the data sets containing both the target and the nontarget data sets were presented during testing. The hyperparameters of most of the one-class classifiers were optimized using a consistency approach [43]. Models such as naïve Parzen and Parzen were optimized using the leave-one-out method. For MST, the entire MST was used. For PCA, the fraction of variance retained from the training data set was set to be 0.67. The models were evaluated based on different characteristics including data nature (with and without filter), data granularity (hourly and daily), data sample size, and required computational time. All the experiments were conducted using MATLAB 2018b (Mathworks, Inc). Most of the models were implemented using *ddtools, prtools,* and *anomaly detection toolbox*, which are MATLAB toolboxes [32,33,35].

**Table 1:** Equipments used in the self-management of diabetes.

Patients	Self-management				
	BG^a^	Insulin administration	Diet	Body weight (kg)	HbA_1c_^b^ (%)
Subject 1	Finger pricks recorded in the Diabetes Diary mobile app and Dexcom CGM^c^	Insulin Pen (multiple bolus and 1-time basal in the morning) recorded in the Diabetes Diary mobile app	Carbohydrate in grams recorded in the Diabetes Diary mobile app; level 3 (advanced carb counting)	83	6.0
Subject 2	Finger pricks recorded in the Spike mobile app and Dexcom G4 CGM^c^	Insulin Pen (multiple bolus [Humalog] and 1-time basal [Toujeo] before bed) recorded in the Spike mobile app	Carbohydrate in grams recorded in the Spike mobile app; level 3 (advanced carb counting)	77	7.3
Subject 3	Enlite (Medtronic) CGM^c^ and Dexcom G4	Medtronic MinMed G640 insulin pump (basal rates profile [Fiasp] and multiple bolus [Fiasp])	Carbohydrate in grams recorded in pump information; level 3 (advanced carb counting)	70	6.2

^a^BG: blood glucose.

^b^HbA_1c_: hemoglobin A_1c_.

^c^CGM: continuous glucose monitoring.

### Model Evaluation

The performance of the one-class classifiers was evaluated using 20 times 5-fold stratified cross-validation. For both daily and hourly cases, the user-specified outlier fraction threshold ß was set to 0.01 such that 1% of the training target data are allowed to be classified as outlier or get rejected [12]. Class imbalance was mitigated by oversampling of the nontarget data sets through random sampling [44]. Performance was measured using the area under the receiver operating characteristic (ROC) curve (AUC), specificity, and F1-score [45-48]. The AUC, specificity, and F1-score were reported as the average (SD) of twenty times five-fold stratified cross-validation rounds. AUC is the result of integration (summation) of the ROC curve over a range of possible classification thresholds [49]. It is regarded as robust (insensitive) when it comes to the presence of data imbalance; however, it is impractical for real-world implementation because it is independent of a single threshold [48]. Specificity measures the ratio of correctly classified negative samples from the total number of available negative samples [50]. Thus, it depicts the proportion of infection days (nontarget samples) that are correctly classified as such to the total number of infection days (period). It is only used to examine how the model performs in regard to the nontarget class (infection days). F1-score is the harmonic mean of precision and recall, where the value ranges from 0 to 1, and high F1 scores depict high classification performance [45]. F1-score is considered appropriate when evaluating model performance with regard to one target class and in the presence of unbalanced data sets [10,46-48]. The models were further compared based on various criteria, which can contribute to the implementation of the models in real-world settings, including computation time, sample size, number of user-defined parameters, and sensitivity to outliers in the training data sets:

- Computation time: this characteristic defines the amount of time taken to train and test the model. Regarding personal use, response time is crucial for acceptance of the services by a wide range of users. Furthermore, with regard to the outbreak detection settings, this is an important parameter given that a system that uses data from many participants needs to have an acceptable response time. However, in real-world applications, the training phase can be performed in an offline mode, which makes the testing response time very crucial.
- Sample size: this characteristic specifies the minimum amount of training data required to generate an acceptable performance. This is an important factor given that the system relies on self-recorded data; it is difficult to accumulate a large set of data for an individual initially.
- Number of user-defined parameters: this characteristic defines the complexity of the model. It is simpler and less data are required to estimate a model with fewer parameters. This is an important factor because it is easier for an individual to implement the simple model compared with the complex model.
- Sensitivity to outliers in the training data sets: this characteristic defines how the model estimation is affected by outliers in the training set. This is a crucial characteristic because the model training depends on self-reported data, which are highly dependent on the accuracy of the user data registration. It is possible that the user might forget to report some infection incidence and hence might be considered as target data sets and be used as a training data set. Furthermore, errors incurred during manual registration of data can also affect model generalization.

### Data Collection and Ethical Declaration

The study protocol has been submitted to the Norwegian Regional Committees for Medical Health Research Ethics Northern Norway for evaluation and was found exempted from regional ethics review because it is outside the scope of medical research (reference number: 108435). Written consent was obtained, and the participants donated the data sets. All data from the participants were anonymized.

## Results

The models were evaluated based on two different versions of the same data set: raw and filtered. The input variables to the models were the average blood glucose levels and the ratio of total insulin (bolus)-to-total carbohydrate. The necessary computational time for both training and testing of the models was also estimated. A comparison of the classifiers was carried out taking into account their performance, necessary sample size for producing acceptable performance, and computational time. These models were further compared based on their theoretical guarantee provided for robustness to outliers in the target data set and based on their complexity. In addition, these classifiers were compared with the unsupervised version of some selected models.

### Model Evaluation

Model training and evaluations were carried out on an individual basis taking into account different characteristics of the data, specified time window or resolution (hourly and daily), and nature of the data (raw data and its smoothed version). For daily evaluation, we compared the performance of the models on raw data and its smoothed version with a 2-day moving average filter. For hourly evaluation, we compared the performance of the model on a smoothed version of the data set. The purpose of the comparison was to study the performance gain achieved by removing short-time noises from the data set through smoothing. The average and SD of AUC, specificity, and F1-score are computed and reported for each model. The top performing models from each category are highlighted in italics within each tables.

#### Semisupervised Models

The regular or normal days were labeled as the target class data set and the infection period as the nontarget class data set. Three groups of one-class classifiers were trained on the target class and tested on a data set containing both the target and the nontarget classes. In addition to the data characteristics stated above, resolution and data nature, the one-class classifier performance was also assessed taking into account the required sample object size to produce acceptable data description. In this direction, we consider four groups of sample size: 1 month, 2 months, 3 months, and 4 months data sets. In the model evaluation, the data set containing the infection period was presented during testing. The evaluation was carried out based on 20 times 5-fold stratified cross-validation. The performance of the model was reported as the average and SD of AUC, specificity, and F1-score of the rounds. A score plot of each model for both the hourly and the daily scenarios using the smoothed version of the data can be found in Multimedia Appendix 3, where the models were trained on random 120 regular or normal days of the patient year and tested over the whole year.

##### Daily

As can be seen in Tables 2 and 3 below (see also Multimedia Appendix 4), the performance of the models generally improves as the size of the sample increases. The models performed well with respect to the raw data sets; however, the performance significantly improved with the smoothed version of the data. The results indicate that the sample size greatly affects the model performance and that there is a larger variation in performance when the training data set is small. Generally, it can be seen that the models generalize well with the 3-month data set (90 sample objects) and further improve after 3 months. In general, on average, with both the raw and smoothed data sets, the boundary and domain-based method performed better with a small sample size. As the sample size increased, all the three groups produced comparable descriptions of the data. From each respective category, models such as V-SVM, K-NN, and K-means performed well across all the sample sizes.

###### First Case of Infection (Flu)

The boundary and domain-based method achieved a better description of the data with a small sample size when compared with the other two groups. However, as the sample size increased, all the three groups achieved relatively comparable descriptions of the data. Specific models such as V-SVM, K-NN, and K-means performed better from their respective group. Regarding the raw data, as seen in Table 2, all the models failed to generalize from the 1-month data set as compared with the large sample objects, that is, 3 months, which was expected:

1. From the boundary and domain-based method, V-SVM performed better in all the sample sizes and achieved comparable performance even with 60 objects and improved significantly afterward. SVDD produced a comparable description with higher sample sizes, that is, 3 months and later.
2. From the density-based method, K-NN performed better in all the sample sizes and achieved better performance even with 60 objects. Naïve Parzen produced comparable performance with higher sample sizes, that is, 3 months and later.
3. From the reconstruction-based method, K-means achieved better performance for all sample sizes.

Smoothing the data, as shown in Table 3, improved the model performance even with 30 sample objects:

1. From the boundary and domain-based method, V-SVM achieved better performance in all sample sizes.
2. From the density-based method, K-NN achieved better performance for all sample sizes, minimum covariance determinant (MCD) Gaussian produced a comparable description with 30 and 60 sample objects, and naïve Parzen achieved comparable description of the data with 4-month sample objects.
3. Regarding the reconstruction-based method, PCA achieved good performance with 30 and 60 sample objects, whereas K-means performed better with larger sample objects.

**Table 2:** Average (SD) of area under the receiver operating characteristic curve, specificity, F1-score for the raw data set (without smoothing), and different sample size. Fraction=0.01.

Models		1 month			2 months			3 months			4 months		
		AUC^a^, mean (SD)	Specificity, mean (SD)	F1, mean (SD)	AUC, mean (SD)	Specificity, mean (SD)	F1, mean (SD)	AUC, mean (SD)	Specificity, mean (SD)	F1, mean (SD)	AUC, mean (SD)	Specificity, mean (SD)	F1, mean (SD)
**Boundary and domain–based method**													
	SVDD^b^	90.7 (8.8)	71.7 (7.7)	73.6 (5.5)	93.4 (6.2)	81.7 (5.0)	87.4 (8.1)	96.4 (2.9)	87.8 (3.3)	91.3 (6.0)	94.6 (3.7)	81.7 (5.0)	90.0 (4.6)
	IncSVDD^c^	90.4 (8.9)	66.7 (7.5)	72.7 (4.9)	91.8 (5.9)	66.7 (7.5)	84.4 (3.2)	95.8 (2.9)	70.0 (7.1)	85.4 (1.2)	93.7 (3.6)	55 (10.7)	81.0 (2.7)
	V-SVM^d^	93.1 (6.0)	63 (10.6)	*78.9 (6.2)* ^e^	96.5 (2.3)	81.9 (4.7)	*90.7 (3.4)*	97.9 (1.5)	88.9 (0.0)	*94.1 (2.0)*	96.2 (2.3)	83.3 (0.0)	*91.7 (1.4)*
	NN^f^	74.2 (9.3)	38.3 (7.7)	61.0 (4.7)	89.5 (9.3)	20.0 (6.7)	70.0 (4.6)	90.1 (6.6)	11.1 (18)	69.2 (3.8)	92.8 (3.3)	33.3 (0.0)	75.1 (0.4)
	MST^g^	89.4 (8.1)	50.0 (0.0)	62.7 (6.6)	95.4 (5.6)	61.7 (7.7)	82.3 (5.9)	96.6 (2.7)	68.9 (4.5)	83.6 (4.7)	94.1 (2.8)	55.0 (7.7)	80.6 (2.3)
**Density-based method**													
	Gaussian	90.6 (7.1)	60.0 (8.2)	68.8 (8.4)	95.4 (4.6)	70.0 (6.7)	85.3 (4.6)	97.3 (2.5)	80.0 (4.5)	89.2 (3.3)	95.5 (3.2)	66.7 (0.0)	84.5 (2.0)
	MOG^h^	88.1 (9.9)	80.1 (17.3)	67.8 (16.4)	93.1 (7.1)	75.8 (14.8)	82.5 (10.1)	95.6 (3.4)	80.2 (7.5)	86.0 (6.7)	93.7 (3.9)	68.7 (11.6)	84.2 (5.7)
	MCD^i^ Gaussian	89.0 (8.5)	55.0 (7.7)	66.4 (9.0)	94.0 (4.6)	68.3 (5.0)	84.6 (6.3)	97.0 (2.7)	80.0 (4.5)	89.9 (2.4)	94.5 (3.2)	65.0 (5.0)	84.0 (3.2)
	Parzen	89.0 (9.2)	70.0 (6.7)	70.7 (5.9)	94.6 (4.9)	83.3 (0.0)	87.9 (6.3)	97.2 (2.4)	88.9 (0.0)	90.5 (5.9)	95.2 (2.9)	83.3 (0.0)	88.9 (3.3)
	Naïve Parzen	90.1 (7.6)	55 (10.7)	65.0 (5.0)	95.7 (3.9)	76.7 (8.2)	87.2 (3.5)	98.3 (1.4)	88.9 (0.0)	*93.6 (2.4)*	96.8 (2.1)	83.3 (0.0)	90.7 (2.0)
	K-NN^j^	91.8 (6.9)	50.0 (0.0)	66.0 (2.0)	95.6 (3.1)	81.7 (5.0)	*90.9 (3.2)*	97.9 (1.6)	88.9 (0.0)	93.5 (3.7)	97.0 (2.2)	83.3 (0.0)	*92.0 (1.0)*
	LOF^k^	88.5 (6.1)	66.7 (7.5)	*72.7 (4.9)*	97.0 (1.9)	71.7 (7.7)	86.1 (2.4)	96.8 (2.8)	78.9 (3.3)	88.7 (2.8)	92.6 (4.8)	50.0 (0.0)	79.3 (2.6)
**Reconstruction-based method**													
	PCA^l^	87.8 (11.9)	50.0 (7.5)	62.4 (8.5)	93.5 (6.2)	51.7 (5.0)	78.2 (4.1)	93.6 (4.7)	60 (10.2)	81.8 (4.4)	91.3 (5.2)	46.7 (6.7)	78.7 (2.3)
	Auto-encoder	82.2 (12.0)	57.9 (15.3)	64.7 (12.0)	88.2 (9.5)	61.6 (14.0)	81.4 (7.1)	93.4 (5.7)	74.4 (11)	86.4 (5.9)	88.4 (8.8)	61.3 (14.3)	82.7 (5.7)
	SOM^m^	86.9 (9.4)	78.3 (13.3)	66.7 (16.9)	92.8 (7.3)	64.2 (12.4)	80.9 (7.0)	95.8 (3.7)	80.1 (6.3)	86.9 (5.5)	92.2 (4.1)	76.5 (9.0)	87.5 (4.5)
	K-means	91.8 (6.9)	65.0 (9.0)	*71.8 (5.1)*	96.0 (2.4)	83.3 (0.0)	*91.5 (2.8)*	97.6 (1.6)	88.9 (0.0)	*93.5 (3.7)*	96.2 (2.2)	83.3 (0.0)	*91.5 (1.6)*

^a^AUC: area under the receiver operating characteristic curve.

^b^SVDD: support vector data description.

^c^IncSVDD: incremental support vector data description.

^d^V-SVM: one-class support vector machine.

^e^Italicized values indicates the top performing models.

^f^NN: nearest neighbor.

^g^MST: minimum spanning tree.

^h^MOG: mixture of Gaussian.

^i^MCD: minimum covariance determinant.

^j^K-NN: K-nearest neighbor.

^k^LOF: local outlier factor.

^l^PCA: principal component analysis.

^m^SOM: self-organizing maps.

**Table 3:** Average of area under the receiver operating characteristic curve, specificity, and F1-score for smoothed version of the data with a 2-day moving average filter and different sample size. Fraction=0.01.

Models		1 month			2 months			3 months			4 months		
		AUC^a^, mean (SD)	Specificity	F1	AUC^a^, mean (SD)	Specificity	F1	AUC^a^, mean (SD)	Specificity	F1	AUC^a^, mean (SD)	Specificity	F1
**Boundary and domain–based method**													
	SVDD^b^	99.6 (1.3)	100 (0.0)	93.6 (15.2)	100 (0.0)	100 (0.0)	94.8 (10.1)	100 (0.0)	100 (0.0)	97.0 (4.1)	100 (0.0)	100 (0.0)	96.9 (4.0)
	IncSVDD^c^	99.6 (1.3)	100 (0.0)	93.6 (15.2)	100 (0.0)	100 (0.0)	97.1 (6.3)	100 (0.0)	100 (0.0)	97.6 (4.1)	100 (0.0)	100 (0.0)	98.3 (2.8)
	V-SVM^d^	100 (0.0)	99.5 (2.9)	*98.9 (3.2)* ^e^	100 (0.0)	100 (0.0)	*99.1 (2.6)*	100 (0.0)	100 (0.0)	*99.4 (1.7)*	100 (0.0)	100 (0.0)	*99.6 (1.2)*
	NN^f^	98.1 (3.9)	58.3 (15.4)	72.3 (9.9)	86.9 (12.5)	16.7 (22.4)	70.5 (5.3)	88.1 (6.5)	54.4 (22.5)	80.0 (8.6)	92.4 (5.3)	8.3 (17.1)	69.0 (4.8)
	MST^g^	98.5 (2.4)	85.0 (5.0)	85.5 (2.1)	99.7 (0.8)	100 (0.0)	97.1 (6.3)	99.9 (0.4)	97.8 (4.5)	97.2 (4.0)	99.7 (0.8)	100 (0.0)	97.0 (7.9)
**Density-based method**													
	Gaussian	100 (0.0)	98.3 (5.0)	92.1 (15.2)	100 (0.0)	100 (0.0)	97.1 (6.3)	99.8 (0.7)	100 (0.0)	97.6 (4.1)	99.4 (1.7)	100 (0.0)	97.0 (7.9)
	MOG^h^	98.6 (3.2)	99.8 (1.7)	88.5 (16.8)	99.6 (1.2)	100 (0.0)	92.2 (11.1)	99.7 (0.7)	99.8 (1.4)	94 (10.3)	99.3 (2.0)	99.9 (1.2)	94.4 (11.8)
	MCD^i^ Gaussian	98.9 (2.2)	91.7 (8.4)	*90.9 (7.7)*	100 (0.0)	100 (0.0)	*98.0 (6.0)*	99.5 (1.1)	96.7 (5.1)	96.6 (5.9)	99.4 (1.7)	88.3 (7.7)	92.0 (6.8)
	Parzen	99.6 (1.3)	100 (0.0)	87.7 (17.0)	100 (0.0)	100 (0.0)	95.1 (8.0)	100 (0.0)	100 (0.0)	94.6 (9.8)	99.9 (0.4)	100 (0.0)	94.6 (12.3)
	Naïve Parzen	99.2 (2.5)	100 (0.0)	94.7 (11.1)	100 (0.0)	100 (0.0)	93.8 (11.0)	99.6 (1.1)	100 (0.0)	97.5 (5.0)	100 (0.0)	100 (0.0)	*98.7 (2.7)*
	K-NN^j^	98.1 (3.9)	68.3 (5.0)	75.2 (4.3)	100 (0.0)	100 (0.0)	*98.0 (6.0)*	100 (0.0)	100 (0.0)	*98.8 (3.8)*	100 (0.0)	100 (0.0)	*97.7 (4.7)*
	LOF^k^	98.6 (2.9)	75.0 (13.5)	80.2 (10.8)	100 (0.0)	100 (0.0)	*98.0 (6.0)*	100 (0.0)	100 (0.0)	96.9 (5.0)	99.7 (0.8)	100 (0.0)	97.4 (7.9)
**Reconstruction-based method**													
	PCA^l^	98.9 (2.2)	85.0 (5.0)	*85.5 (2.1)*	99.2 (1.3)	85.0 (5.0)	*91.4 (2.7)*	98.6 (1.9)	88.9 (0.0)	92.2 (6.0)	97.8 (2.2)	83.3 (0.0)	89.1 (9.7)
	Auto-encoder	97.4 (6.0)	89.1 (13.0)	86.0 (14.2)	98.5 (3.2)	94.5 (9.6)	91.8 (9.4)	99.2 (2.4)	93.7 (10.2)	93.7 (8.3)	98.6 (3.8)	94.4 (9.5)	93.7 (9.7)
	SOM^m^	99.3 (1.9)	99.9 (1.2)	84.7 (19.8)	99.8 (0.7)	100 (0.0)	91.4 (9.6)	99.9 (0.3)	100 (0.0)	95.2 (7.9)	99.6 (1.3)	100 (0.0)	93.4 (12.1)
	K-means	99.2 (2.5)	85.0 (11.7)	87.0 (10.4)	100 (0.0)	100 (0.0)	97.1 (6.3)	100 (0.0)	100 (0.0)	*98.8 (3.8)*	100 (0.0)	100 (0.0)	*99.2 (2.5)*

^a^AUC: area under the receiver operating characteristic curve.

^b^SVDD: support vector data description.

^c^IncSVDD: incremental support vector data description.

^d^V-SVM: one-class support vector machine.

^e^Italicized values indicates the top performing models.

^f^NN: nearest neighbor.

^g^MST: minimum spanning tree.

^h^MOG: mixture of Gaussian.

^i^MCD: minimum covariance determinant.

^j^K-NN: K-nearest neighbor.

^k^LOF: local outlier factor.

^l^PCA: principal component analysis.

^m^SOM: self-organizing maps.

###### Second Case of Infection (Flu)

The boundary and domain-based method achieved better performance with a small sample size compared with the density and reconstruction-based methods. However, as the sample size increased, all the three groups achieved comparable performance. The detailed numerical values of comparison are given in Multimedia Appendix 4. Specific models such as V-SVM**,** K-NN, and K-means performed better from their respective group. Regarding the raw data, all the models failed to generalize from the 1-month data set as compared with the higher sample objects, that is, 3 months (Multimedia Appendix 4):

1. From the boundary and domain-based method, SVDD, MST, and incremental support vector data description (incSVDD) performed better with a larger sample object, and V-SVM achieved better description with 30 sample objects.
2. From the density-based method, all the models exhibited similar performance. Naïve Parzen and K-NN, with only 60 sample objects, achieved comparable performance with the higher sample objects.
3. From the reconstruction-based method, K-means achieved better performance for all sample sizes.

Smoothing the data significantly improved the performance of the model even with 30 objects, compared with the raw data (Multimedia Appendix 4):

1. From the boundary and domain-based method, the V-SVM achieved higher performance in all the sample sizes.
2. From the density-based method, LOF achieved better description with small sample objects, and K-NN produced better description with all the sample sizes. Gaussian families achieved improved and comparable performance with increased sample objects. Among them, K-NN with only 60 objects achieved comparable performance with larger sample objects.
3. Regarding the reconstruction-based method, K-means and SOM achieved better performance, whereas K-means performed better in all the sample sizes.

###### Third Case of Infection (Flu)

The boundary and domain-based method achieved better performance with a small sample size compared with the density and reconstruction-based methods. However, as the sample size increased, all the three groups produced comparable descriptions. The detailed numerical values of comparison are given in Multimedia Appendix 4. Specific models such as V-SVM, MST, LOF, and PCA performed better from their respective group. Regarding the raw data, surprisingly, in contrast to the previous two infection cases, all the models achieved higher generalization from the 1-month data set (Multimedia Appendix 4):

1. From the boundary and domain-based method, SVDD, V-SVM, MST, and incSVDD performed better in all the cases, with MST achieving better performance.
2. From the density-based method, normal and MCD Gaussian achieved better description of the data with 1-month sample objects. K-NN and LOF performed better with sample sizes larger than 1-month sample objects, and LOF outperformed all sample sizes. The LOF with only 60 objects achieved comparable performance with the higher sample objects.
3. From the reconstruction-based method, PCA produced better description for all sample sizes, whereas K-means and SOM achieved comparable performance with sample size larger than 1-month sample objects.

Smoothing the data allowed the models to generalize well and significantly improved the performance of the model even with 30 objects, compared with the raw data (Multimedia Appendix 4):

1. From the boundary and domain-based method, the V-SVM and MST achieved higher performance in all the sample sizes, whereas V-SVM outperformed all the models.
2. From the density-based method, the Gaussian families, LOF, and K-NN achieved better performance, whereas LOF achieved better performance in all sample sizes.
3. Regarding the reconstruction-based method, K-means and PCA achieved better performance, whereas PCA performed better in all the sample sizes.

###### Fourth Case of Infection (Flu)

The boundary and domain-based method achieved better performance with small sample sizes compared with the density and reconstruction-based methods. All the three groups improved with increasing sample size. The detailed numerical values of comparison are given in Multimedia Appendix 4. Specific models such as V-SVM, LOF, and K-means performed better from their respective group. Regarding the raw data, surprisingly, in contrast to all the previous three infection cases, all the models achieved higher generalization from the 1-month data set (Multimedia Appendix 4):

1. From the boundary and domain-based method, SVDD, V-SVM, and incSVDD performed better for all the sample sizes.
2. From the density-based method, MCD Gaussian performed better with a 1-month sample size, and all the models produced comparable descriptions as the sample size increased, whereas the LOF performed better for all the sample sizes.
3. From the reconstruction-based method, PCA performed relatively better for all the sample sizes, and K-means and SOM achieved comparable performance with a larger sample size.

Smoothing the data significantly improved the model performance even with 30 objects compared with the raw data (Multimedia Appendix 4):

1. From the boundary and domain-based method, the V-SVM achieved higher performance in all the sample sizes. As the sample size increased, the incSVDD and MST achieved comparable performance.
2. From the density-based method, K-NN and LOF produced better descriptions with a 1-month sample size. K-NN performed better in almost all sample sizes.
3. From the reconstruction-based method, K-means achieved better performance for all sample sizes.

##### Hourly

As can be seen in Table 4 (see also Multimedia Appendix 4), the performance of the model generally improved as more training sample data were presented. The models produced comparable performance even with the 1-month data set compared with the daily scenario. This is mainly because of the presence of more samples per day (24 samples per day), which enables the models to reach a better generalization. Generally, the results indicate that the models generalize well after 2 months. Both the boundary and domain-based method and reconstruction-based method achieved better performance even with a 1-month sample size. However, the density-based method suffers from large variation with 1-month training samples. In general, the boundary and domain-based method performed better in all the infection cases compared with the other two methods. In addition, specific models such as V-SVM, K-NN, and K-means performed well from their respective groups.

###### First Case of Infection (Flu)

The boundary and domain-based method achieved better performance compared with the density and reconstruction-based methods. As can be seen in Table 4, the boundary and domain-based method achieved better generalization from the 1-month data set. Specific models such as V-SVM, K-NN, and K-means performed better from their respective group:

1. From the boundary and domain-based method, V-SVM achieved better description in all sample sizes, whereas SVDD, incSVDD, and V-SVM achieved comparable performance with a larger sample size.
2. From the density-based method, Gaussian families and naïve Parzen performed better at large sample sizes, whereas K-NN and LOF achieved better performance in all the sample sizes. K-NN outperformed all the models.
3. From the reconstruction-based method, K-means performed better in all the sample sizes, and all the other models performed better with larger sample sizes.

**Table 4:** Average (SD) of area under the receiver operating characteristic curve, specificity, F1-score for the smoothed version of the data with a 48-hour moving average filter and different sample size. Fraction=0.01.

Models		1 month			2 months			3 months			4 months		
		AUC^a^, mean (SD)	Specificity	F1	AUC^a^, mean (SD)	Specificity	F1	AUC^a^, mean (SD)	Specificity	F1	AUC^a^, mean (SD)	Specificity	F1
**Boundary and domain–based method**													
	SVDD^b^	97.6 (1.9)	83.2 (3.4)	85.8 (1.7)	97.8 (1.2)	85.7 (5.0)	90.5 (9.6)	97.7 (1.2)	90.4 (5.1)	94.2 (2.9)	98.1 (0.9)	91.0 (3.7)	96.8 (0.9)
	IncSVDD^c^	97.4 (1.9)	84.5 (2.8)	86.8 (1.9)	97.7 (1.2)	86.7 (2.0)	93.9 (1.0)	97.5 (1.2)	88.5 (1.5)	96.0 (1.1)	97.9 (0.9)	88.9 (1.2)	*97.1 (0.7)*
	V-SVM^d^	98.1 (2.1)	84.5 (1.1)	*90.5 (1.1)* ^e^	99.0 (1.1)	92.6 (0.0)	*96.1 (1.3)*	99.5 (0.6)	93.8 (0.5)	*96.9 (1.4)*	99.4 (0.4)	94.2 (0.0)	97.1 (1.3)
	NN^f^	84.8 (6.0)	75.9 (4.5)	74.8 (6.0)	89.3 (2.2)	76.5 (4.1)	87.1 (3.3)	89.0 (4.0)	77.5 (3.9)	89.3 (4.4)	90.2 (4.7)	77.5 (3.8)	91.4 (6.4)
	MST^g^	90.5 (3.1)	85.4 (3.9)	67.6 (14.5)	94.4 (2.0)	85.7 (4.0)	85.1 (7.0)	94.7 (2.4)	88.8 (3.5)	87.8 (8.5)	95.8 (2.2)	88.8 (3.0)	90.9 (5.9)
**Density–based method**													
	Gaussian	98.1 (2.2)	79.8 (4.9)	83.9 (2.7)	99.5 (0.9)	90.1 (1.7)	95.2 (1.8)	99.6 (0.7)	92.9 (1.3)	97.1 (2.5)	99.5 (0.5)	92.2 (1.0)	97.7 (1.1)
	MOG^h^	95.8 (3.6)	82.7 (4.3)	83.7 (5.0)	98.3 (1.5)	86.2 (2.7)	92.3 (2.7)	98.7 (1.4)	88.7 (4.6)	94.7 (3.5)	98.6 (1.6)	88.2 (3.1)	95.3 (3.2)
	MCD^i^ Gaussian	98.6 (2.1)	75.3 (6.9)	81.3 (2.5)	99.6 (0.9)	89.6 (1.9)	95.0 (1.8)	99.6 (0.7)	92.5 (1.8)	97.0 (2.3)	99.6 (0.4)	92.0 (1.2)	97.7 (1.1)
	Parzen	91.9 (2.9)	93.6 (2.0)	63.4 (16.5)	96.2 (2.3)	94.4 (2.0)	81.6 (10.2)	96.6 (2.6)	94.8 (1.7)	84.2 (9.5)	97.4 (2.2)	95.6 (1.2)	87.9 (7.1)
	Naïve Parzen	94.8 (3.7)	76.4 (5.6)	77.6 (7.9)	98.7 (1.2)	85.2 (3.3)	91.8 (2.9)	99.1 (1.1)	89.1 (3.8)	94.8 (2.5)	98.9 (0.9)	89.7 (2.4)	96.2 (1.6)
	K-NN^j^	97.1 (3.4)	78.8 (2.0)	*84.2 (2.1)*	99.1 (1.0)	92.9 (0.7)	*96.0 (1.8)*	99.6 (0.4)	93.8 (0.7)	*97.3 (1.9)*	99.5 (0.3)	94.0 (0.6)	*98.2 (0.9)*
	LOF^k^	96.9 (3.5)	78.3 (3.0)	84.2 (2.4)	99.2 (1.1)	91.9 (0.9)	*96.0 (1.8)*	99.6 (0.5)	93.7 (0.8)	97.3 (2.1)	99.5 (0.4)	93.1 (0.4)	97.8 (1.2)
**Reconstruction–based method**													
	PCA^l^	97.1 (3.4)	63.9 (8.8)	75.4 (0.3)	99.4 (1.2)	76.4 (6.6)	90.2 (1.1)	99.1 (1.3)	75.1 (6.8)	92.4 (1.1)	98.9 (1.2)	69.1 (4.1)	93.1 (0.8)
	Auto-encoder	92.0 (4.8)	79.5 (7.6)	78.9 (8.3)	96.2 (2.6)	83.1 (7.2)	91.1 (3.9)	96.3 (3.2)	84.3 (7.7)	92.7 (5.0)	96.7 (3.0)	84.0 (8.0)	94.6 (4.4)
	SOM^m^	94.1 (2.3)	82.2 (3.3)	82.6 (4.9)	95.6 (1.1)	82.9 (3.1)	91.6 (1.9)	94.8 (2.3)	83.4 (5.8)	92.3 (4.1)	95.5 (1.9)	84.1 (3.8)	94.3 (3.8)
	K-means	97.3 (3.2)	80.9 (2.5)	*85.5 (2.5)*	98.9 (1.1)	92.6 (0.7)	*95.8 (1.8)*	99.3 (0.6)	92.9 (0.7)	*97.3 (1.4)*	99.4 (0.4)	94.1 (0.2)	*98.1 (1.1)*

^a^AUC: area under the receiver operating characteristic curve.

^b^SVDD: support vector data description.

^c^IncSVDD: incremental support vector data description.

^d^V-SVM: one-class support vector machine.

^e^Italicized values indicates the top performing models.

^f^NN: nearest neighbor.

^g^MST: minimum spanning tree.

^h^MOG: mixture of Gaussian.

^i^MCD: minimum covariance determinant.

^j^K-NN: K-nearest neighbor.

^k^LOF: local outlier factor.

^l^PCA: principal component analysis.

^m^SOM: self-organizing maps.

###### Second Case of Infection (Flu)

The boundary and domain-based method and reconstruction-based method achieved better performance for all sample sizes compared with the density-based method. Specifically, the boundary and domain-based method achieved better generalization from the 1-month data set. The detailed numerical values of comparison are given in Multimedia Appendix 4. Specific models such as V-SVM, K-NN, and K-means performed better from their respective group:

1. From the boundary and domain-based method, V-SVM achieved better description for all the sample sizes, and SVDD, NN, and incSVDD improved with larger training sample size; however, V-SVM outperformed all the models for all the sample sizes.
2. From the density-based method, normal and MCD Gaussian performed better with the 1- and 2-month sample sizes, and models such as K-NN performed better on all the sample sizes, whereas naïve Parzen outperformed all the models with the 3- and 4-month data sets.
3. From the reconstruction-based method, K-means produced better description for all the sample sizes and the auto-encoder and SOM performed better with larger sample sizes.

###### Third Case of Infection (Flu)

Generally, in comparison, all the groups performed better at large training sample sizes; however, the boundary and domain-based method achieved better performance with small training sample sizes. It achieved comparable generalization from the 1-month data set. The detailed numerical values of comparison are given in Multimedia Appendix 4. Specific models such as V-SVM, families that utilize nearest neighbor distance (K-NN and LOF), and PCA performed better from their respective group:

1. From the boundary and domain-based method, SVDD, NN, MST, incSVDD, and V-SVM achieved better performance at larger training sample sizes, whereas V-SVM outperformed all the models for all the sample sizes.
2. From the density-based method, the Gaussian families, K-NN, LOF, and naïve Parzen achieved better performance at larger training sample sizes, whereas K-NN and LOF outperformed all the models for all the sample sizes.
3. From the reconstruction-based method, K-means, PCA, auto-encoder, and SOM achieved better performance at larger training sample sizes, whereas PCA performed better for all sample sizes.

###### Fourth Case of Infection (Flu)

Generally, in comparison, all the group performed better at large training sample size; however, the boundary and domain-based method achieved better performance with small training sample sizes, for example, 1-month data set. It achieved comparable generalization from the 1-month data set. The detailed numerical values of comparison are given in Multimedia Appendix 4. Specific models such as V-SVM, Gaussian families (Gaussian, MOG, and MCD Gaussian), and PCA performed better from their respective groups:

1. From the boundary and domain-based method, NN, incSVDD, and V-SVM achieved better performance at larger training sample sizes, whereas V-SVM outperformed all the models for all the sample sizes.
2. From the density-based method, Gaussian families, K-NN, LOF, and naïve Parzen achieved better performance at larger training sample sizes, whereas Gaussian families outperformed all the models for all the sample sizes.
3. From the reconstruction-based method, K-means, SOM, auto-encoder, and PCA achieved better performance at larger training sample sizes, whereas PCA performed better for all sample sizes.

##### Average Performance Across all the Infection Cases

The average performances of the models across all the infection cases for different sample sizes, levels of data granularity (hourly and daily), and nature of data (raw and smoothed) are shown in Tables 5-7. In general, the boundary and domain-based method performed better than the other two groups in both daily and hourly smoothed data sets; however, all the groups achieved comparable performance with respect to the daily raw data set. Specific models such as V-SVM, K-NN, and K-means performed better in all these circumstances.

###### Daily Raw Data Set

Regarding the daily raw data set, as shown in Table 5, specific models such as V-SVM, MCD Gaussian, K-NN, and K-means produced relatively better descriptions of the 1-month data. For the 2-month sample size, models such as incSVDD, K-NN, LOF, and K-means achieved better performance. For the 3-month sample size, SVDD, incSVDD, V-SVM, Gaussian, MCD Gaussian, K-NN, LOF, and K-means produced comparable descriptions. As expected, SVDD and most of the density-based method improved with larger training sizes. For the 4-month sample size, almost all the models produced much improved performance. In the group comparison, all three groups produced comparable descriptions in all the sample sizes.

**Table 5:** Average performance of each model across all the infection cases for the daily raw data set (without smoothing) and different sample sizes. Fraction=0.01.

Models		1 month			2 months			3 months			4 months		
		AUC^a^, mean (SD)	Specificity	F1	AUC^a^, mean (SD)	Specificity	F1	AUC^a^, mean (SD)	Specificity	F1	AUC^a^, mean (SD)	Specificity	F1
**Boundary and domain-based method**													
	SVDD^b^	87.1 (11)	66.0 (13.5)	74.8 (9.5)	91.7 (7.3)	61.7 (10.6)	84.1 (5.5)	93.3 (4.6)	67.3 (10.5)	86.2 (4.4)	91.4 (4.3)	61.7 (10.6)	*85.7 (4.1)* ^c^
	IncSVDD^d^	85.2 (11)	63.0 (4.6)	74.7 (10.4)	90.5 (8.5)	57.9 (11)	*83.8 (3.6)*	92.8 (5.1)	62.8 (10.9)	84.9 (3.2)	90.8 (4.4)	55.0 (11.7)	83.5 (3.7)
	V-SVM^e^	91.5 (8.0)	55.7 (7.0)	*77.4 (6.4)*	92.2 (5.1)	60.6 (5.0)	82.8 (4.5)	94.2 (3.8)	66.9 (6.1)	*86.6 (3.5)*	93.8 (4.1)	63.1 (11.9)	84.5 (5.1)
	NN^f^	73.4 (12)	31.3 (6.5)	65.0 (5.4)	72.1 (11.9)	25.0 (9.6)	75.7 (3.7)	70.8 (11.2)	8.6 (17.6)	72.0 (4.7)	70.0 (9.0)	16.0 (14.4)	75.7 (3.4)
	MST^g^	82.4 (8.7)	52.1 (0.0)	71.2 (6.1)	82.6 (9.1)	50.4 (9.0)	82.0 (5.1)	84.0 (6.3)	56.2 (9.3)	82.9 (3.5)	84.2 (6.6)	50.0 (11.4)	82.6 (2.7)
**Density-based method**													
	Gaussian	91.5 (9.9)	56.9 (7.7)	72.9 (7.8)	93.6 (6.1)	58.8 (10.9)	84.0 (4.0)	95.1 (4.3)	65.3 (10.6)	86.3 (3.2)	95.0 (3.5)	57.9 (10.3)	84.6 (3.2)
	MOG^h^	89.9 (12)	69.2 (11.9)	71.3 (14.3)	91.7 (6.1)	64.1 (14.0)	83.8 (6.8)	94.0 (4.4)	67.0 (11.4)	85.0 (5.6)	94.5 (3.7)	61.6 (12.6)	84.9 (5.1)
	MCD^i^ Gaussian	90.8 (9.1)	54.0 (5.5)	*72.0 (6.8)*	93.1 (6.0)	58.0 (8.1)	84.1 (4.3)	95.3 (4.2)	65.3 (10.6)	86.4 (3.0)	94.8 (3.5)	57.9 (10.6)	84.9 (3.0)
	Parzen	89.7 (10)	59.6 (8.3)	70.6 (9.4)	91.7 (6.5)	62.1 (10.3)	83.9 (5.3)	93.9 (5.0)	68.7 (11.2)	85.6 (5.4)	94.3 (3.8)	66.1 (12.7)	86.1 (3.8)
	Naïve Parzen	88.1 (8.7)	54.2 (6.5)	69.1 (9.6)	90.2 (7.1)	60.4 (11.2)	83.7 (4.9)	91.9 (5.5)	66.5 (12.8)	86.6 (4.4)	92.8 (4.7)	64.6 (10.0)	*86.9 (3.4)*
	K-NN^j^	91.1 (7.8)	52.9 (5.1)	71.6 (7.9)	91.6 (5.0)	61.1 (11.3)	*85.9 (3.1)*	94.8 (4.8)	66.9 (11.2)	*87.1 (3.2)*	95.0 (3.8)	62.1 (10.3)	*86.5 (3.3* **)**
	LOF^k^	89.2 (8.9)	56.3 (3.9)	73.0 (8.6)	92.4 (6.0)	59.2 (11.1)	84.9 (2.8)	94.0 (4.8)	64.4 (11.4)	86.2 (2.8)	93.7 (4.3)	53.8 (10.3)	83.8 (2.5)
**Reconstruction-based method**													
	PCA^l^	87.6 (8.8)	58.8 (4.6)	73.7 (8.3)	90.2 (6.4)	55.0 (6.8)	82.7 (4.5)	91.4 (4.9)	59.7 (6.2)	84.1 (3.2)	90.5 (4.5)	53.8 (7.2)	83.6 (2.9)
	Auto-encoder	83.6 (14)	58.3 (17.7)	71.0 (12.5)	84.6 (12.5)	53.1 (20.0)	82.1 (7.0)	88.4 (10.0)	57.7 (21.5)	83.3 (6.8)	88.5 (10.6)	52.3 (21.0)	83.2 (5.8)
	SOM^m^	85.6 (12)	63.4 (10.3)	72.7 (11.7)	87.6 (7.2)	57.1 (10.2)	81.6 (5.8)	93.5 (5.4)	64.4 (8.5)	84.8 (4.0)	94.7 (4.0)	59.0 (5.8)	85.0 (3.1)
	K-means	94.2 (7.6)	57.2 (7.6)	*73.1 (7.1)*	93.7 (6.2)	62.2 (10.5)	*85.4 (4.2)*	96.0 (4.4)	67.6 (10.3)	*87.4 (3.1)*	95.8 (3.9)	62.1 (10.3)	*86.5 (2.9)*

^a^AUC: area under the receiver operating characteristic curve.

^b^SVDD: support vector data description.

^c^Italicized values indicates the top performing models.

^d^IncSVDD: incremental support vector data description.

^e^V-SVM: one-class support vector machine.

^f^NN: nearest neighbor.

^g^MST: minimum spanning tree.

^h^MOG: mixture of Gaussian.

^i^MCD: minimum covariance determinant.

^j^K-NN: K-nearest neighbor.

^k^LOF: local outlier factor.

^l^PCA: principal component analysis.

^m^SOM: self-organizing maps.

###### Daily Smoothed Data Set

Regarding the daily smoothed data set, as shown in Table 6, almost all models achieved excellent performance and much improved data description compared with the daily raw data set. As shown in Table 6, specific models such as V-SVM, K-NN, and K-means produced excellent descriptions of the data for all the sample sizes; however, V-SVM achieved superior performance compared with these models. In the group comparison, the boundary and domain-based method produced excellent description of the data for all sample sizes.

**Table 6:** Average performance of each model across all the infection cases for the daily smoothed data set (with filter) and different sample size. Fraction=0.01.

Models		1 month				2 months				3 months				4 months		
		AUC^a^, mean (SD)	Specificity	F1	AUC^a^, mean (SD)		Specificity	F1	AUC^a^, mean (SD)		Specificity	F1	AUC^a^, mean (SD)		Specificity	F1
**Boundary and domain-based method**																
	SVDD^b^	99.9 (0.7)	100 (0.0)	94.1 (14.2)	100 (0.0)		100 (0.0)	96.1 (7.6)	100 (0.0)		100 (0.0)	96.5 (6.5)	100 (0.0)		100 (0.0)	97.9 (3.9)
	**I**ncSVDD^c^	99.9 (0.7)	100 (0.0)	94.1 (14.2)	100 (0.0)		100 (0.0)	96.9 (6.5)	100 (0.0)		100 (0.0)	97.3 (5.9)	100 (0.0)		100 (0.0)	98.6 (2.9)
	V-SVM^d^	100 (0.0)	100 (0.0)	*99.1 (3.2)* ^e^	100 (0.0)		100 (0.0)	*99.1 (2.9)*	100 (0.0)		100 (0.0)	*99.4 (1.9)*	100 (0.0)		100 (0.0)	*99.5 (1.5)*
	NN^f^	90.1 (14.5)	40.0 (30.5)	69.5 (13.2)	88.9 (9.9)		33.1 (22.6)	78.4 (6.8)	89.2 (7.9)		33.6 (14.6)	77.7 (5.3)	90.5 (6.8)		23.5 (18.6)	77.1 (5.7)
	MST^g^	98.9 (3.6)	85 (6.1)	86.7 (9.4)	99.8 (0.7)		96.7 (3.4)	95.1 (6.2)	99.9 (0.2)		98.9 (4.1)	98.0 (3.5)	99.9 (0.5)		100 (0.0)	98.0 (5.4)
**Density-based method**																
	Gaussian	99.2 (5.1)	92.6 (9.0)	87.2 (15.2)	99.5 (2.5)		96.7 (7.5)	94.8 (10.4)	99.9 (0.4)		100 (0.0)	98.1 (4.9)	99.8 (0.8)		100 (0.0)	98.3 (5.9)
	MOG^h^	98.8 (5.4)	92.9 (8.6)	85.2 (17.1)	99.4 (2.6)		97.0 (5.4)	92.1 (11.6)	99.9 (0.4)		99.9 (0.7)	95.4 (7.8)	99.8 (1.0)		99.9 (0.6)	96.4 (7.7)
	MCD^i^ Gaussian	98.4 (5.6)	86.6 (8.8)	86.6 (11.9)	99.3 (2.7)		90.0 (8.7)	93.4 (8.1)	99.8 (0.5)		99.2 (2.6)	98.0 (5.3)	99.8 (0.9)		97.1 (3.9)	97.0 (5.5)
	Parzen	99.2 (3.5)	100 (0.0)	90.8 (16.4)	99.9 (0.4)		100 (0.0)	93.7 (9.8)	100 (0.0)		100 (0.0)	93.6 (8.9)	99.9 (0.3)		100 (0.0)	95.8 (8.2)
	Naïve Parzen	99.8 (1.2)	100 (0.0)	94.4 (14.6)	100 (0.0)		100 (0.0)	96.1 (7.9)	99.9 (0.5)		100 (0.0)	97.4 (5.6)	100 (0.0)		100 (0.0)	98.2 (4.2)
	K-NN^j^	99.5 (2.0)	91.6 (3.6)	*90.7 (9.6)*	99.9 (0.4)		100 (0.0)	*98.3 (4.9)*	100 (0.0)		100 (0.0)	*98.4 (5.1)*	100 (0.0)		100 (0.0)	*98.8 (3.6)*
	LOF^k^	99.6 (1.5)	93.3 (7.3)	92.4 (10.6)	99.9 (0.5)		99.2 (3.4)	97.1 (7.3)	99.9 (0.2)		98.6 (2.8)	97.4 (4.5)	99.9 (0.4)		100 (0.0)	98.2 (5.9)
**Reconstruction-based method**																
	PCA^l^	93.8 (6.7)	82.0 (7.3)	83.8 (10.4)	91.3 (4.3)		77.9 (7.3)	89.3 (8.7)	88.7 (5.9)		76.3 (8.6)	89.5 (5.3)	90.7 (3.6)		76.2 (8.6)	89.0 (6.9)
	Auto-encoder	97.0 (8.1)	91.6 (14.6)	87.7 (16.0)	98.1 (5.4)		92.6 (15.3)	92.0 (10.7)	98.6 (4.6)		92.8 (14.8)	94.0 (8.3)	98.7 (4.0)		92.7 (15.8)	94.9 (7.7)
	SOM^m^	99.1 (3.2)	99.9 (0.6)	85.2 (20.5)	99.8 (0.7)		100 (0.0)	88.9 (16.1)	99.9 (0.2)		100 (0.0)	94.6 (8.0)	99.8 (0.6)		100 (0.0)	95.9 (8.1)
	K-means	99.8 (1.2)	96.2 (6.0)	*93.2 (12.7)*	100 (0.0)		100 (0.0)	*97.8 (5.6)*	100 (0.0)		100 (0.0)	*98.0 (5.6)*	100 (0.0)		100 (0.0)	*99.0 (2.9)*

^a^AUC: area under the receiver operating characteristic curve.

^b^SVDD: support vector data description.

^c^IncSVDD: incremental support vector data description.

^d^V-SVM: one-class support vector machine.

^e^Italicized values indicates the top performing models.

^f^NN: nearest neighbor.

^g^MST: minimum spanning tree.

^h^MOG: mixture of Gaussian.

^i^MCD: minimum covariance determinant.

^j^K-NN: K-nearest neighbor.

^k^LOF: local outlier factor.

^l^PCA: principal component analysis.

^m^SOM: self-organizing maps.

###### Hourly Smoothed Data Set

Regarding the hourly smoothed data set, as shown in Table 7, almost all the models failed to produce acceptable data description from the 1-month sample size except V-SVM, which achieved the best description. The high variability between the performance of the models with the 1-month hourly data set could be associated with the high data granularity, and, in fact, the models require more data sets to capture the high variability among the data objects. Models such as V-SVM, MCD Gaussian, and K-means achieved superior performance from their respective groups. In general, V-SVM outperformed in all the sample sizes. The density and reconstruction-based models improved with larger sample size. In the group comparison, the boundary and domain-based method produced better description in all the sample sizes, and the density and reconstruction-based method achieved equivalent performance with larger sample sizes.

**Table 7:** Average performance of each model across all the infection cases for the hourly data set with smoothing and different sample size. Fraction=0.01.

Models		1 month			2 months			3 months			4 months		
		AUC^a^, mean (SD)	Specificity	F1	AUC^a^, mean (SD)	Specificity	F1	AUC^a^, mean (SD)	Specificity	F1	AUC^a^, mean (SD)	Specificity	F1
**Boundary and domain-based method**													
	SVDD^b^	97.4 (2.9)	89.0 (3.4)	89.4 (7.1)	97.4 (1.8)	86.7 (4.4)	91.5 (10.9)	97.2 (2.6)	80.1 (5.5)	93.5 (3.4)	97.6 (1.7)	81.8 (5.3)	94.6 (6.0)
	IncSVDD^c^	97.1 (2.9)	87.7 (2.7)	89.5 (5.9)	97.2 (1.8)	86.4 (2.8)	93.6 (4.8)	97.0 (2.7)	76.2 (6.3)	93.2 (2.6)	97.4 (1.7)	79.0 (4.8)	*95.4 (1.9)* ^d^
	V-SVM^e^	98.1 (2.0)	85.5 (0.6)	*92.3 (1.3)*	98.9 (1.4)	89.8 (0.2)	*95.4 (1.6)*	98.7 (1.4)	86.4 (0.4)	*94.4 (2.0)*	99.0 (0.9)	89.2 (0.3)	*95.4 (2.1)*
	NN^f^	93.2 (7.8)	92.0 (2.4)	83.9 (12.0)	94.4 (2.5)	88.4 (3.4)	90.9 (5.3)	93.3 (2.8)	83.0 (3.7)	92.0 (4.2)	94.0 (2.8)	82.9 (3.6)	94.0 (4.0)
	MST^g^	96.1 (2.6)	94.4 (2.2)	72.9 (18.5)	97.3 (1.4)	94.2 (2.1)	86.1 (11.0)	96.1 (2.1)	93.5 (1.9)	90.2 (7.3)	97.0 (1.4)	93.6 (1.7)	92.6 (5.0)
**Density-based method**													
	Gaussian	98.4 (1.6)	91.2 (2.6)	89.6 (12.5)	99.3 (0.9)	92.3 (1.7)	95.7 (4.9)	98.8 (1.3)	88.1 (4.0)	95.9 (2.7)	99.2 (0.7)	89.8 (3.1)	*97.2 (1.8)*
	MOG^h^	97.5 (3.0)	91.7 (3.2)	87.8 (13.3)	98.9 (1.2)	90.9 (2.7)	94.0 (6.3)	98.2 (2.0)	85.4 (6.6)	94.2 (4.1)	98.5 (1.5)	88.0 (4.9)	96.0 (3.1)
	MCD^i^ Gaussian	98.5 (1.5)	89.9 (3.7)	*89.1 (11.8)*	99.5 (0.9)	92.2 (92.2)	*95.8 (4.5)*	98.9 (1.1)	87.9 (3.3)	*96.0 (2.5)*	99.2 (0.7)	90.4 (3.4)	*97.4 (1.7)*
	Parzen	96.4 (2.6)	97.8 (1.1)	59.9 (18.9)	98.0 (1.6)	97.7 (1.1)	79.5 (14.5)	97.2 (2.3)	96.4 (1.2)	85.1 (10)	98.1 (1.6)	96.7 (1.1)	88.6 (7.1)
	Naïve Parzen	96.4 (3.0)	87.5 (3.5)	85.1 (10.9)	98.7 (1.5)	89.2 (2.8)	92.8 (7.5)	96.0 (2.3)	90.8 (2.6)	95.0 (4.1)	98.2 (1.6)	90.0 (1.8)	96.2 (2.8)
	K-NN^j^	97.6 (2.9)	91.1 (1.6)	87.6 (13.6)	99.0 (1.4)	92.4 (2.4)	94.5 (6.6)	98.4 (1.4)	92.6 (1.4)	95.7 (4.8)	98.7 (1.1)	93.3 (1.3)	*97.3 (2.8)*
	LOF^k^	96.9 (2.9)	91.2 (1.6)	86.2 (13.0)	97.4 (1.8)	89.8 (4.8)	93.1 (4.9)	95.0 (3.0)	85.2 (4.6)	92.9 (4.8)	95.8 (1.7)	85.3 (4.7)	94.7 (3.2)
**Reconstruction-based method**													
	PCA^l^	97.4 (3.2)	78.2 (6.1)	82.5 (10.9)	94.8 (3.8)	77.6 (4.5)	90.9 (3.6)	92.6 (4.2)	72.4 (3.8)	92.5 (1.9)	93.4 (3.2)	71.1 (2.5)	93.9 (1.1)
	Auto-encoder	95.4 (5.3)	88.7 (9.5)	86.1 (13.1)	96.9 (3.2)	87.1 (9.9)	92.8 (6.4)	95.0 (5.3)	79.3 (14.5)	93.1 (4.8)	95.9 (4.3)	80.3 (14.4)	95.0 (3.6)
	SOM^m^	95.9 (2.9)	91.6 (2.6)	86.1 (14.4)	95.7 (1.7)	87.6 (4.1)	92.7 (5.7)	93.9 (3.5)	79.1 (10.9)	92.3 (4.5)	96.0 (2.5)	87.5 (7.0)	96.1 (3.2)
	K-means	97.1 (3.9)	89.7 (6.7)	*88.7 (12.1)*	98.6 (1.7)	91.1 (4.2)	*95.2 (4.4)*	98.5 (1.5)	92.3 (2.9)	*96.9 (3.3)*	98.9 (1.0)	93.9 (1.3)	*97.9 (2.2)*

^a^AUC: area under the receiver operating characteristic curve.

^b^SVDD: support vector data description.

^c^IncSVDD: incremental support vector data description.

^d^Italicized values indicates the top performing models.

^e^V-SVM: one-class support vector machine.

^f^NN: nearest neighbor.

^g^MST: minimum spanning tree.

^h^MOG: mixture of Gaussian.

^i^MCD: minimum covariance determinant.

^j^K-NN: K-nearest neighbor.

^k^LOF: local outlier factor.

^l^PCA: principal component analysis.

^m^SOM: self-organizing maps.

##### Unsupervised Methods

Two density-based unsupervised models were tested and evaluated on the same set of data as used in the one-class classifiers: LOF and COF. The average AUC, specificity, and F1-score were computed after 20 runs. The best performing thresholds for all the infection cases along with the optimal value of *k* (number of neighbors) are given in Table 8. As can be seen from the table, both the LOF and the COF achieved better performance on the smoothed data set as compared with its raw version. In all the infection cases, LOF performed better than COF. This is mainly because of the characteristics of the data sets, which fulfill the LOF spherical assumption of neighbor distribution. Considering the average F1-score across all the infection cases, LOF achieved 74.7% on the raw daily data, 91.1% on the smoothed daily data, and 72.7% on the hourly data, whereas COF achieved 71.9% on the raw daily data, 85.8% on the smoothed daily data, and 68.9% on the hourly data. However, compared with the one-class classifier, it suffers from performance degradation mainly because the data are not distributed uniformly, where some regions may contain high density and others might be sparse. However, the region of sparse density does not always signify anomalies (infection incidence). For example, an individual patient on certain days might prefer to take little insulin compared with most of the days and perform heavy physical activity to replace their insulin needs. This scenario could generate an outlier, a small ratio of insulin-to-carbohydrate, which will be considered and detected as outliers by unsupervised models. A detailed score plot of each model for the different infection cases can be found in Multimedia Appendix 3.

**Table 8:** Average area under the receiver operating characteristic curve, specificity, and F1-score for both with and without smoothed versions of the data. The parameters kd and kh represent the optimal number of nearest neighbors for the daily and hourly cases, respectively.

Frequencies, density-based methods														
	Pre-pro	Models (threshold)	1st case of infection (k_d_=30, k_h_=240)			2nd case of infection (k_d_=30, k_h_=240)			3rd case of infection (k_d_=30, k_h_=240)			4th case of infection (k_d_=30, k_h_=240)		
			AUC^a^	Specific	F1	AUC^a^	Specific	F1	AUC^a^	Specific	F1	AUC^a^	Specific	F1
**Daily**														
	Without filter	LOF^b^ (T_1_=2.4, T_2_=1.2, T_3_=1.45, T_4_=1.8)^c^	75.0	50.0	*85.6*	90.0	100	67.4	92.1	66.7	*70.1*	98.2	100	*75.8*
		COF^d^ (T_1_=1.4, T_2_=1.3, T_3_=1.4, T_4_=1.4)	82.1	66.7	72.6	97.4	100	*75.8*	75.2	66.7	67.6	96.7	100	71.8
	With filter	LOF^b^ (T_1_=1.7, T_2_=1.6, T_3_=1.95, T_4_=2.2)	99.0	100	*84.1*	99.2	100	*85.4*	100	100	*100*	99.9	100	94.7
		COF^d^ **(**T_1_**=**1.3**,** T_2_=1.3**,** T_3_=1.8, T_4_=1.8)	97.6	100	76.6	97.9	100	77.6	99.5	100	88.8	100	100	*100*
**Hourly**														
		LOF^b^ (T_1_=1.4, T_2_=1.3, T_3_=1.35, T_4_=1.5)	98.0	86.0	*74.6*	95.5	100	*70.2*	94.3	91.4	*75.0*	85.2	72.6	*71.1*
		COF^d^ (T_1_=1.2, T_2_=1.1, T_3_=, T_4_=1.1)	92.4	88.4	*74.6*	77.0	66.0	62.5	90.3	82.7	74.6	82.6	82.2	63.7

^a^AUC: area under the receiver operating characteristic curve.

^b^LOF: local outlier factor.

^c^T_k_: threshold for the kth month.

^d^COF: connectivity-based outlier factor.

##### Computational Time

**Figure 5:**
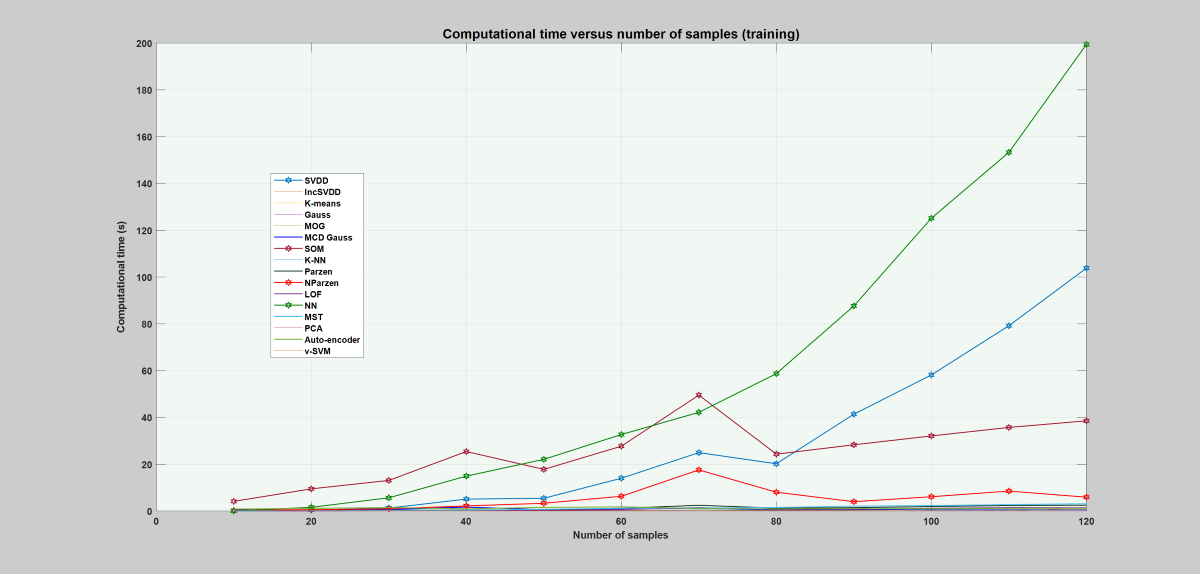
Plot of models’ average computational time for the training phase. The x-axis depicts the sample size, and each label stands for total sample size divided by 24. The y-axis depicts the computational time required by each model. Gauss: Gaussian; IncSVDD: incremental support vector data description; K-NN: K-nearest neighbor; LOF: local outlier factor; MCD: minimum covariance determinant; MOG: mixture of Gaussian; MST: minimum spanning tree; NN: nearest neighbor; NParzen: naïve Parzen; PCA: principal component analysis; SOM: self-organizing maps; SVDD: support vector data description; V-SVM: one-class support vector machine.

**Figure 6:**
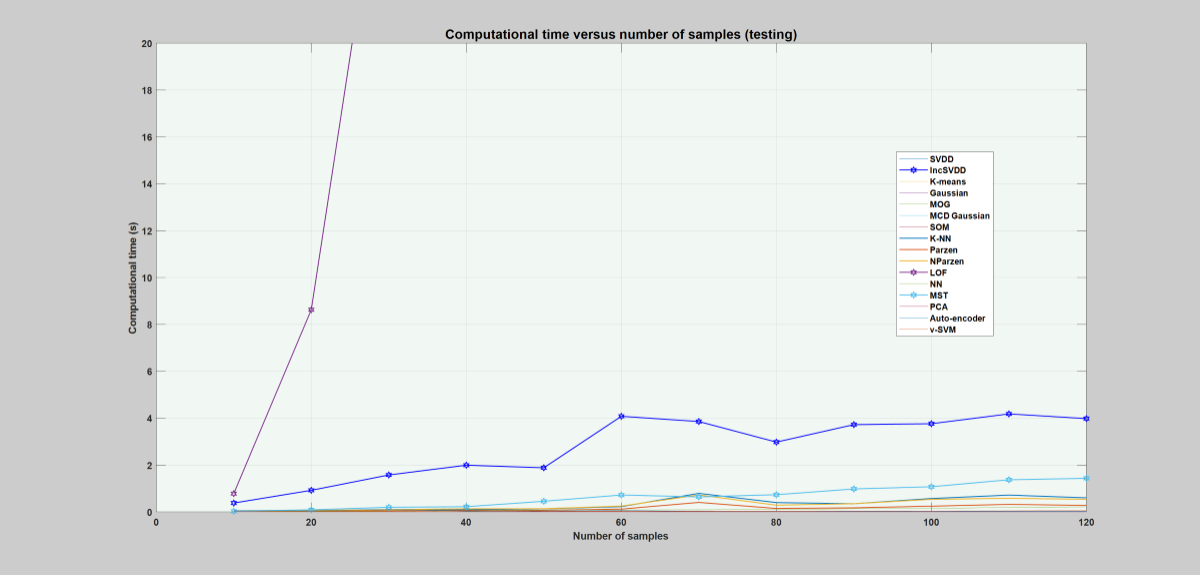
Plot of models’ average computational time for the testing phase. The x-axis depicts the sample size, and each label stands for total sample size divided by 24. The y-axis depicts the computational time required by each model. Gauss: Gaussian; IncSVDD: incremental support vector data description; K-NN: K-nearest neighbor; LOF: local outlier factor; MCD Gauss: Gaussian: SOM: self-organizing maps; MOG: mixture of Gaussian; MST: minimum spanning tree; NN: nearest neighbor; NParzen: naïve Parzen; PCA: principal component analysis; SVDD: support vector data description; V-SVM: one-class support vector machine.

Computational time is the amount of time a particular model needs to learn and execute a given task [12]. It can be regarded as one of the best performance indicators for real-time systems. For a real-time application, an optimal model is the one that achieves superior detection performance with small training and testing time**.** Depending on the application, sometimes models can be trained offline, which makes the training time less important [12]. In this regard, the computational times of all the models were estimated and compared with each other. The computational time was measured for different sample sizes of the training and testing data sets. The sample size of the training and testing data includes 240, 480, 720, 960, 1200, 1440, 1680, 1920, 2160, 2400, 2640, and 2880 sample objects (data points) each. The required computational time for both training and testing each model is depicted in Figures 5 and 6. The figures demonstrate a rough estimation of the computational time, where each model learns the data set and classifies the sample objects. During the training phase, NN, SVDD, and SOM took considerable time. For a training sample size of 2880 objects, NN requires 296 times, SVDD requires 206 times, and SOM requires 42 times the time taken by K-NN on the same sample size. Generally, as the number of sample objects increases, these models require much more time. However, K-means, Gaussian families, LOF, MST, K-NN, V-SVM, PCA, auto-encoder, and incSVDD took less time. These models took almost constant time even when the number of samples increased. During the testing phase, only the LOF took considerable time compared with the other models, as can be seen in Figure 6.

## Discussion

### Principal Findings

Anomaly or novelty detection problem has been widely used in various applications including machine fault and sensor failure detection, prevention of credit card or identity fraud, health and medical diagnostics and monitoring, cyber-intrusion detection, and others [1-3]. In applications related to health and medical diagnostics and monitoring, the anomaly detection problem has been used to detect and identify the abnormal health state of an individual, for example, detecting abnormal patterns of heartbeat recorded using an electrocardiogram [1,51-54]. The omnipresence of various physiological sensors has facilitated circumstances for individuals to easily self-record health-related events and data for the purpose of self-informatics and management [55]. Currently, people are generating huge amounts of data on a daily basis that can contribute to both individual and public health purposes [54]. To this end, people with diabetes are not an exception, generating rich data in both quality and quantity, which is expected to further improve with advances in diabetes technologies. These data can provide valuable information if processed with the right tools and methodology, and in this regard, particular instance includes detecting novel or anomalous data points for various purposes. The availability of labeled data constrains the choice of methods in the anomaly detection problem [3,9-11]. Supervised anomaly detection methods are impractical for applications such as detecting infection incidences in people with type 1 diabetes for a number of reasons [10,12]. Blood glucose dynamics are affected by various other factors apart from infection incidences [19,56,57], and characterization of infection-induced anomalies (abnormal class) from the normal class [13] is a challenging task because of the following reasons:

1. There are no well-defined boundaries regarding how different pathogens affect various key parameters of blood glucose dynamics, including blood glucose levels, insulin injections, carbohydrate ingestions, physical activity or exercise load, and others. This results in poor boundary demarcation between the normal and abnormal classes.
2. Class boundaries defined for a single pathogen might not work for the other pathogens because the effect of different pathogens on the blood glucose dynamics could be different.
3. It is expensive and time consuming to collect infection-related data to explore and characterize pathogen-specific class boundaries. This results in ill-defined class boundaries even for an infection related to a single pathogen.
4. The degree of effect of the same pathogens on the blood glucose dynamics could differ between different individuals because of the difference in individual immunity, which further complicates the characterization task.
5. Lack of sufficient sample size for both the abnormal and the normal classes results in poor training and testing data sample size or imbalanced class problems.

**Figure 7:**
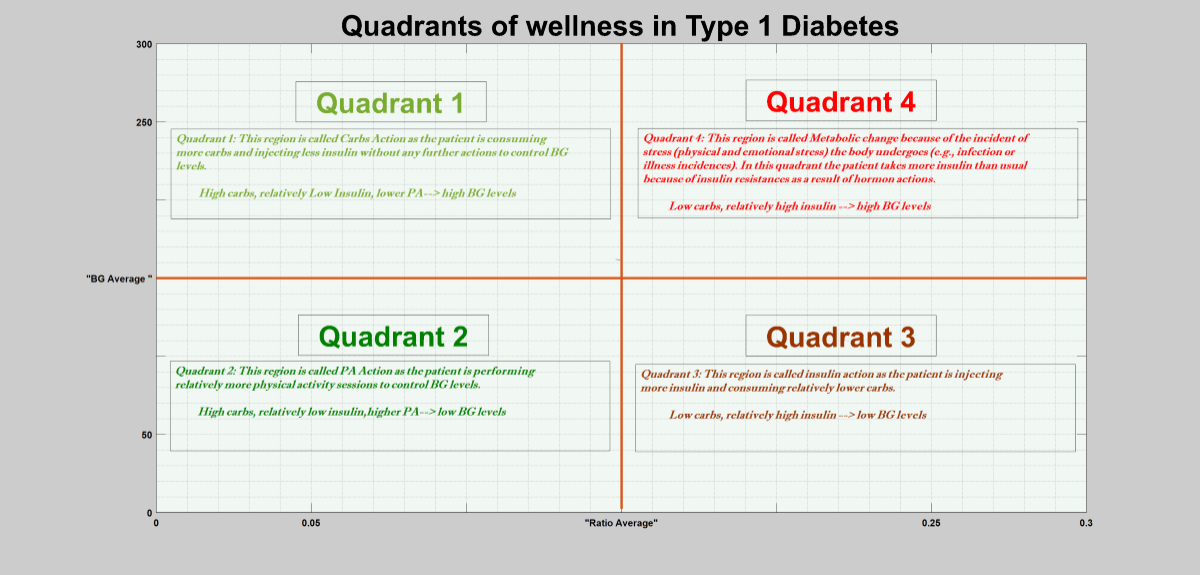
Quadrants of wellness in people with type 1 diabetes. The figure depicts the 4 possible scenarios of different parameters: carbohydrate action, insulin action, physical activity action, and abnormality because of metabolic change such as infection and stress. BG: blood glucose; PA: physical activity.

Given these challenges, the best possible approach is to identify methods that can learn from the normal health state of an individual and classify abnormalities relying on the boundaries learnt from the normal health state, which is a one-class classifier approach. This definitely reduces the challenge because it only requires the characterization of what is believed to be a normal health state. For instance, assume a health diagnostic and monitoring system that detects health changes in an individual by tracking the individual’s physiological parameters, where the current health status is examined based on set of parameters, and raises a notification alarm when the individual health deteriorates [12]. In such a system, it becomes feasible to rely on a method that can be trained using only the regular or normal day measurements (target days) so as to detect deviation from normality [12,14]. Another possible alternative approach is to identify a method that does not require any characterization and labeling of classes, which is unsupervised methods [7]. Accordingly, considering the previously mentioned challenges, one-class classifiers and unsupervised models were proposed for detecting infection incidence in people with type 1 diabetes. The objective was to develop a personalized health model that can automatically detect the incidence of infection in people with type 1 diabetes using blood glucose levels and insulin-to-carbohydrate ratio as input variables. The model is expected to detect any deviations from the norm as a result of infection incidences considering blood glucose level (hyperglycemia incidences) coupled with unusual changes in the insulin-to-carbohydrate ratio, that is, frequent insulin injections and unusual reduction in the amount of carbohydrate intake [19]. A personalized health model based on one-class classifiers and unsupervised methods was tested using blood glucose levels and the insulin-to-carbohydrate ratio as a bivariate input. The result demonstrated the potential of the proposed approach, which achieved excellent performance in describing the data set, that is, detecting infection days from the regular or normal days, and, in particular, the boundary and domain-based method performed better. Among the respective group, particular models such as V-SVM, K-NN, and K-means achieved excellent performance in all the sample sizes and infection cases. However, the unsupervised approaches suffer performance degradation compared with the one-class classifier mainly because of the atypical nature of the data, which are not distributed uniformly, where some regions may contain high density and others might be sparse (Multimedia Appendix 2). There are rare events (sparse region) of blood glucose dynamics that are a normal response; however, the unsupervised methods can still detect and flag false alarms including the following:

1. Carbohydrate action: a situation in which the ratio of insulin-to-carbohydrate is small and the blood glucose levels are high (hyperglycemia), *Carb Action-Quadrant 1* in Figure 7. This is a normal response to blood glucose dynamics as consumption of more carbohydrates and less insulin intake can derive blood glucose dynamics into the hyperglycemia region (high blood glucose levels) if there is no physical activity session. A typical example of this particular situation is holiday seasons, where people consume too many carbohydrates.
2. Physical activity action: despite a small ratio of insulin-to-carbohydrate, the blood glucose levels still drop to low levels (hypoglycemia), *PA Action-Quadrant 2* in Figure 7. Normally, a small ratio of insulin-to-carbohydrate signifies that the patient consumed more carbohydrates and injected less insulin, which normally derives the blood glucose dynamics into the hyperglycemia region. However, despite taking more carbohydrates and less insulin, a rigorous physical exercise can still derive the blood glucose dynamics into the hypoglycemia region. Therefore, this is a normal response of blood glucose dynamics as the action of physical activity or exercise can derive the patient into hypoglycemic regions even if the patient consumes more carbohydrates. For example, an individual patient on certain days might prefer to take little insulin as compared with most of the days and perform heavy physical activity to replace their insulin needs. This scenario could generate an outlier, a small ratio of insulin-to-carbohydrate, which will be considered and detected as anomalies by the unsupervised models. However, this could be mitigated by incorporating physical activity data as an input variable.
3. Insulin action: the ratio of insulin-to-carbohydrate is large, that is, high insulin intake and low carbohydrate consumption, and blood glucose levels are low (hypoglycemia), *Insulin Action-Quadrant 3* in Figure 7. This is a normal response to blood glucose dynamics as administration of high insulin with little carbohydrate consumption can derive the blood glucose dynamics into the hypoglycemic region.

The drawback of unsupervised methods is that they do not have any mechanism to handle rare events even if the events are normal. This is mainly because unsupervised methods define an anomaly on the basis of the entire data set. However, the one-class classifier can learn and handle such scenarios appropriately if presented during the training phase. This is mainly because one-class classifiers produce a reference description based on the available normal (target) data set, including the rare events. With regard to the one-class classifiers, the boundary and domain-based method achieved a better description of the data set compared with the density and reconstruction-based methods, mainly because of the ability of such models to handle the atypical nature of the data [12]. Detectability of the infection incidence is directly related to the extent and degree of the effect it induces on the blood glucose dynamics. The type of pathogen, individual’s immunity, and hormones involved could play a role in determining the degree of severity in this regard [19,24,58-62]. To this end, the results demonstrated that the models were capable of detecting all the infection incidences that can significantly alter the blood glucose dynamics, such as influenza. Moreover, infection incidence that had a moderate effect on the blood glucose dynamics, such as mild common cold without fever, was also detected. However, as expected, infection incidences that had almost little effect on the blood glucose dynamics, such as light common cold without fever, as reported by the individual patient, were not detected. Regarding the computational time, NN, SVDD, and SOM took considerable training time, which typically increased as the number of sample objects increased. Moreover, compared with the other models, only LOF and COF took considerable testing time.

### Comparative Analysis of the Methods

Selecting the proper model for implementation in a real-world setting requires considering different characteristics of the model. This includes typical model characteristics such as performance in limited training sample size, robustness to outliers in the training data, required training and testing time, and complexity of the model (in terms of the number of model parameters).

#### Performance and Sample Size

**Figure 8:**
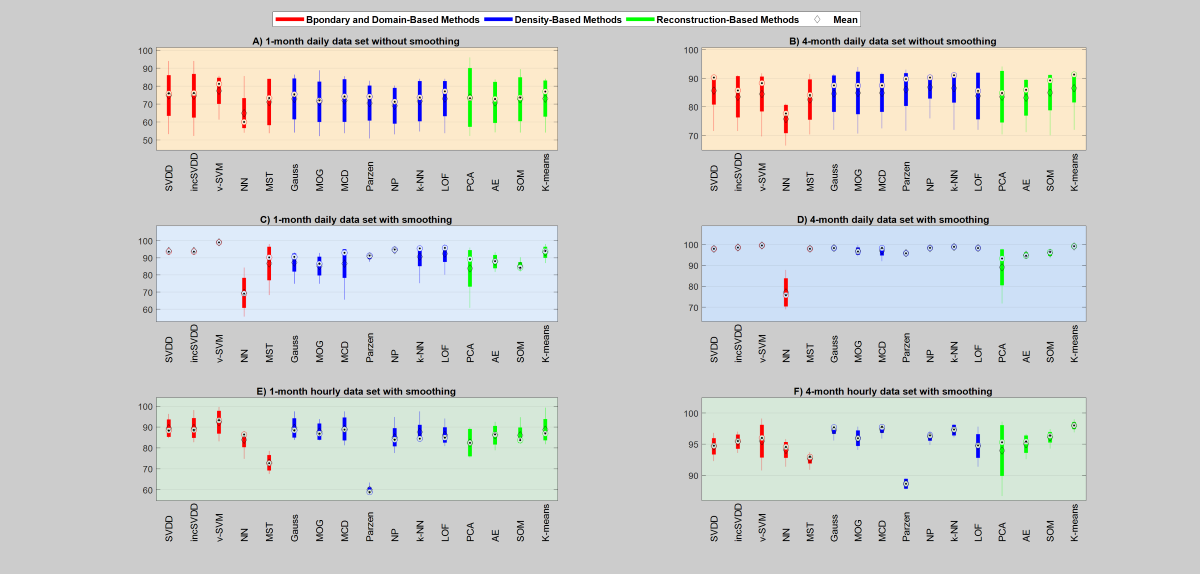
Average performance (F1-score) of each model across all the infection cases. AE: auto-encoder; Gauss: Gaussian; IncSVDD: incremental support vector data description; K-NN: K-nearest neighbor; LOF: local outlier factor; MCD: minimum covariance determinant; MOG: mixture of Gaussian; MST: minimum spanning tree; NN: nearest neighbor; NP: naïve Parzen; PCA: principal component analysis; SOM: self-organizing maps; SVDD: support vector data description; V-SVM: one-class support vector machine.

The sample size, N, is the number of sample objects used during the training phase and highly affects the generalization power of the model [12,13]. Models trained with small sample sizes often fail to produce satisfactory descriptions mainly associated with the presence of large variance in the sample objects [3,12,13,63]. To this end, the results indicate that most of the models fail to make good descriptions with a 1-month (30 objects) data set, mainly with the daily raw data set, as shown in Figure 8. The figure depicts the average performance of each model across all the infection cases over the 1- and 4-month sample sizes. Specifically, MST, Gaussian families, SOM, and auto-encoders require a considerable amount of training sample objects to better describe the data. There is some exception, for instance V-SVM, which produces a satisfactory description of the 1-month data sets in all the infection cases and data granularity. Models such as NN and PCA produced the worst description in most cases. As the number of training sample objects increased, all the models improved and produced a comparable description of the data. As a rule of thumb, for the daily scenario, a 3-month training sample (90 sample objects) produces a good description of the data, which can be considered for real-world applications. Moreover, if smoothing is considered, a 1-month sample size produces better description than the 4-month sample size without smoothing, as shown in Figure 8. However, for the hourly scenario, a 1-month training sample object produces a comparable description and anything more than this size will be enough.

#### Computational Time

For real-time applications, the time a model takes to learn and classify the sample object is essential in model selection. Table 9 depicts the rough estimation of average training and testing time required by different classifiers, both the one-class classifiers and the unsupervised models, based on 2880 training and testing sample objects each. Most of the models, as shown in Figures 5 and 6 and Table 9, require reasonable training and testing time, except NN, SVDD, and SOM, which took a considerably longer time. However, it is possible that in some cases models can be trained offline, which makes the training time less important. With regard to the testing time, most of the models executed the classification task in a reasonable time except COF and one class classifier version of LOF, which consume considerable time to classify the 2880 objects. The computational time in these particular models grows exponentially as the sample size increases, which makes them resource demanding in a big data setting.

**Table 9:** Rough estimation of average training and testing time required by the different classifiers.

Methods		Training time, mean (SD)		Testing time, mean (SD)	
**One-class classifiers**					
	SVDD^a^		105.2 (2.03)		0.008 (0.002)
	IncSVDD^b^		0.05 (0.16)		2.41 (0.83)
	K-means		0.0047 (0.0014)		0.0032 (0.0010)
	Gaussian		0.0055 (0.0032)		0.0032 (0.0012)
	MOG^c^		0.076 (0.018)		0.0036 (0.0011)
	MCD^d^ Gaussian		0.27 (0.075)		0.0034 (0.0015)
	SOM^e^		21.62 (5.91)		0.0033 (0.00087)
	K-NN^f^		0.51 (0.11)		0.52 (0.12)
	Parzen		2.02 (0.41)		0.21 (0.052)
	Naïve Parzen		4.02 (0.82)		0.40 (0.10)
	LOF^g^		1.15 (0.28)		1198.05 (323.07)
	NN^h^		151.34 (22.52)		0.18 (0.024)
	MST^i^		2.39 (0.31)		1.24 (0.19)
	PCA^j^		0.046 (0.20)		0.0031 (0.00086)
	Auto-encoder		0.65 (0.094)		0.017 (0.0034)
	V-SVM^k^		0.32 (0.024)		0.035 (0.0066)
**Unsupervised**					
	LOF^l^		N/A^m^		0.2 (0.0)
	COF^n^		N/A		82.8 (1.5)

^a^SVDD: support vector data description.

^b^IncSVDD: incremental support vector data description.

^c^MOG: mixture of Gaussian.

^d^MCD: minimum covariance determinant.

^e^SOM: self-organizing maps.

^f^K-NN: K-nearest neighbor.

^g^LOF: local outlier factor.

^h^NN: nearest neighbor.

^i^MST: minimum spanning tree.

^j^PCA: principal component analysis.

^k^V-SVM: one-class support vector machine.

^l^LOF: local outlier factor.

^m^N/A: not applicable.

^n^COF: connectivity-based outlier factor.

#### Robustness to Outliers in the Training Data Set

The presence of outliers in the training data set could significantly affect the model’s generalization ability. Outlier objects are samples that exhibit different characteristics compared with the rest of the objects in the data set [8,63]. For instance, an individual might forget a previous infection incident and could label these days as a regular or normal period during self-reporting, which could end up being used as target data sets for training. Another important example could be error recorded during data registration, that is, carbohydrate, blood glucose levels, and insulin registration. Such errors could occur during the manual registration of carbohydrates, associated with infusion set failures and other similar situations. In this scenario, an individual could record lower or higher values incorrectly affecting the input features, for example, ratio of insulin-to-carbohydrate and blood glucose levels, resulting in an outlier that could greatly affect the model’s generalization ability. In this type of situation, a model’s sensitivity to outliers in the training data is crucial to curb the influence of outliers on the accuracy of the description generated. To some extent, a user-specified empirical rejection rate is incorporated in the models to reduce the effect of outliers in the training data by rejecting the most dissimilar objects from the description generated. For example, a rejection rate of 1% on training data sets implies that 1% of outliers in the training data set are rejected. Nevertheless, the sensitivity of models to outliers in the training data sets differs greatly between models. Among the models, NN is regarded as the most sensitive model to outliers in the training data set [12]. The presence of outliers in the training data changes the shape of the description generated by the model, forcing a larger portion of the feature space to be accepted as the target class [10,12]. Furthermore, models that rely on an estimation of the covariance matrix, for example, Gaussian families, also suffer from the presence of outliers in the training data sets [12,36]. However, when equipped with regularization, Gaussian models can withstand such outliers. Local density estimators such as Parzen can withstand outliers, considering the fact that only the local density is affected [12]. Models that rely on prototype estimation, such as SOM and K-means, are highly affected by the presence of outliers in the training data set, which could force the estimated prototype to be placed near or at the nontarget data set [2,12,13]. Nevertheless, boundary and domain-based method such as SVDD and V-SVM and reconstruction-based method such as auto-encoders are more or less insensitive to outliers and can generate acceptable solutions [3,12,64].

#### Model Parameters and Associated Complexity

The parameters of a model can be either free or user defined. These two parameters, free and user defined, provide insight into how flexible the model is, how sensitive the model is to overtraining, and how easy the model is to configure (simplicity) [12,16]. Considering the number of these parameters, there exist large variations among the models. For instance, NN does not possess any free parameters; therefore, its performance completely relies on the training data set [12]. This constraint has limitations, mainly because training data that contain outliers could ruin the model’s performance [12,15,16]. A model that possess large number of free and user defined parameters is too flexible and complex [12]**.** Regarding the user-defined parameters, also known as hyper-parameters, a model equipped with small number of parameters and preferably with intuitive meaning are easy to configure. Setting up the user defined parameters incorrectly can degrade the model’s performance and selecting the proper values (optimization) becomes complex and vague as the number of model parameters become too large. One of the simplest models is Parzen density and NN, which do not require the user to specify any parameters [3,12,13]. Some models, such as support vector families, require the user to specify parameters that have intuitive meaning, for example, the ratio of training objects to be rejected by the description [12,65]. There are also models that are complex enough given that the user is expected to specify many parameters, which are not intuitive and require careful choice. Examples of such models include SOM and auto-encoders, where the user is expected to supply the number of neuron, hidden units, and learning rate [10,12,37,66].

### Practical Illustration and Area of Applications

For a real-world application, apart from the performance of the model, it is important to consider two important aspects of the data set, the time window of detection (data granularity) and the required sample size. The time window or data granularity, that is, hourly and daily, defines the frequency (continuity) of computation one needs to conduct throughout the day to screen the health status of the individual with type 1 diabetes. In an hourly time window, one is expected to carry out the computation at the end of each hour throughout the day. However, in the daily time window, one needs to carry out one aggregate computation at the end of the day. Decreasing the time window (increasing the granularity of the data) enhances early detections; however, at the coast of accuracy, for example, more unwanted features (noise) in the data. The results demonstrated that almost all the models produced fairly comparable detection performances in both time windows. Moreover, the required sample size determines the necessary amount of data an individual with type 1 diabetes needs to collect in advance before joining such an infection detection system. Models that could generalize well with small sample sizes could be preferred in a real-world application to enable more people to join the system with ease. Generally, the results demonstrated that the models require at least a sample size of 3-month data for the daily case and 2-month data for hourly case to perform better. Automating the detection of infection incidences among people with type 1 diabetes can deliver a means to provide personalized decision support and learning platforms for the individuals and, at the same time, can be used to detect infectious disease outbreaks on a large scale through spatio-temporal cluster detection [19,67,68]. Detailed descriptions of these instances are given below:

1. A personalized decision support system and learning platform relies on an individual’s self-recorded data to provide relevant information in relation to decision making to assist the individuals during crises [19,67,68]. Moreover, it can also provide a learning platform concerning the extent to which infection incidence affects the key parameters of the blood glucose dynamics. Information regarding what to expect at each stage of the course of infection could be very important to the individuals [19]. During infection incidences, various kinds of information could be vital for an individual to properly manage blood glucose levels, including time in range (blood glucose), to what extent is the evolution of blood glucose affected during the course of infection, to what extent does insulin sensitivity change, and how much does the insulin-to-carbohydrate ratio shift, that is, changes in insulin requirements for each gram of carbohydrate intake.
2. A population-based early outbreak detection system relies on self-recorded information from an individual with type 1 diabetes to detect individuals’ infection cases and, thereby, detect a group of infected individuals on a spatio-temporal basis. Such a system should collect individuals’ self-recoded data to a central server, analyze individuals’ data on a timely basis, identify and locate a cluster of people based on space and time, and notify the responsible bodies if there is an ongoing outbreak [19,67-71].

### Conclusions

Anomaly or novelty detection problem has been widely used in various applications including machine fault and sensor failure detection, prevention of credit card or identity fraud, health and medical diagnostics and monitoring, cyber-intrusion detection, and others. In this study, we demonstrated the applicability of one-class classifiers and unsupervised anomaly detection methods for the purpose of detecting infection incidences in people with type 1 diabetes. In general, the proposed methods produced excellent performance in describing the data set, and particularly the boundary and domain-based method performed better. In contrast to the specific models, V-SVM, K-NN, and K-means achieved better generalization in describing the data set in all infection cases. Detecting the incidence of infection in people with type 1 diabetes can provide an opportunity to devise tailored services, that is, personalized decision support and a learning platform for the individuals, and can simultaneously be used for detecting potential public health threats, that is, infectious disease outbreaks, on a large-scale basis through a spatio-temporal cluster detection. Generally, we foresee that the results presented could encourage researchers to further examine the presented features along with other additional features of self-recorded data, for example, various CGM features and physical activity data, on a large-scale basis.

## Appendix

Multimedia Appendix 1 Theoretical background of the methods.

Multimedia Appendix 2 Detailed description of the models input features.

Multimedia Appendix 3 Score plot of the models for each patient year.

Multimedia Appendix 4 Model evaluations – performance of the models for each patient year.

## Abbreviations

AUC: area under the receiver operating characteristic curve
COF: connectivity-based outlier factor
IncSVDD: incremental support vector data description
K-NN: K-nearest neighbor
LOF: local outlier factor
MCD: minimum covariance determinant
MOG: mixture of Gaussian
MST: minimum spanning tree
NN: nearest neighbor
PCA: principal component analysis
SOM: self-organizing maps
SVDD: support vector data description
ROC: receiver operating characteristic curve
V-SVM: one-class support vector machine

## References

[1] Dunning T, Friedman E, Loukides M (2014). Practical Machine Learning: A New Look at Anomaly Detection.

[2] Agrawal S, Agrawal J (2015). Survey on anomaly detection using data mining techniques. Procedia Comput Sci.

[3] Pimentel MA, Clifton DA, Clifton L, Tarassenko L (2014). A review of novelty detection. Sig Process.

[4] Cohen G, Hilario M, Sax H, Hugonnet S, Pellegrini C, Geissbuhler A (2004). An application of one-class support vector machine to nosocomial infection detection. Stud Health Technol Inform.

[5] Cohen G, Sax H, Geissbuhler A (2008). Novelty detection using one-class Parzen density estimator. An application to surveillance of nosocomial infections. Stud Health Technol Inform.

[6] Cohen G, Hilario M, Sax H, Hugonnet S, Geissbuhler A (2006). Learning from imbalanced data in surveillance of nosocomial infection. Artif Intell Med.

[7] Chandola V, Banerjee A, Kumar V (2009). Anomaly detection: a survey. ACM Comput Surv.

[8] Hawkins DM (1980). Identification of Outliers.

[9] Mehrotra KG, Mohan CK, Huang H, Subrahmanian VS (2017). Anomaly Detection Principles and Algorithms.

[10] Khan SS, Madden MG (2014). One-class classification: taxonomy of study and review of techniques. Knowl Eng Rev.

[11] Ding X, Li Y, Belatreche A, Maguire LP (2014). An experimental evaluation of novelty detection methods. Neurocomputing.

[12] Tax DM (2002). One-Class Classification: Concept Learning in the Absence of Counter-Examples.

[13] Irigoien I, Sierra B, Arenas C (2014). Towards application of one-class classification methods to medical data. ScientificWorldJournal.

[14] Japkowicz N (1999). Concept Learning in the Absence of Counterexamples: an Autoassociation-Based Approach to Classification. Semantic Scholar.

[15] Juszczak P, Tax DM, Pe¸kalska E, Duin RP (2009). Minimum spanning tree based one-class classifier. Neurocomputing.

[16] Mazhelis O (2006). One-class classifiers: a review and analysis of suitability in the context of mobile-masquerader detection. S Afr Comput J.

[17] Clark M, Ogden J (2004). What is diabetes?. Understanding Diabetes.

[18] Ogurtsova K, da Rocha FJ, Huang Y, Linnenkamp U, Guariguata L, Cho N, Cavan D, Shaw J, Makaroff L (2017). IDF diabetes atlas: global estimates for the prevalence of diabetes for 2015 and 2040. Diabetes Res Clin Pract.

[19] Woldaregay A, Årsand E, Albers D, Launonen I, Hartvigsen G (2020). Towards detecting infection incidences in people with type 1 diabetes using self-recorded data: a novel framework for a digital infectious disease detection mechanism. JMIR preprints.

[20] Marcovecchio ML, Chiarelli F (2012). The effects of acute and chronic stress on diabetes control. Sci Signal.

[21] Rayfield EJ, Ault MJ, Keusch GT, Brothers MJ, Nechemias C, Smith H (1982). Infection and diabetes: the case for glucose control. Am J Med.

[22] Botsis T, Lai AM, Hripcsak G, Palmas W, Starren JB, Hartvigsen G (2012). Proof of concept for the role of glycemic control in the early detection of infections in diabetics. Health Informatics J.

[23] Botsis T, Hejlesen O, Bellika JG, Hartvigsen G (2007). Blood Glucose Levels as an Indicator for the Early Detection of Infections In Type-1 Diabetics. Faculty & Staff Insider - University of Washington.

[24] Mizock BA (1995). Alterations in carbohydrate metabolism during stress: a review of the literature. Am J Med.

[25] Bosarge PL, Kerby JD (2013). Stress-induced hyperglycemia: is it harmful following trauma?. Adv Surg.

[26] Kajbaf F, Mojtahedzadeh M, Abdollahi M (2007). Mechanisms underlying stress-induced hyperglycemia in critically ill patients. Therapy.

[27] Tax DM, Duin RP (2004). Support vector data description. Mach Learn.

[28] Schölkopf B, Williamson RC, Smola AJ, Shawe-Taylor J, Platt JC (1999). Support Vector Method for Novelty Detection. NIPS Proceedings.

[29] Tax DM, Duin RP (1999). Support vector domain description. Pattern Recognit Lett.

[30] Ridder DD, Tax DM, Duin, RP (1998). An Experimental Comparison of One-Class Classification Methods. Proceedings of the 4th Annual Conference of the Advanced School for Computing and Imaging.

[31] Breunig MM, Kriegel H, Ng RT, Sander J (2000). LOF: identifying density-based local outliers. SIGMOD Rec.

[32] Tax D (2015). Software. DDTools.

[33] (2016). A Collection of Algorithms for Anomaly/Outlier Detection. Anomaly Detection Toolbox.

[34] Tang J, Chen Z, Fu AW, Cheung DW (2002). Enhancing effectiveness of outlier detections for low density patterns. Advances in Knowledge Discovery and Data Mining.

[35] Duin R, Juszczak P, Paclik P, Pekalska E, De Ridder D, Tax DM (2007). Software. Delft University of Technology.

[36] Goldstein M, Uchida S (2016). A comparative evaluation of unsupervised anomaly detection algorithms for multivariate data. PLoS One.

[37] Swersky L, Marques H, Sander J, Campello RJ, Zimek A (2016). On the Evaluation of Outlier Detection and One-Class Classification Methods. IEEE International Conference on Data Science and Advanced Analytics (DSAA).

[38] Rousseeuw PJ, Driessen KV (1999). A fast algorithm for the minimum covariance determinant estimator. Technometrics.

[39] Parzen E (1962). On estimation of a probability density function and mode. Ann Math Statist.

[40] Rasoulzadeh V, Erkus EC, Yogurt TA, Ulusoy I, Zergeroğlu SA (2016). A comparative stationarity analysis of EEG signals. Ann Oper Res.

[41] Azami H, Mohammadi K, Bozorgtabar B (2012). An improved signal segmentation using moving average and Savitzky-Golay filter. J Signal Inf Process.

[42] Kandanaarachchi S, Muñoz MA, Hyndman RJ, Smith-Miles K (2019). On normalization and algorithm selection for unsupervised outlier detection. Data Min Knowl Disc.

[43] Tax DM, Muller KA (2004). A Consistency-based Model Selection for One-Class Classification. Proceedings of the 17th International Conference on Pattern Recognition.

[44] Maldonado S, Montecinos C (2014). Robust classification of imbalanced data using one-class and two-class SVM-based multiclassifiers. Intell Data Anal.

[45] Tharwat A (2018). Classification assessment methods. Appl Comput Inf.

[46] Nguyen GH, Bouzerdoum A, Phung SL (2009). Learning pattern classification tasks with imbalanced data sets. Pattern Recog.

[47] Hajizadeh S, Li Z, Dollevoet RP, Tax DM, Fränti P, Brown G, Loog M, Escolano F, Pelillo M (2014). Evaluating classification performance with only positive and unlabeled samples. Structural, Syntactic, and Statistical Pattern Recognition: Joint IAPR International Workshop.

[48] Hajizadeh S, Núñez A, Tax DM (2016). Semi-supervised rail defect detection from imbalanced image data. IFAC-PapersOnLine.

[49] Bradley AP (1997). The use of the area under the ROC curve in the evaluation of machine learning algorithms. Pattern Recog.

[50] McNamara LA, Martin S, Long SS, Prober CG, Fischer M (2018). Principles of epidemiology and public health. Principles and Practice of Pediatric Infectious Diseases. Fifth Edition.

[51] Zhu Y (2011). Automatic detection of anomalies in blood glucose using a machine learning approach. J Commun Netw.

[52] Zhu Y (2010). Automatic Detection of Anomalies in Blood Glucose Using a Machine Learning Approach. International Conference on Information Reuse & Integration.

[53] Spinosa EJ, Carvalho AC (2005). Support vector machines for novel class detection in Bioinformatics. Genet Mol Res.

[54] Lotze TH (2009). Anomaly Detection in Time Series: Theoretical and Practical Improvements for Disease Outbreak Detection. Digital Repository at the University of Maryland.

[55] Tsui F, Espino JU, Dato VM, Gesteland PH, Hutman J, Wagner MM (2003). Technical description of RODS: a real-time public health surveillance system. J Am Med Inform Assoc.

[56] Woldaregay AZ, Årsand E, Botsis T, Albers D, Mamykina L, Hartvigsen G (2019). Data-driven blood glucose pattern classification and anomalies detection: machine-learning applications in type 1 diabetes. J Med Internet Res.

[57] Oviedo S, Vehí J, Calm R, Armengol J (2017). A review of personalized blood glucose prediction strategies for T1DM patients. Int J Numer Method Biomed Eng.

[58] Yki-Järvinen H, Sammalkorpi K, Koivisto VA, Nikkilä EA (1989). Severity, duration, and mechanisms of insulin resistance during acute infections. J Clin Endocrinol Metab.

[59] Rayfield EJ, Curnow RT, George DT, Beisel WR (1973). Impaired carbohydrate metabolism during a mild viral illness. N Engl J Med.

[60] McGuinness OP (2005). Defective glucose homeostasis during infection. Annu Rev Nutr.

[61] Brealey D, Singer M (2009). Hyperglycemia in critical illness: a review. J Diabetes Sci Technol.

[62] Mizock BA (2001). Alterations in fuel metabolism in critical illness: hyperglycaemia. Best Pract Res Clin Endocrinol Metab.

[63] Tax DM, Duin RP (2005). Characterizing One-Class Datasets. CiteSeerX.

[64] Wang D, Yeung DS, Tsang EC (2006). Structured one-class classification. IEEE Trans Syst Man Cybern B Cybern.

[65] Janssens JH (2013). Outlier Selection and One-Class Classification.

[66] Wang B, Mao Z (2017). One-class classifiers ensemble based anomaly detection scheme for process control systems. T I Meas Control.

[67] Samerski S (2018). Individuals on alert: digital epidemiology and the individualization of surveillance. Life Sci Soc Policy.

[68] Radin JM, Wineinger NE, Topol EJ, Steinhubl SR (2020). Harnessing wearable device data to improve state-level real-time surveillance of influenza-like illness in the USA: a population-based study. Lancet Digit Health.

[69] Woldaregay A, Årsand E, Giordanengo A, Albers D, Mamykina L, Botsis T, Hartvigsen G (2017). EDMON-A Wireless Communication Platform for a Real-time Infectious Disease Outbreak Detection System Using Self-recorded Data From People With Type 1 Diabetes. The 15th Scandinavian Conference on Health Informatics.

[70] Coucheron S, Woldaregay AZ, Årsand E, Botsis T, Hartvigsen G (2019). EDMON - A System Architecture for Real-Time Infection Monitoring and Outbreak Detection Based on Self-Recorded Data from People with Type 1 Diabetes: System Design and Prototype Implementation. The 17th Scandinavian Conference on Health Informatics.

[71] Yeng PK, Woldaregay AZ, Solvoll T, Hartvigsen G (2020). Cluster detection mechanisms for syndromic surveillance systems: systematic review and framework development. JMIR Public Health Surveill.

