# Supplementary material for: A Novel Approach for Continuous Health Status Monitoring and Automatic Detection of Infection Incidences in People With Type 1 Diabetes Using Machine Learning Algorithms (Part 2): A Personalized Digital Infectious Disease Detection Mechanism
Source: J Med Internet Res. 2020 Aug 12;22(8):e18912. doi: 10.2196/18912 (PMC7450372; doi:10.2196/18912)
Supplement: Multimedia Appendix 1 [file jmir_v22i8e18912_app1.docx]

# **Appendix 1: Theoretical Background of the Methods**

# Background

- - 1. Notion of Object

An object is described with a feature vector encompassing the number of parameters under consideration. For example, an object *k* can define a specific event of an individual BG dynamics at a specified time index *k*, and is represented by a feature vector $\mathcal{X}_{k}=\left( \mathcal{x}_{1,1}, \mathcal{x}_{1,2} \right)$, where $\mathcal{x}_{1,1}$ represent the insulin to carb ratio at the time index *k* and $\mathcal{x}_{1,2}$ represent the average BG level in the specified time bin around *k*.

- - 1. One-class Classifier

One-class classification problem can be regarded as a special type of two-class classification problem incorporating a target and non-target class [7, 9]. The target class is where a model is trained on, and is expected to incorporate well sampled representative object of the target that reflect the region of the data in the feature space. The non-target (outlier) class is sparsely represented and sometimes can be totally absent. The task of one-classification is governed mainly by distinct two elements; function (model) and threshold. Function (model) measures the resemblance of an object $\mathcal{x}$depending on the distance $\mathcal{d(x)}$ or probability $\mathcal{P(x)}$ to the target class described by the training dataset $\mathcal{X}_{\mathrm{tr}}$. A threshold ($\beta)$ is used to decide the belongingness of a test object $\mathcal{x}$ to either of the classes, i.e. non-target or the target class, depending on the definition of the function’s internal structure. For example, test objects are rejected when computed distance by the function (model) is greater than some specified threshold $\beta$,

$\boldsymbol{\psi}\left( \mathcal{x} \right)=\left\{ \begin{aligned} target I(\mathcal{d}\left( \mathcal{x} \right)\leq\beta) \\ non-target I(\mathcal{d}\left( \mathcal{x} \right)>\beta) \end{aligned} \right.$ (1)

Or when computed resemblance by the function (model) is less than some specified threshold $\beta$,

$\boldsymbol{\psi}\left( \mathcal{x} \right)=\left\{ \begin{aligned} target I(\mathcal{P}\left( \mathcal{x} \right)\geq\beta) \\ non-target I(\mathcal{P}\left( \mathcal{x} \right)<\beta) \end{aligned} \right.$ (2)

Where $\psi\left( \mathcal{x} \right)$ is the class label and $I$ is an indicator function.

Depending on the type of internal function (model) used, one-class classifier can be broadly categorized into three main groups; boundary and domain-based, density-based, and reconstruction-based method [5, 7, 10-12]. The main difference between these methods is the way they define of the function (model), and minimization approaches, thereby achieving different generalization, bias and overfitting as they consider different data characteristics [7].

- - - 1. *Boundary and Domain-Based Method*

Boundary-based method estimate a boundary, e.g. hyperplane/hypersphere, around majority of the training (target) dataset, where a predefined small percentage (fraction) of the target data are allowed to lie outside the specified boundary [11, 12]. A test object is regarded as outlier if it falls outside of the defined boundary. The resemblance of the test object is determined by computing the distance from the test object to the boundary estimated around the training objects. The distance computation is conducted by taking into account both I) the inter-distance between the new object and the training datasets and II) intra-distance between the objects in the training datasets. Different boundary and domain-based method exist including support vector data description (SVDD), one-class support vector machine (v-SVM), incremental support vector machine, nearest neighbors (NN), and minimum spanning tree (MST) [7, 10, 11].

**Support Vector, and Incremental Support Vector Data Description Vs. One-class Support Vector Classifier:** These methods describe the target class by fitting the training (target) dataset into either a hypersphere or a hyperplane respectively [7, 12, 13]. SVDD defines a hypersphere that encompass the entire target dataset with a volume as minimum as possible [14, 15]. SVDD carries out the minimization task through quadratic programming problem [9]. The incremental version is applicable to a problem that involves online and sequential data [16]. During the training phase, the parameter $\alpha_{i}$ are estimated by minimizing

$\boldsymbol{L}=\boldsymbol{\varepsilon}_{\boldsymbol{SVDD}}=\sum_{i} \alpha_{i}\left( \boldsymbol{x}_{i}\boldsymbol{.}\boldsymbol{x}_{j} \right)-\sum_{i,j} \alpha_{i}\alpha_{j}\left( \boldsymbol{x}_{i}\boldsymbol{.}\boldsymbol{x}_{j} \right)$ (3)

Subject to the constraints $\sum_{i} \alpha_{i}=1$ and $0\leq\alpha_{i}\mathbb{\leq c}$, where $\mathbb{c}$ specifies the number of vectors that will not be covered by the description. The minimization is solved via quadratic programing problem by relying on kernel function, e.g. Gaussian, to replace the inner product so as to transform the vectors into a higher dimensional feature space for a more accurate description. A new test object $x$ is evaluated by computing its distance to the hypersphere’s center and comparing against the hypersphere’s radius:

$\boldsymbol{\psi(x)}=\left\{ \begin{aligned} target, if \left\| x-а \right\|^{2}\leq\mathbb{R}^{2} \\ non-target, otherwise \end{aligned} \right.$ (4)

Where, $а$ is the hypersphere’s center, and is computed as $\sum_{i} \alpha_{i}x_{i}$. The hypersphere’s radius is calculated as:

$\mathbb{R=}\left( x_{k}.x_{k} \right)-2\sum_{i} \alpha_{i}\left( x_{i}.x_{k} \right)+\sum_{i,j} \alpha_{i}\alpha_{j}(x_{i}.x_{j})$ (5)

Where $x_{k}$ are the vectors, which have $\alpha_{i}\mathbb{<C}$.

The one-class support vector machine, i.e. v-SVM, considers a hyperplane that separate the target datasets from the origin with a maximum margin [13]. The following minimization problem is carried out to reach at a solution for the parameter $\alpha_{i}$:

$\begin{matrix} min \\ \alpha_{i,j} \end{matrix} \frac{1}{2} \sum_{i,j} \alpha_{i}\alpha_{j}K(x_{i},x_{j})$ (6)

Subject to $0\leq\alpha_{i}\leq\frac{1}{N\nu}, \sum_{i} \alpha_{i}=1,$

Where $\nu$ is similar to $\mathbb{C}$ in SVDD, and plays the role of regularization term. A new test object $x$ is evaluated by calculating the distance from the test object to the origin as follows:

$\boldsymbol{\psi(x)}=\left\{ \begin{aligned} target, if \sum_{i} \alpha_{i}K\left( x_{i},x \right)-\zeta\geq0, \\ non-target, otherwise \end{aligned} \right.$ (7)

$\zeta$ is computed as $\sum_{j} \alpha_{j}\left( x_{j},x_{i} \right)$, where $x_{i}$ is an object vector, for which $\alpha_{i}$ is not at the lower or upper bound.

**Nearest-Nearest Neighbor Data Description:** Uses the distances to the first nearest neighbor to approximate the local density of the target class [7, 17, 18]. A new test object $x_{i}$ is evaluated by measuring the distance to the first nearest neighbor, NN(x), in the target dataset. The estimated distance is then normalized by its nearest neighbor distance as given below:

$\boldsymbol{\rho}_{\boldsymbol{NN}}\left( x \right)=\frac{\left\| x-{NN}^{tr}(x) \right\|}{\left\| {NN}^{tr}\left( x \right)-{NN}^{tr}({NN}^{tr}(x) \right\|}$ (8)

Where, $\mathrm{NN}^{tr}\left( x \right)$ is the nearest neighbor of object $x.$ The classifier is defined based on a threshold $\beta$ as:

$\psi\left( x \right)=\left\{ \begin{aligned} \\ target if \boldsymbol{\rho}_{\boldsymbol{NN}}\left( x \right)\leq\beta\\ non-target if \boldsymbol{\rho}_{\boldsymbol{NN}}\left( x \right)>\beta\end{aligned} \right.$ (9)

**Minimum Spanning Tree Data Description:** Exploits the structure of minimum spanning tree to describe the target class [10]. A new test object is evaluated by calculating the distance from the object to the closest edge of the tree [9, 10]. The classifier is defined based on a specified threshold as given in equation 9.

- - - 1. *Density-Based Method*

Density-based method estimate the probability density distribution of the target object, where a test object that lies in the high density region is regarded as normal and anomaly if it lies in a low density region [6, 11]. Different variants of density-based methods exist such as Gaussian, minimum covariance Gaussian, mixture of Gaussian, Parzen, Naïve Parzen, local outlier factor, and k-nearest neighbor [7].

**Gaussian, Minimum Covariance Gaussian, and Mixture of Gaussian Data Description:** Gaussian data descriptions describe the target data by assuming that the data is either normally distributed or mixture of a number of normal distributions [6, 7, 9, 12]. Gaussian and MCD Gaussian data description models uses Mahanalobis distance estimate as resemblance measure instead of density estimate [9]. Normal Gaussian data description defines the Mahanalobis distance from a new test object $x$ to the training set $X$ based on the mean and covariance matrix of the training set [6, 9]:

$\boldsymbol{\rho}_{\boldsymbol{Maha}}\left( x \right)=\left( x-\mu^{tr} \right)^{T}\boldsymbol{\Sigma}^{\boldsymbol{-1}}(x-\mu^{tr})$*,*

where $\mu^{tr}$ is training sample mean

$\boldsymbol{\Sigma}=\frac{1}{\left| X \right|}\sum_{x^{k}\epsilon X} (x^{k}-\mu^{tr})(x^{k}-\mu^{tr})$*,* (10)

where $\left| X \right|$ is the number of objects in the training dataset.

The classifier is defined as:

$\boldsymbol{\psi(x)}=\left\{ \begin{aligned} \\ target if \rho_{Maha}\left( x \right)\leq\beta\\ non-target if \rho_{Maha}\left( x \right)>\beta\end{aligned} \right.$ (11)

The threshold $\beta$ is estimated by taking the user specified target error into consideration.

Minimum Covariance Gaussian data description is similar to Gaussian data description, except that the mean and covariance matrix is estimated using only a fraction of the target datasets that minimize the determinant of the covariance matrix [9, 19]. Mixture of Gaussian data description model define the target class using a linear combination of $k$ Gaussian [9, 11, 20]. A new test object $x$ is evaluated as follows:

$\boldsymbol{\rho}_{\boldsymbol{MOG}}\left( x \right)=\sum_{i=1}^{K} \boldsymbol{P}_{\boldsymbol{i}}exp(-({x-\mu_{i})}^{T})\boldsymbol{\Sigma}_{\boldsymbol{i}}^{\boldsymbol{-1}}(x-\mu_{i}))$ (12)

$P_{i}$ and $\Sigma_{i}$ are optimized using expectation minimization (EM) algorithm. The classifier is defined based on a specified threshold β as:

$\boldsymbol{\psi(x)}=\left\{ \begin{aligned} \\ target if \rho_{MOG}\left( x \right)\geq\beta\\ non-target if \rho_{MOG}\left( x \right)<\beta\end{aligned} \right.$ (13)

**Parzen and Naïve Parzen Data Description:** These models are non-parametric density estimators**,** which don’t take into account any assumption about the underlying data distribution [7, 11]. The density is directly estimated from the training datasets using a mixture of kernels, most often a Gaussian kernel, centered on each individual training dataset, with diagonal covariance matrix $\boldsymbol{\Sigma}_{\boldsymbol{i}}\boldsymbol{=hI}$**.** The smoothing parameter $h$ characterizes the density estimate, where large values results in overestimate and small values results in noisy estimation. The optimal value of the smoothing parameter is computed based on the maximum likelihood on the training data using leave-one-out approach [6, 7, 12]. A new test object is evaluated as follows:

$\boldsymbol{\rho}_{\boldsymbol{Parzen}}\left( \boldsymbol{x} \right)=\sum_{i=1}^{N} exp(-({x-x_{i})}^{T}\boldsymbol{h}^{\boldsymbol{-2}}(x-x_{i}))$ (14)

The classifier is defined as given in equation 13.

**K-Nearest Neighbor Data Description:** KNN describes the target class by approximating the local density of the training (target) datasets [12]. The distance to the dataset can be computed based on the distance to the k^th^ nearest neighbor, distance to the average of the k-nn’s, or average squared distance to the k-nn’s [9]. For example, considering the distance to the k^th^ nearest neighbor, an object is evaluated as a function of the score, which is the ratio of the distance from the object to its k^th^ nearest neighbors and the distance between the k^th^ nearest neighbor and its k^th^ nearest neighbors [12].

$\boldsymbol{\rho}_{\boldsymbol{Knn}}\left( \boldsymbol{x}_{\boldsymbol{i}}\boldsymbol{,k} \right)=\frac{d(x_{i}, {NN}_{k}\left( x_{i} \right))}{d( {NN}_{k}\left( x_{i} \right),{NN}_{k}\left( {NN}_{k}\left( x_{i} \right) \right))}$ (15)

The classifier is defined based on a specified threshold as given in equation 9.

**Local Outlier Factor Data Description:** Like KNN, LOF considers the local density of an object to its respective neighbors, however, the distance is replaced by reachability distance [21, 22].

$\boldsymbol{reach-dist}_{k}\left( x_{i}⟵x_{j} \right)=max\left\{ d\left( x_{j},{NN}_{k}\left( x_{j} \right) \right),d(x_{i},x_{j}) \right\}$ (16)

For an object $x_{i}$, the local reachability density is computed by taking the inverse average reachability distance from the set of $x_{i}^{'}$s neighbors, which are located within the k-nearest neighbor distance around $x_{i}:$

$\boldsymbol{lrd}_{\boldsymbol{k}}=\frac{1}{\frac{\sum_{x_{j}\in kNN(x_{i})} \boldsymbol{reach-dist}_{k}(x_{i}⟵x_{j})}{\left| kNN(x_{i}) \right|}}$ (17)

For an object $x_{i}$, the degree of outlierness score, called LOF score, is evaluated by comparing its reachability density ($lrd$) with its neighbors:

$\boldsymbol{LOF}_{\boldsymbol{k}}\left( \boldsymbol{x}_{\boldsymbol{i}} \right)= \frac{\sum_{x_{j}\in kNN(x_{i})} \frac{\boldsymbol{lrd}_{\boldsymbol{k}}(x_{i})}{\boldsymbol{lrd}_{\boldsymbol{k}}(x_{j})}}{\left| kNN(x_{i}) \right|}$ (18)

The classifier is defined based on a specified threshold as given in equation 9.

- - - 1. *Reconstruction-Based Method*

Reconstruction-based method make assumptions about the underlying data characteristics, which involves modelling of the data generating process by estimating the parameters during the training phase using the target objects [6, 17]. It is characterized by a set of prototypes/subspaces with minimal reconstruction errors. A test object is determined as either normal or anomaly based on the reconstruction error, which indicates how the test object fits to the model. Normal test objects usually generate minimum reconstruction error (closer fit) and anomalies generate high reconstruction errors. Reconstruction-based method includes different models; principal component analysis (PCA), self-organizing map (SOM), auto-encoder, and K-means, which mainly differ in their prototype/subspace definition, optimization principle, and the way the reconstruction error is used [7].

**Principal Component Analysis Data Description (PCA):** Computes the internal variance and external covariance structures of the target data in terms of set of principal components, which are a linear combinations of the original variables, to describe the data on a linear subspace [7, 9, 12]. The eigenvectors of the data covariance matrix $\boldsymbol{\Sigma}$ are used to define the corresponding subspace. Different PCA optimization techniques exist and yet eigenvalue decomposition is the simplest procedure to compute the eigenvectors of the target covariance matrix, $\boldsymbol{\Sigma}$ [7]. The number of basis vectors are computed depending on the fraction of variance the user intends to retain in the description. The projection is carried out as:

$x_{projected}\mathbb{=W(}{\mathbb{W}^{\mathbb{T}}\mathbb{W)}}^{-1}\mathbb{W}^{\mathbb{T}}x$***,*** (19)

where $\mathbf{x}_{\boldsymbol{projected}}$, and $\boldsymbol{x}$ are the new projected data and the original data respectively. Whereas $\mathbb{W}$ is a $\boldsymbol{d x k}$ matrix containing $\boldsymbol{k}$ eigenvectors, and $\boldsymbol{d}$ represent the original feature space dimensionality. A new test object is evaluated based on the reconstruction error, which is the difference between the original test object $\boldsymbol{x}$ and its projection onto the space, $\mathbf{x}_{\boldsymbol{projected}}$ [7]**.**

$\varepsilon_{reconstruction}=\left\| x-x_{projected} \right\|^{2}$ (20)

The classifier is defined based on a threshold $\beta$ as:

$\psi(x)=\left\{ \begin{aligned} \\ target if \varepsilon_{reconstruction}\leq\beta\\ non-target if \varepsilon_{reconstruction}>\beta\end{aligned} \right.$ (21)

**Auto-Encoder Data Description:** Neural network contains of a series of interconnected neurons at one or several layers, where training updates the weights connecting each respective neuron [12]. Neural network is capable of learning a complex functional mapping between the input and output features. Auto-encoder is a special type of neural network that learns the internal structure of the data to reconstruct the input features at the output [12]. Therefore, in one-class classification, the difference between the input and output features is taken into consideration to characterized the target class [7]. The reconstruction error is computed as:

$\varepsilon_{reconstruction}=\left\| x-f_{auto}(x) \right\|^{2}$ (22)

The classifier is similar to equation 21.

**K-Means and Self-Organizing Maps (SOM) Data Description:** Both are type of clustering methods, which relies on the assumption that the data can be clustered and described by a set of prototypes or codebook vectors $\boldsymbol{\mu}_{\mathbf{i}}$ [6, 7, 12]. Often the nearest prototypes, measured in terms of Euclidian distance, are used to represent the target object. K-means data description describes the training (target) data by k number of clusters, where the average distance to the cluster center is minimized [6, 7]. The standard k-means clustering procedure is used to place the center of the clusters ($\mu_{i})$ as follows:

$\varepsilon_{im}=\sum_{k} ({min}_{i}\left\| x_{k}-\mu_{i} \right\|^{2})$ (23)

Self-organizing map (SOM) is an unsupervised clustering method, where objects in the feature space are mapped into a space while retaining their distance and neighborhood relationships [7]. SOM performs a competitive learning so as to locate the position of the prototype vectors [12]. An update is carried out not only on the nearest prototype but also prototypes in the neighborhoods of the nearest prototype, which is specified by a predefined topology. However, the magnitude of update decreases as the distance increases and distant porotypes get smaller updates. A new test object is evaluated based on a reconstruction error, which is the difference between the test object and its closest cluster center (neuron) in either the K-means or SOM [7, 12]:

$\varepsilon_{reconstruction}={min}_{i}\left\| x-\mu_{i} \right\|^{2}$ (24)

The classifier is similar to equation 21.

- - 1. Unsupervised approach

Unsupervised approaches take unlabeled datasets as input and determine whether each objects in the datasets are normal or abnormal with respect to the entire dataset [21, 23]. There exist variety of unsupervised approaches in literatures, which can be categorized into statistical, nearest-neighbor based techniques, and cluster based approaches [21, 23]. In this paper, we have tested two nearest-neighbor based techniques (local density based methods), local outlier factor (LOF) and connectivity based outlier factor (COF), which compare the density of an object with its neighbors than the entire dataset [24]. The connectivity-based outlier factor and local outlier factor only differ in the way the density is estimated for a given object [23, 25]. LOF exploits the Euclidian distance measure to select the k-nearest neighbors, which is valid only if the data is distributed spherically around the object. This method often fails in some condition, for example, when objects in the dataset have a direct linear correlation. In this regard, COF improves this drawback by computing the local density of the neighborhood based on shortest-path approach, also known as chaining distance, which is the minimum of the sum of all distances connecting all k-neighbors and the object [23, 25].

# Reference

1. Hawkins, D.M., *Introduction*, in *Identification of Outliers*. 1980, Springer Netherlands: Dordrecht. p. 1-12.

2. Chandola, V., Banerjee, A., and Kumar, V., *Anomaly detection: A survey.* ACM computing surveys (CSUR), 2009. **41**(3): p. 15.

3. Dunning, T. and Friedman, E., *Practical machine learning: a new look at anomaly detection*. 2014: " O'Reilly Media, Inc.".

4. Mehrotra, K.G., Mohan, C.K., and Huang, H., *Anomaly detection principles and algorithms*. 2017: Springer.

5. Khan, S.S. and Madden, M.G., *One-class classification: taxonomy of study and review of techniques.* The Knowledge Engineering Review, 2014. **29**(3): p. 345-374.

6. Ding, X., Li, Y., Belatreche, A., and Maguire, L.P., *An experimental evaluation of novelty detection methods.* Neurocomputing, 2014. **135**: p. 313-327.

7. Tax, D.M.J., *One-class classification: Concept learning in the absence of counter-examples*, in *Technische Universiteit Delft*. 2002.

8. Japkowicz, N., *Concept-Learning in the Absence of Counter-Examples:An Autoassociation-Based Approach to Classification*, in *Computer Science*. 1999, Graduate School-New Brunswick Rutgers, The State University of New Jersey: New Brunswick, New Jersey.

9. Tax, D.M.J., *DDTools, the data description toolbox for MATLAB, version 2.1. 2.* Delft University of Technology, Delft, Netherlands, 2015.

10. Juszczak, P., Tax, D.M.J., Pe¸kalska, E., and Duin, R.P.W., *Minimum spanning tree based one-class classifier.* Neurocomputing, 2009. **72**(7): p. 1859-1869.

11. Irigoien, I., Sierra, B., and Arenas, C., *Towards application of one-class classification methods to medical data.* ScientificWorldJournal, 2014. **2014**: p. 730712.

12. Mazhelis, O., *One-class classifiers: a review and analysis of suitability in the context of mobile-masquerader detection.* South African Computer Journal, 2006. **2006**(36): p. 29-48.

13. Schölkopf, B., Williamson, R.C., Smola, A.J., Shawe-Taylor, J., and Platt, J.C., *Support Vector Method for Novelty Detection*. 1999. p. 582-588.

14. Tax, D.M.J. and Duin, R.P.W., *Support vector domain description.* Pattern Recognition Letters, 1999. **20**(11): p. 1191-1199.

15. Tax, D.M.J. and Duin, R.P.W., *Support Vector Data Description.* Machine Learning, 2004. **54**(1): p. 45-66.

16. Tax, D.M.J. and Laskov, P. *Online SVM learning: from classification to data description and back*. in *2003 IEEE XIII Workshop on Neural Networks for Signal Processing (IEEE Cat. No.03TH8718)*. 2003.

17. Ridder, D.d., Tax, D.M.J., and Duin, R.P.W. *An experimental comparison of one-class classification methods*. in *Proceedings of the 4th Annual Conference of the Advacned School for Computing and Imaging, Delft*. 1998.

18. Tax, D.M.J. and Duin, R.P.W. *Data description in subspaces*. in *Proceedings 15th International Conference on Pattern Recognition. ICPR-2000*. 2000.

19. Duin, R.P., Juszczak, P., Paclik, P., Pekalska, E., De Ridder, D., Tax, D.M., and Verzakov, S., *Prtools4. 1, a matlab toolbox for pattern recognition.* Delft University of technology, 2007. **2600**.

20. Irigoien, I., Sierra, B., Arenas, C., and #xf3, *Towards Application of One-Class Classification Methods to Medical Data.* The Scientific World Journal, 2014. **2014**: p. 7.

21. Swersky, L., Marques, H.O., Sander, J., Campello, R.J.G.B., and Zimek, A. *On the Evaluation of Outlier Detection and One-Class Classification Methods*. in *2016 IEEE International Conference on Data Science and Advanced Analytics (DSAA)*. 2016.

22. Breunig, M.M., Kriegel, H.-P., Ng, R.T., and Sander, J., *LOF: identifying density-based local outliers.* SIGMOD Rec., 2000. **29**(2): p. 93-104.

23. Goldstein, M. and Uchida, S., *A Comparative Evaluation of Unsupervised Anomaly Detection Algorithms for Multivariate Data.* PLoS One, 2016. **11**(4): p. e0152173.

24. *Anomaly Detection Toolbox*. 2016 [cited 2020 3/25]; Available from: <http://dsmi-lab-ntust.github.io/AnomalyDetectionToolbox/>.

25. Tang, J., Chen, Z., Fu, A.W.-c., and Cheung, D.W. *Enhancing Effectiveness of Outlier Detections for Low Density Patterns*. in *Advances in Knowledge Discovery and Data Mining*. 2002. Berlin, Heidelberg: Springer Berlin Heidelberg.
