# Supplementary material for: A Novel Approach for Continuous Health Status Monitoring and Automatic Detection of Infection Incidences in People With Type 1 Diabetes Using Machine Learning Algorithms (Part 2): A Personalized Digital Infectious Disease Detection Mechanism
Source: J Med Internet Res. 2020 Aug 12;22(8):e18912. doi: 10.2196/18912 (PMC7450372; doi:10.2196/18912)
Supplement: Multimedia Appendix 2 [file jmir_v22i8e18912_app2.docx]

# **Appendix 2 - Detailed Description of the Models Input Features**

## Quadrants of wellness in people with type 1 diabetes

The four quadrants of wellness in people with type 1 diabetes, as shown in the **Figure 1**, tries to defines the state of BG dynamics (blood glucose levels) at any time $t$ using carbohydrate, insulin and physical activity parameters. The first quadrant is called *carbohydrate action*, where the ratio of insulin to carbohydrate is small and the blood glucose levels are elevated (hyperglycemia). This is a normal response of blood glucose dynamics, since consumption of more carbohydrate can elevate blood glucose levels. The second quadrant is called *physical activity action*, where the ratio of insulin to carbohydrate is small but blood glucose levels drops (hypoglycemia). This is a normal response of blood glucose dynamics, since the action of physical activity can derive the patient into hypoglycemia regions even if the patient consumes. The third quadrant is called *insulin action*, where the ratio of insulin to carbohydrate is large (high insulin and low carbohydrate consumption and blood glucose levels drops (hypoglycemia). This is a normal response of blood glucose dynamics, since administration of high insulin with little carbohydrate consumption can derive the patient into hypoglycemia region. The fourth quadrant is called *effect of metabolic change*, where the ratio of insulin to carbohydrate is large (high insulin and low carbohydrate consumption but blood glucose levels are elevated (hyperglycemia). The patient experiences hyperglycemia despite injecting higher amount of insulin and consuming less carbohydrate. This quadrant is an abnormal response due to the effect of metabolic change incurred due to the incidence of infection and stress.


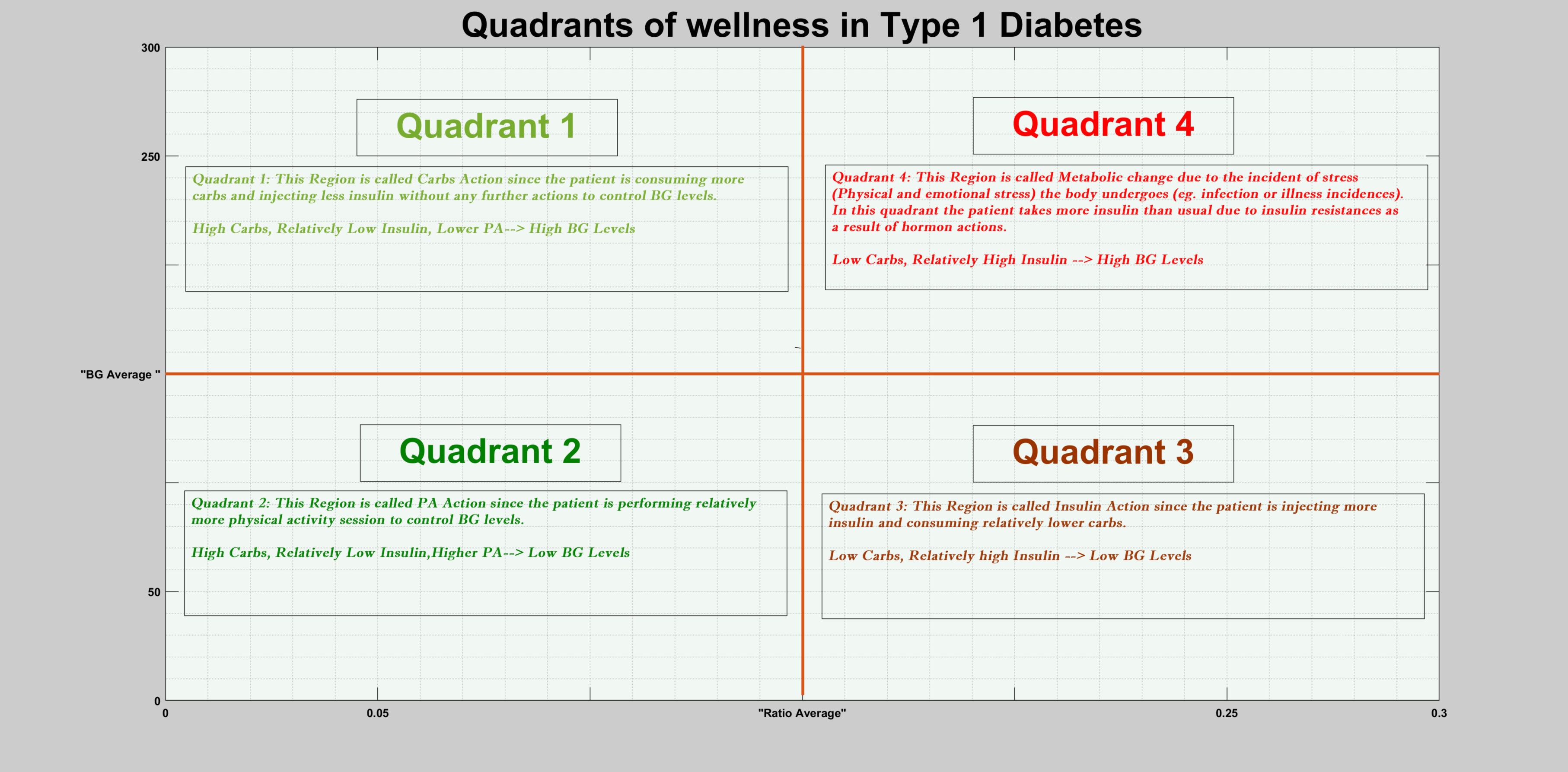
**Figure 1:** Quadrants of wellness in people with type 1 diabetes. The figure depicts the four possible scenarios of different parameters: -carbohydrate action, insulin action, physical activity action, and abnormality due to metabolic change such as infection and stress.

## Atypical Data

Atypical data signify when the data is not uniformly distributed and some region contain high density and other region contain sparse density ^[[1]](#footnote-1)^. In this kind of data, only the boundary between the target and outlier data is the same. However, the exact target density within the boundary could differ. As shown in the **Figure 2-11** below, the scatter plot of the input features, i.e. blood glucose levels vs. insulin to carbohydrate ratio, depicts similar characteristics to the nature of atypical dataset. In this kind of datasets, boundary and domain based method are more preferable than the others. Typically, density methods such as parzen density could suffer in performance degradation to this kind of dataset.

## Description of Input Features

The input features, i.e. average blood glucose levels vs. insulin (bolus) to carbohydrate ratio, used for model training and testing are selected in accordance with the description provided in Woldaregay et al.^[[2]](#footnote-2)^. The **Figures 2-11** depicts the scatter plot of the input features. The data are smoothed using a moving average filter to remove short term noises. For both daily and hourly analysis a moving average filter window size of two days or forty-eight hours were used respectively. Understanding of the data characteristics is essential to select the optimal anomaly detection model to better capture the data distribution during normal situations. The scatter plots of the input features presented in this section incorporates the data of ten different patient years under free living conditions. Five patient years depicting regular years without any significant infection incidences and five patient years with at least one or more incidences of infections1.

### Description of Input Features During the Normal Patient Years

During the normal patients, the input features are characterized to be bounded with similar values of insulin to carbohydrate ratio1. However, from the scatter plot it appears to be a typical in distribution containing regions with high density and low density. The challenge with such kind of data is varying density and rare events that are still normal.

- **The First Patient Year (Normal year)**

1. Daily average blood glucose levels vs. total insulin (bolus) to carbohydrate ratio.

1. Hourly average blood glucose levels vs. total insulin (bolus) to carbohydrate ratio.

**Figure 2:** The first patient year, where there is no incidence of acute infections. Figure (a & b) depicts the daily and hourly scatter plot of the input features.

- **The Second Patient Year (Normal year)**

1. Daily average blood glucose levels vs. total insulin (bolus) to carbohydrate ratio.

1. Hourly average blood glucose levels vs. total insulin (bolus) to carbohydrate ratio.

**Figure 3:** The second patient year, where there is no incidence of acute infections. Figure (a & b) depicts the daily and hourly scatter plot of the input features.

- **The Third Patient Year (Normal year)**

1. Daily average blood glucose levels vs. total insulin (bolus) to carbohydrate ratio.

1. Hourly average blood glucose levels vs. total insulin (bolus) to carbohydrate ratio.

**Figure 4:** The third patient year, where there is no incidence of acute infections. Figure (a & b) depicts the daily and hourly scatter plot of the input features.

- **The Fourth Patient Year (Normal year)**

1. Daily average blood glucose levels vs. total insulin (bolus) to carbohydrate ratio.

1. Hourly average blood glucose levels vs. total insulin (bolus) to carbohydrate ratio.

**Figure 5:** The fourth patient year, where there is no incidence of acute infections. Figure (a & b) depicts the daily and hourly scatter plot of the input features.

- **The Fifth Patient Year (Normal year)**

1. Daily average blood glucose levels vs. total insulin (bolus) to carbohydrate ratio.

1. Hourly average blood glucose levels vs. total insulin (bolus) to carbohydrate ratio.

**Figure 6:** The fifth patient year, where there is no incidence of acute infections. Figure (a & b) depicts the daily and hourly scatter plot of the input features.

### Description of Input Features During the Patient Years with Infection incidences

As can be seen from the scatter plot of the input features, the anomalies from the patient years are visible in both the daily and hourly cases. As described for the normal patient years above, the challenge is mostly modelling the normal portion of the data while minimizing false alarms. There are rare events that are normal response of the blood glucose dynamics and the optimal model is the one that captures those rare events along with the entire normal days.

- **The Six Patient Year (flu)**

1. Daily average blood glucose levels vs. total insulin (bolus) to carbohydrate ratio.

1. Hourly average blood glucose levels vs. total insulin (bolus) to carbohydrate ratio.

**Figure 7:** The six patient year, where the patient was infected with influenza (flu). Figure (a & b) depicts the daily and hourly scatter plot of the input features.

- **The Seventh Patient Year (flu)**

1. Daily average blood glucose levels vs. total insulin (bolus) to carbohydrate ratio.

1. Hourly average blood glucose levels vs. total insulin (bolus) to carbohydrate ratio.

**Figure 8:** The seventh patient year, where the patient was infected with influenza (flu). Figure (a & b) depicts the daily and hourly scatter plot of the input features.

- **The Eighth Patient Year (flu)**

1. Daily average blood glucose levels vs. total insulin (bolus) to carbohydrate ratio.

1. Hourly average blood glucose levels vs. total insulin (bolus) to carbohydrate ratio.

**Figure 9**: The eighth patient year, where the patient was infected with influenza (flu). Figure (a & b) depicts the daily and hourly scatter plot of the input features.

- **The Ninth Patient Year (flu)**

1. Daily average blood glucose levels vs. total insulin (bolus) to carbohydrate ratio.

1. Hourly average blood glucose levels vs. total insulin (bolus) to carbohydrate ratio.

**Figure 10**: The ninth patient year, where the patient was infected with influenza (flu) and light and mild common cold without fever. Figure (a & b) depicts the daily and hourly scatter plot of the input features.

- **The Tenth Patient Year (Flu)**

1. Daily average blood glucose levels vs. total insulin (bolus).

1. Hourly average blood glucose levels vs. total insulin (bolus).

**Figure 11**: The tenth patient year, where the patient was infected with influenza (flu). Figure (a & b) depicts the daily and hourly scatter plot of average blood glucose levels vs. total insulin (bolus).

1. Tax, D.M.J., *One-class classification: Concept learning in the absence of counter-examples*, in *Technische Universiteit Delft*. 2002. [↑](#footnote-ref-1)
2. Woldaregay, A.Z., Årsand, E., Albers, D., Launonen, I., Holubová, A., and Hartvigsen, G., *Towards Detecting Infection Incidences in People with Type 1 Diabetes Using Self-Recorded Data: A Novel Framework for a Digital Infectious Disease Detection Mechanism.* JMIR Preprints, 2020. **26/03/2020:18911**. [↑](#footnote-ref-2)
