# Supplementary material for: A Novel Approach for Continuous Health Status Monitoring and Automatic Detection of Infection Incidences in People With Type 1 Diabetes Using Machine Learning Algorithms (Part 2): A Personalized Digital Infectious Disease Detection Mechanism
Source: J Med Internet Res. 2020 Aug 12;22(8):e18912. doi: 10.2196/18912 (PMC7450372; doi:10.2196/18912)
Supplement: Multimedia Appendix 3 [file jmir_v22i8e18912_app3.docx]

# **Appendix 3-Score Plot of the Models for Each Patient Year**

The evaluations of the models were carried out based on each patient year depending on two specified time-window; hourly and daily. The data were smoothed using a moving average with a window size of two days (forty-eight hours). The models were trained using a target dataset (regular/normal days) and tested on the whole patient year containing both the target (regular/normal days) and non-target (infection period). A training sample size of 120 days, which are randomly selected from the patient year excluding the infection period, were used train the models and the score of each models for the whole patient year were plotted. Regarding the unsupervised methods, the whole patient year was presented during testing. The portion rejected by the models in all the figures represent the infection period.

1. **Semi-supervised (One-class classifiers)**

### Daily

#### The First Case of Infection (flu)

##### Boundary and Domain-Based Method

**Figure 1**: The score of the boundary and domain-based method on the whole patient year.

##### Density-Based Method

**Figure 2:** The score of the density-based method on the on the whole patient year.

##### Reconstruction-Based Method

**Figure 3**: The score of the reconstruction-based method on the whole patient year.

#### The Second Case of Infection (flu)

##### Boundary and Domain-Based Method

**Figure 4**: The score of the boundary and domain-based method on the whole patient year.

##### Density-Based Method

**Figure 5**: The score of the density-based method on the on the whole patient year.

##### Reconstruction-Based Method

**Figure 6**: The score of the reconstruction-based method on the whole patient year.

#### The Third Case of Infection (flu)

##### Boundary and Domain-Based Method

**Figure 7**: The score of the boundary and domain-based method on the whole patient year.

##### Density-Based Method

**Figure 8**: The score of the density-based method on the whole patient year.

##### Reconstruction-Based Method

**Figure 9**: The score of the reconstruction-based method on the whole patient year.

#### The Fourth Case of *Infection* (flu)

##### Boundary and Domain-Based Method

**Figure 10**: The score of the boundary and domain-based method on the whole patient year.

##### Density-Based Method

**Figure 11**: The score of the density-based method on the whole patient year.

##### Reconstruction-Based Method

**Figure 12**: The score of the reconstruction-based method on the whole patient year.

### Hourly

#### The First Case of Infection (flu)

##### Boundary and Domain-Based Method


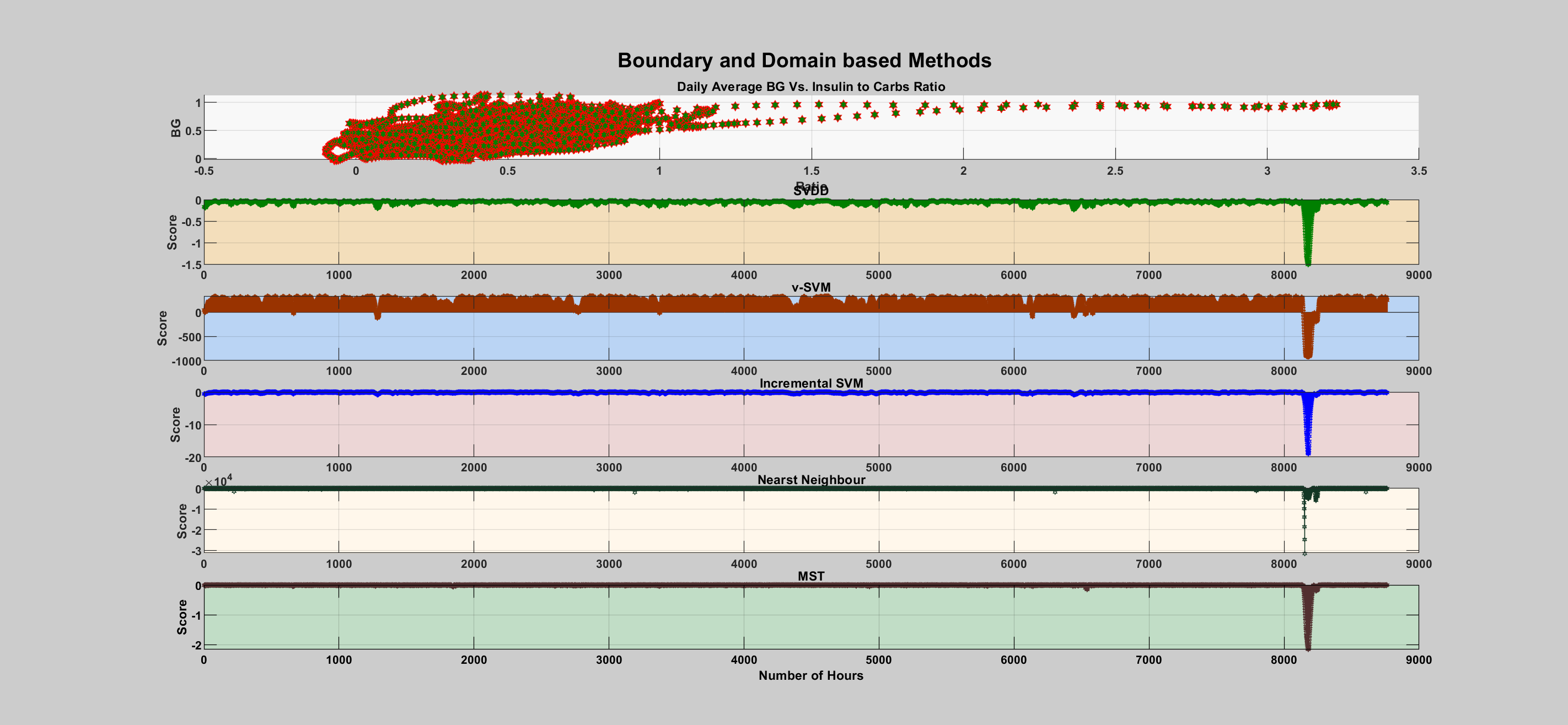


**Figure 13**: The score of the boundary and domain-based method on the whole patient year.

##### Density-Based Method


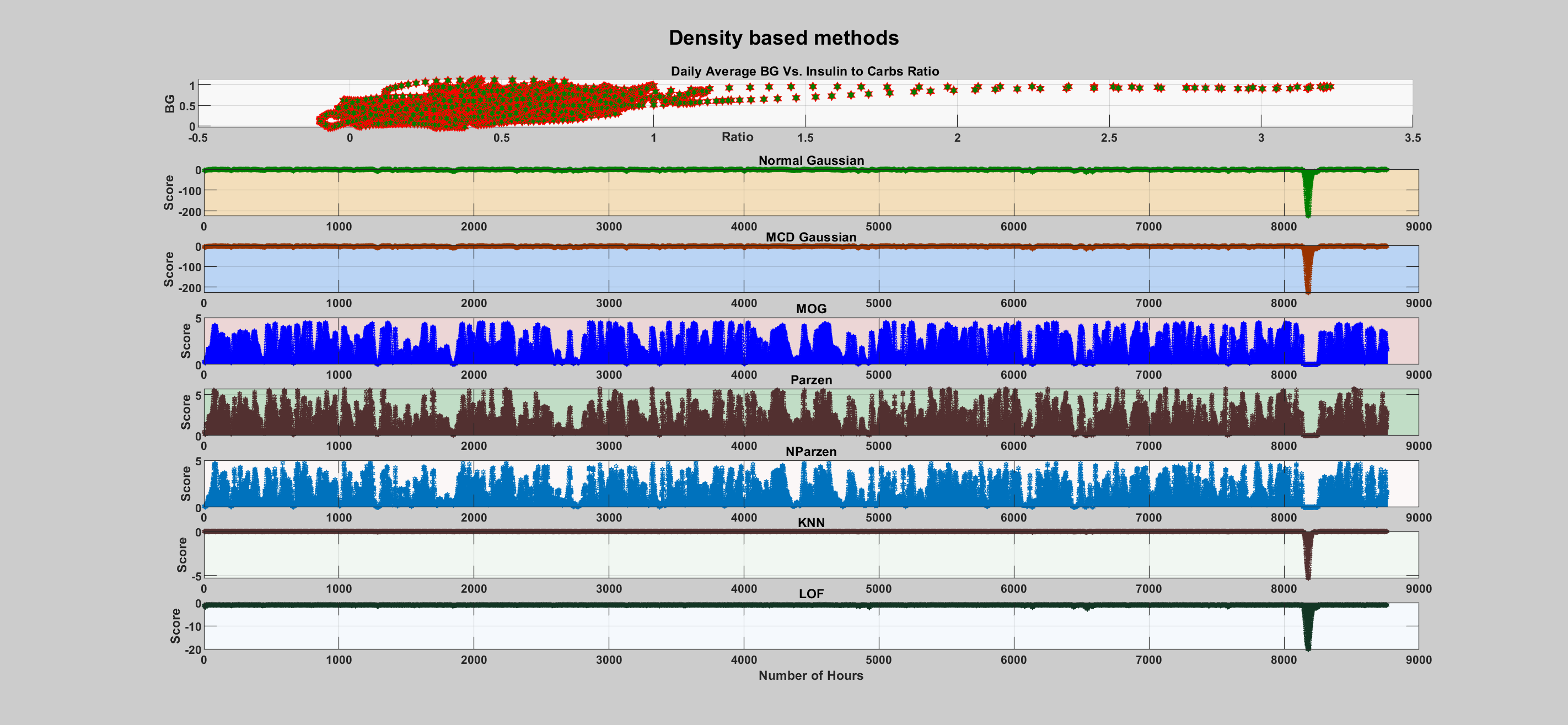


**Figure 14**: The score of the density-based method on the whole patient year.

##### Reconstruction-Based Method


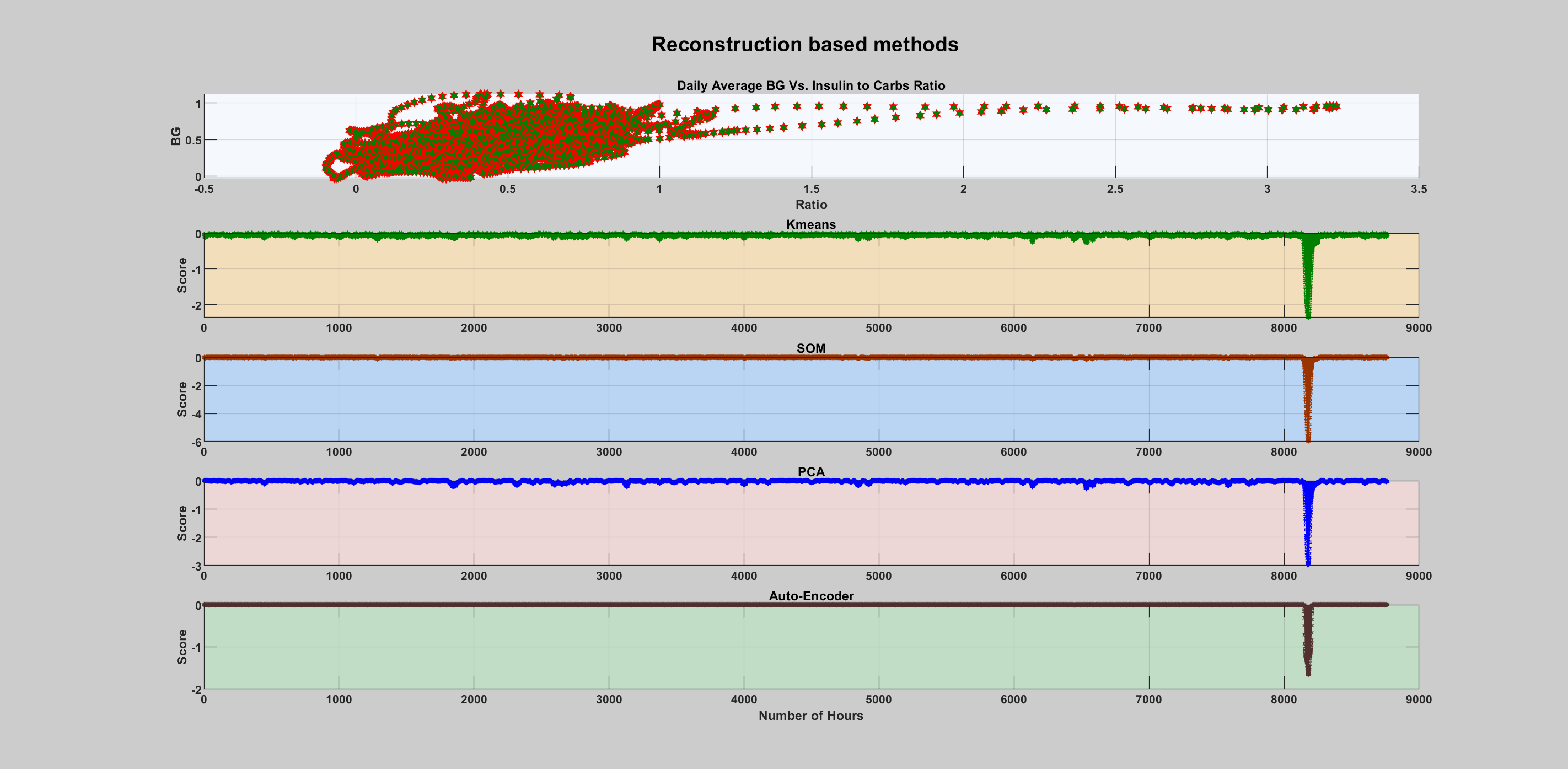


**Figure 15**: The score of the reconstruction-based method on the whole patient year.

#### The Second Case of Infection (flu)

##### Boundary and Domain-Based Method


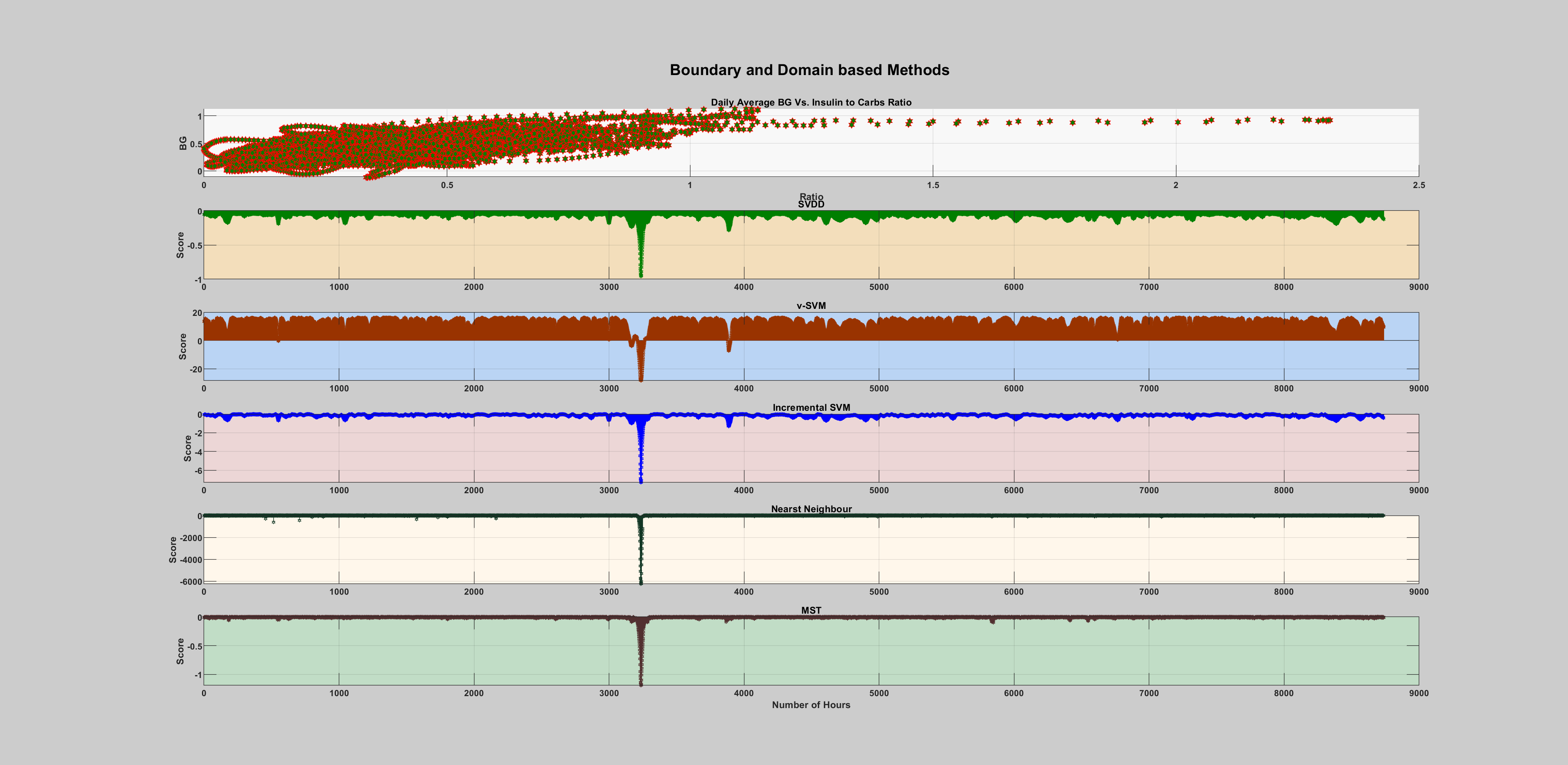


**Figure 16**: The score of the boundary and domain-based method on the whole patient year.

##### Density-Based Method


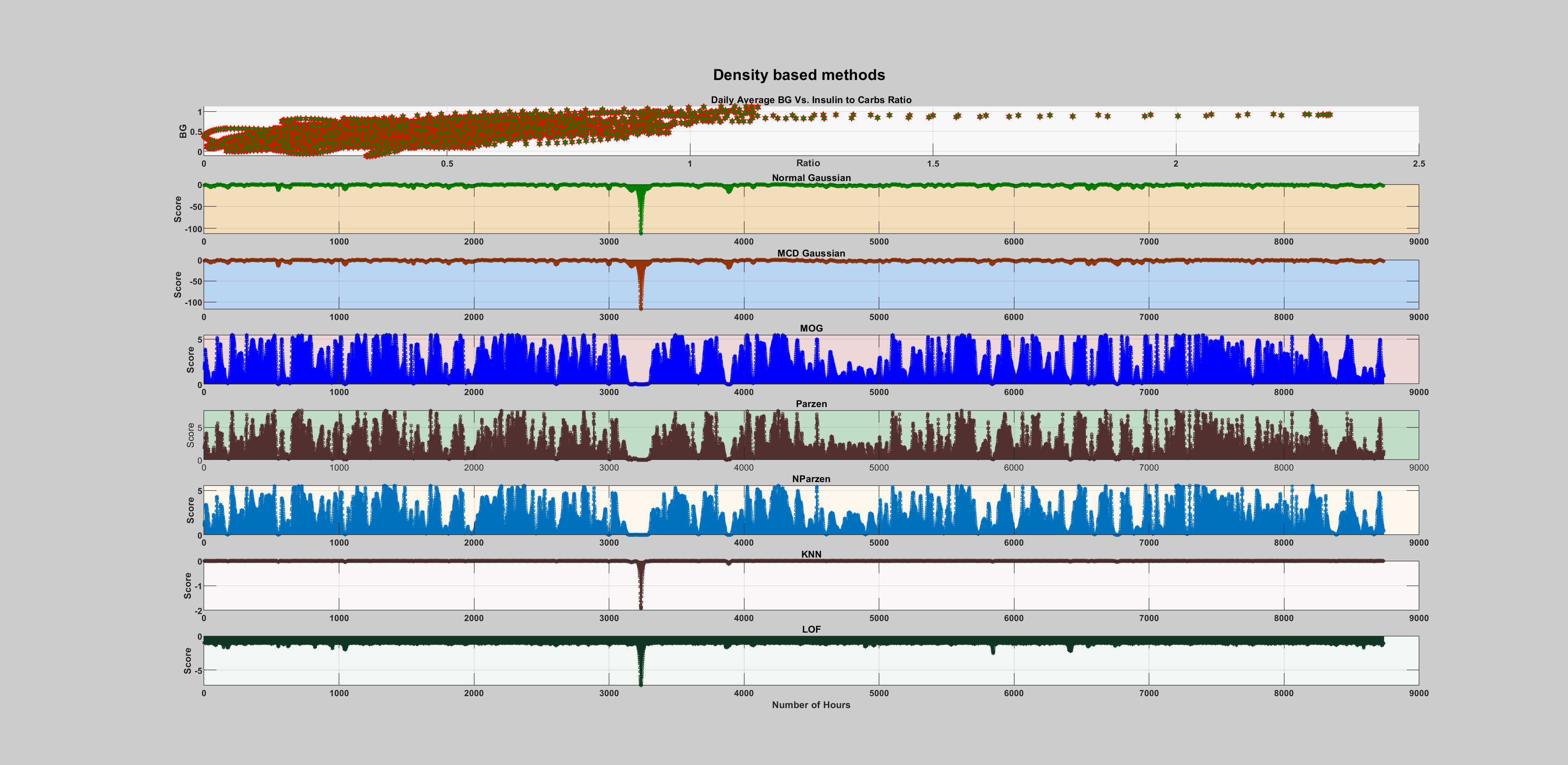


**Figure 17**: The score of the density-based method on the whole patient year.

##### Reconstruction-Based Method


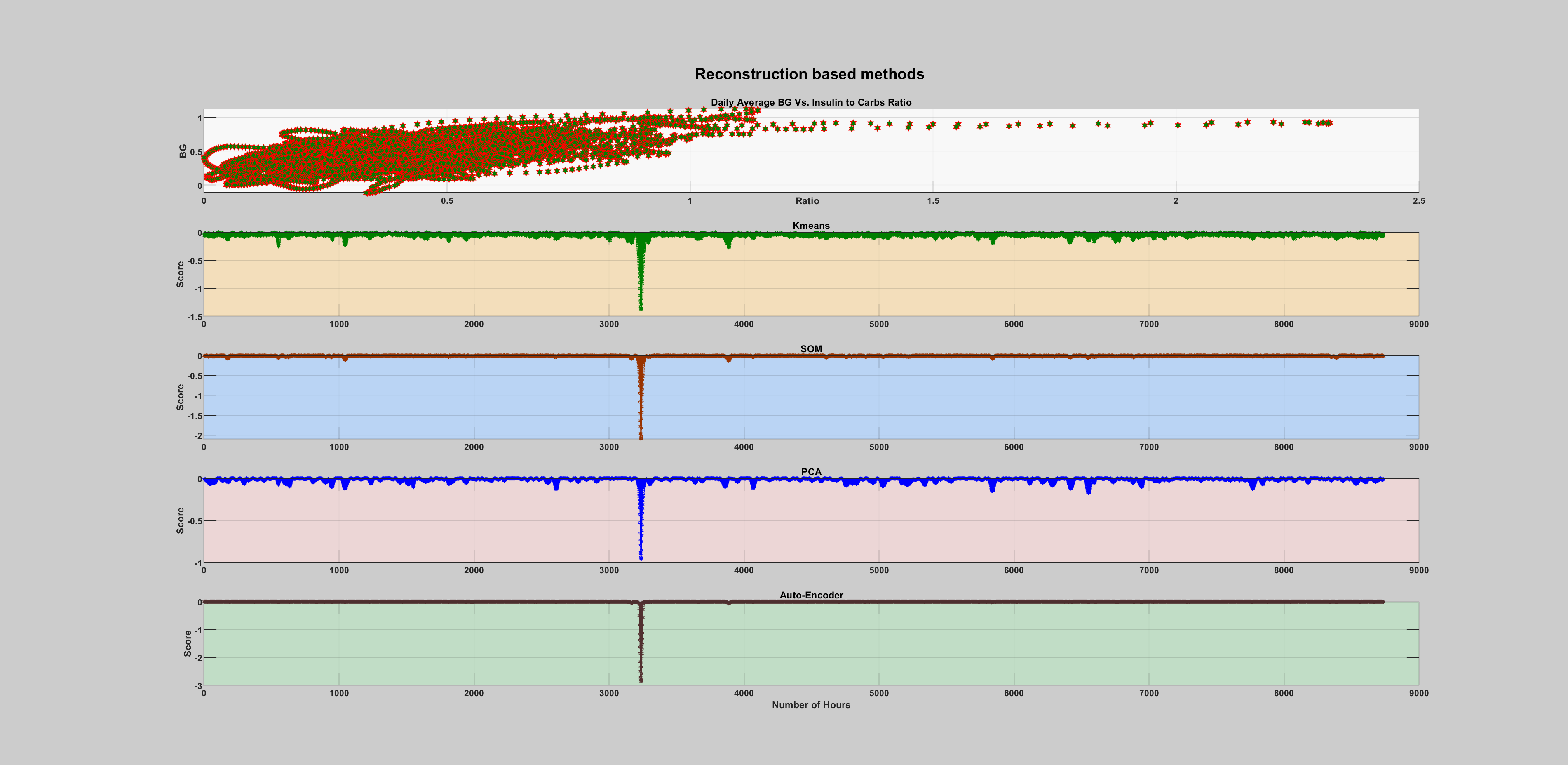


**Figure 18**: The score of the reconstruction-based method on the whole patient year.

#### The Third Case of Infection (flu)

##### Boundary and Domain-Based Method


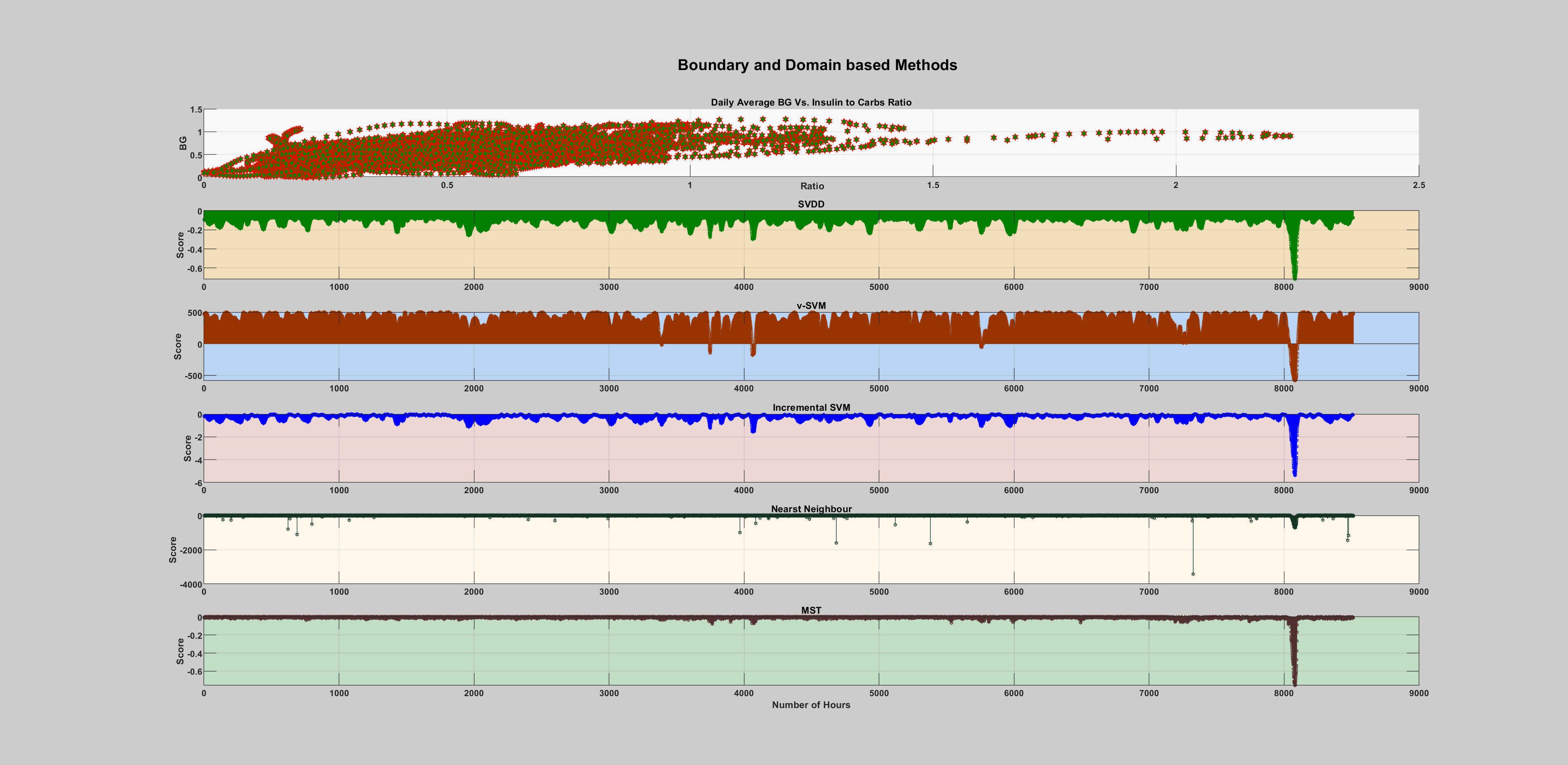


**Figure 19**: The score of the boundary and domain-based method on the whole patient year.

##### Density-Based Method


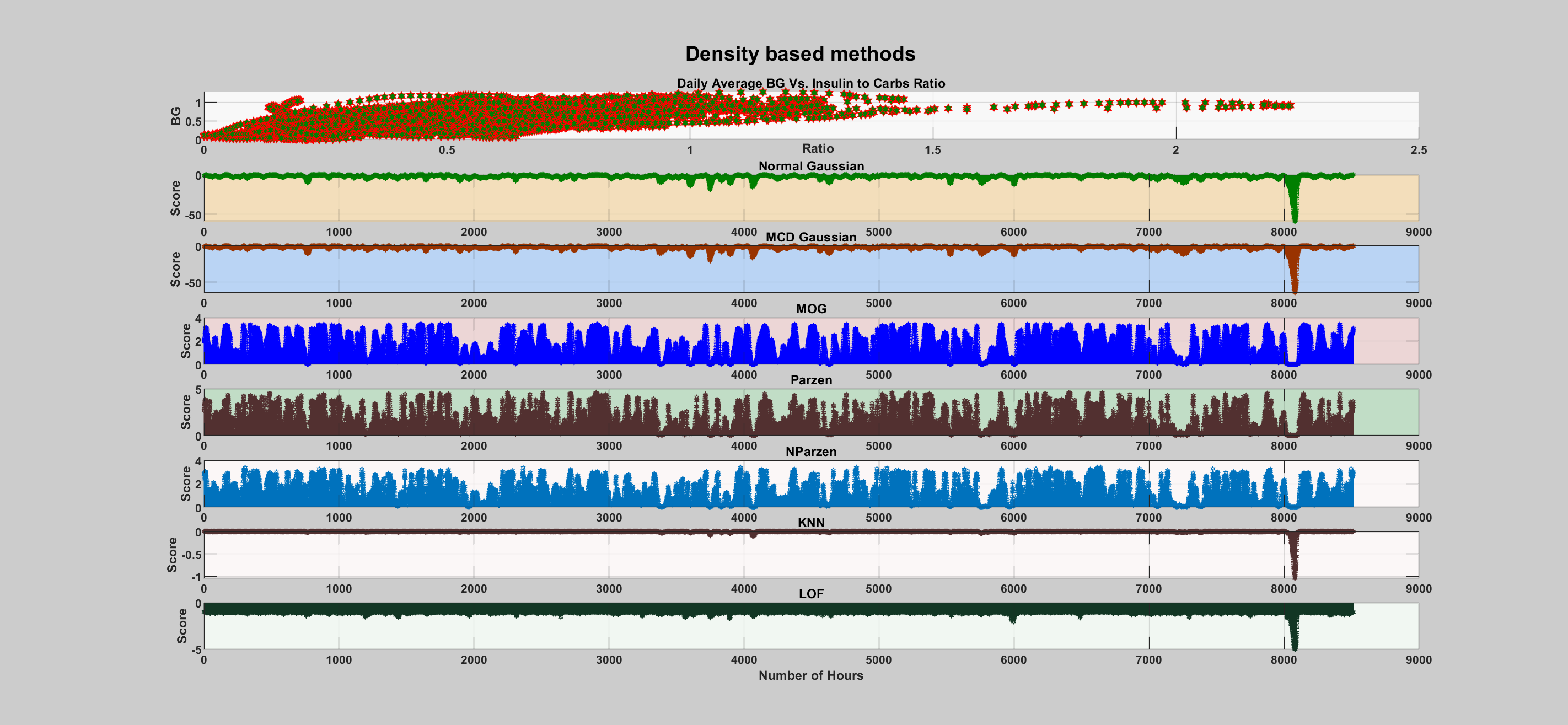


**Figure 20**: The score of the density-based method on the whole patient year.

##### Reconstruction-Based Method


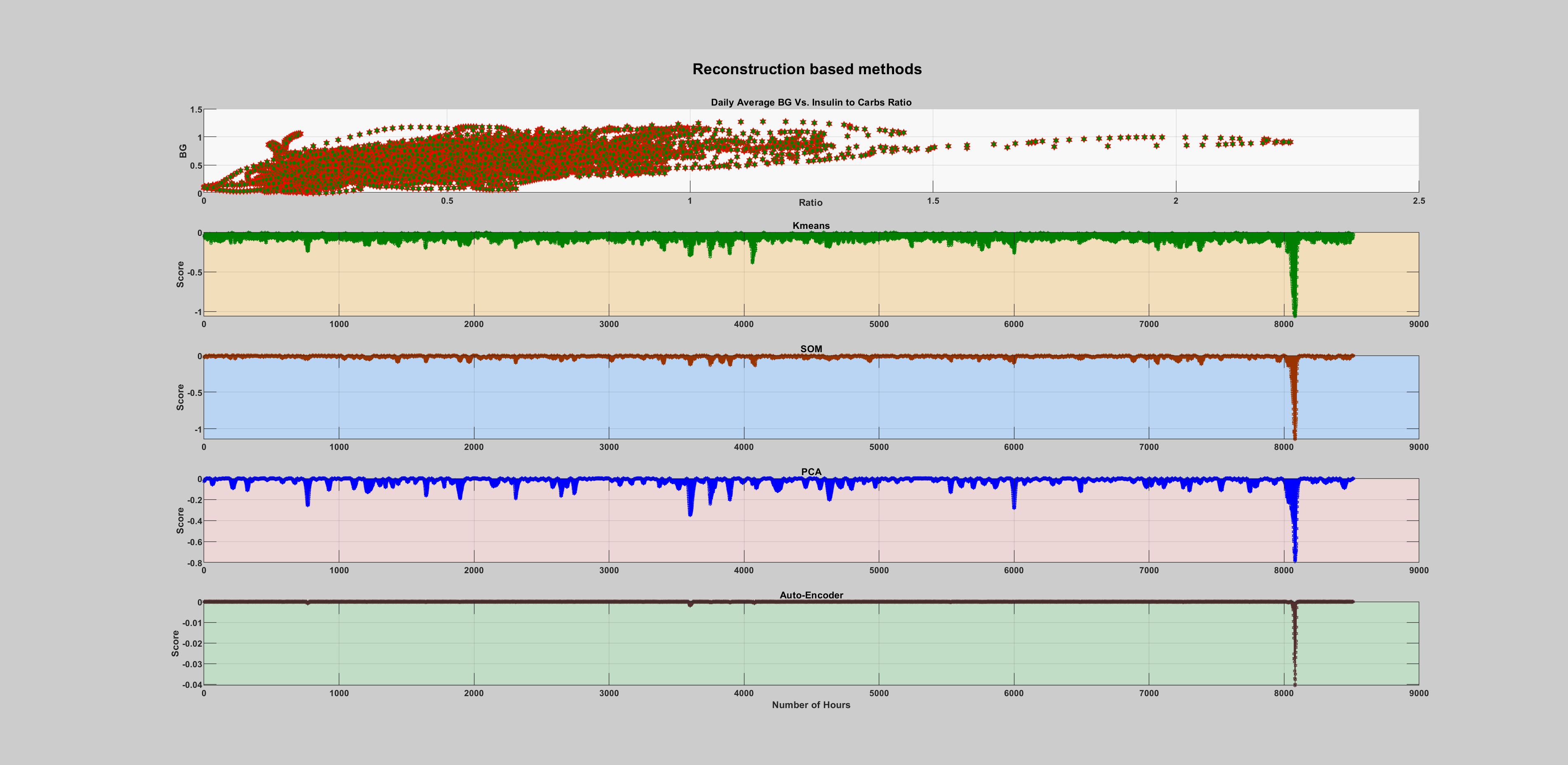


**Figure 21**: The score of the reconstruction-based method on the whole patient year.

#### The Fourth Case of Infection (flu)

##### Boundary and Domain-Based Method


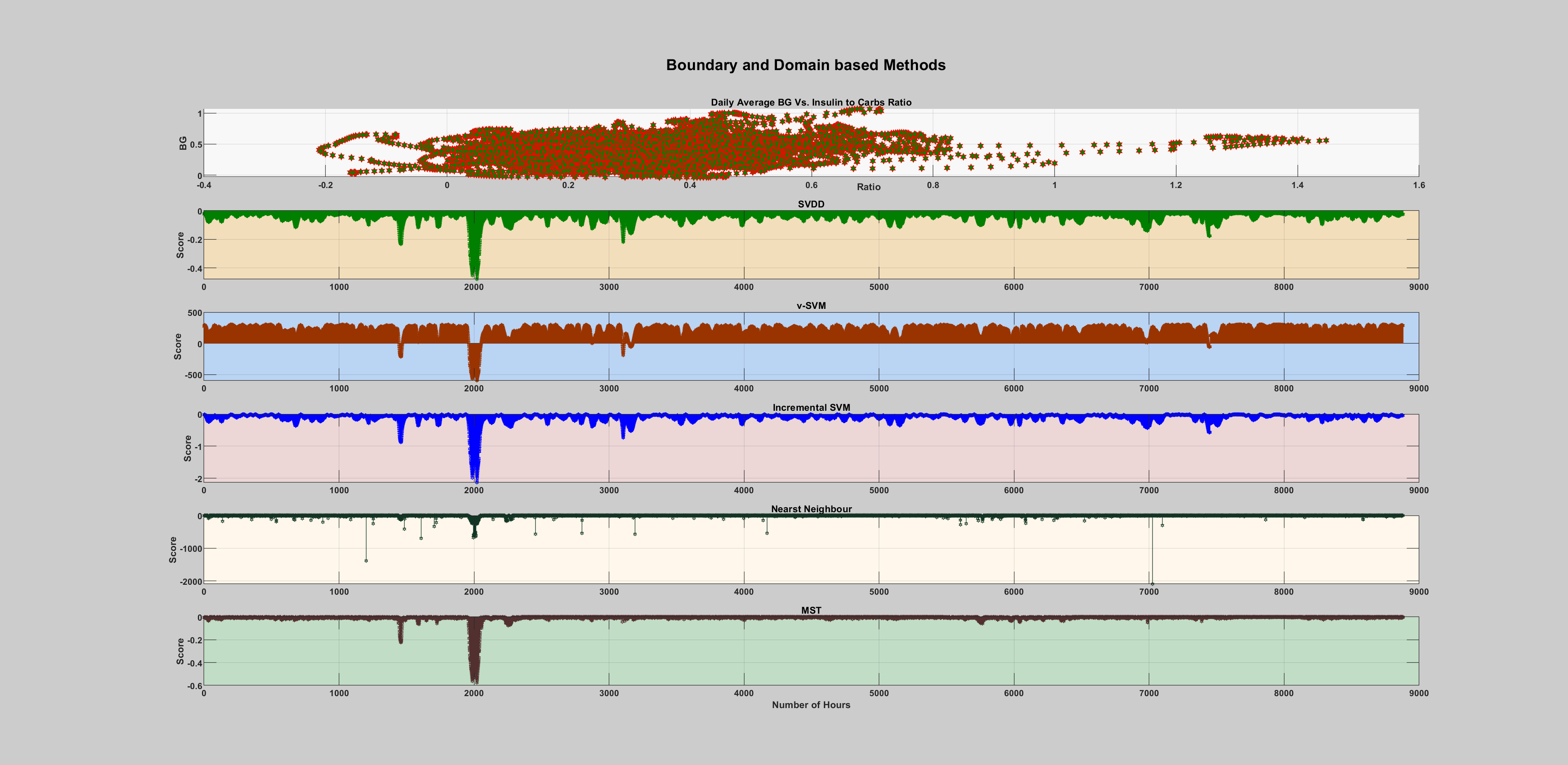


**Figure 22**: The score of the boundary and domain-based method on the whole patient year.

##### Density-Based Method


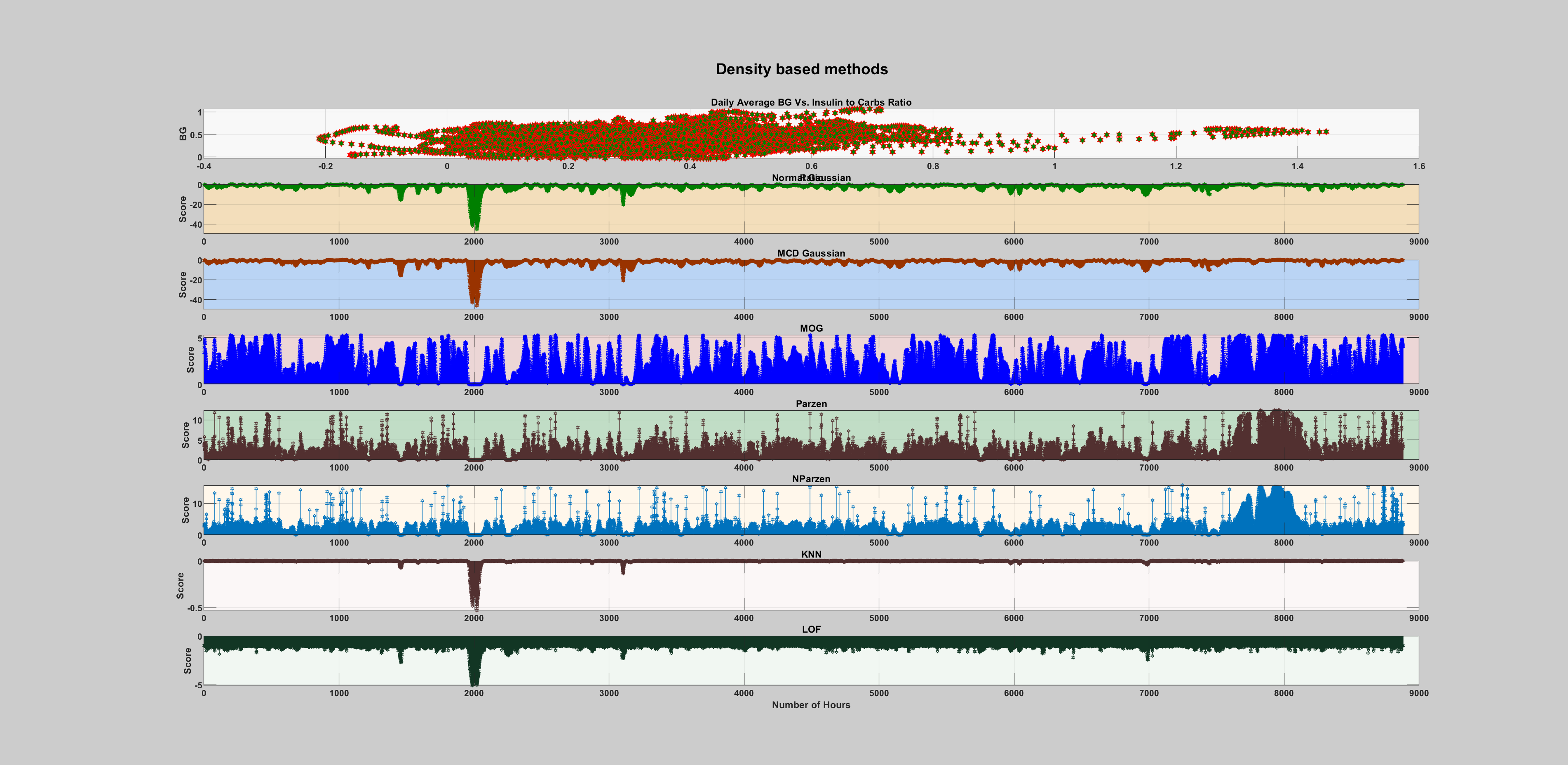


**Figure 23**: The score of the density-based method on the whole patient year.

##### Reconstruction-Based Method


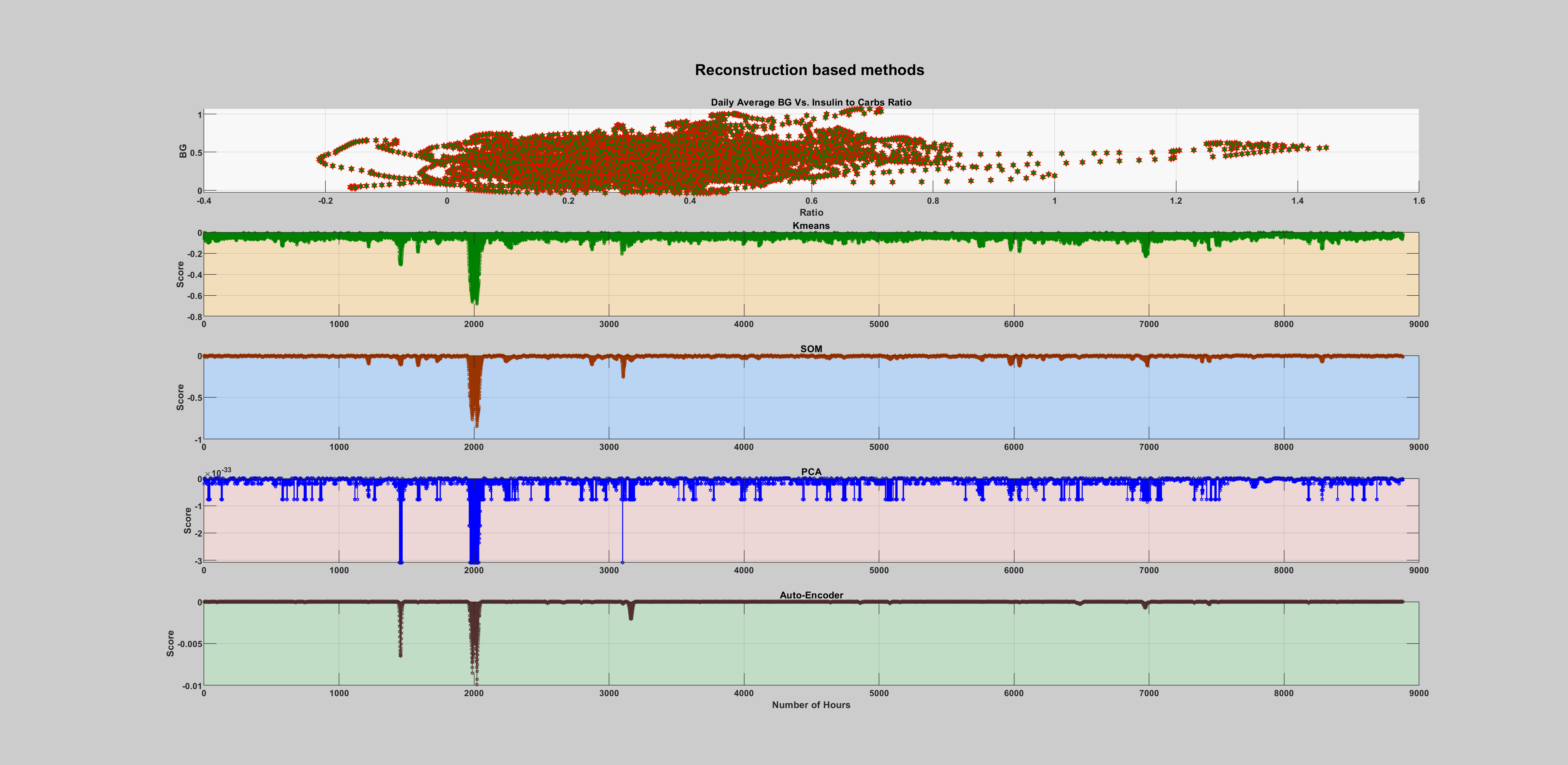


**Figure 24**: The score of the reconstruction-based method on the whole patient year.

1. **Unsupervised Approach**

### Daily

#### The First Case of Infection (flu)

**Figure 25**: The score of unsupervised methods, LOF and COF, on the whole patient year. The value of k is set to be 30 data points.

#### The Second Case of Infection (flu)

**Figure 26**: The score of unsupervised methods, LOF and COF, on the whole patient year. The value of k is set to be 30 data points.

#### The Third Case of Infection (flu)

**Figure 27**: The score of unsupervised methods, LOF and COF, on the whole patient year. The value of k is set to be 30 data points.

#### The Fourth Case of Infection (flu)

**Figure 28**: The score of unsupervised methods, LOF and COF, on the whole patient year. The value of k is set to be 30 data points.

### Hourly

#### The First Case of Infection (flu)

**Figure 29**: The score of unsupervised methods, LOF and COF, on the whole patient year. The value of k is set to be 240 data points.

#### The Second Case of Infection (flu)

**Figure 30**: The score of unsupervised methods, LOF and COF, on the whole patient year. The value of k is set to be 240 data points.

#### The Third Case of Infection (flu)

**Figure 31**: The score of unsupervised methods, LOF and COF, on the whole patient year. The value of k is set to be 240 data points.

#### The Fourth Case of Infection (flu)

**Figure 32**: The score of unsupervised methods, LOF and COF, on the whole patient year. The value of k is set to be 240 data points.
