# Supplementary material for: A Novel Approach for Continuous Health Status Monitoring and Automatic Detection of Infection Incidences in People With Type 1 Diabetes Using Machine Learning Algorithms (Part 2): A Personalized Digital Infectious Disease Detection Mechanism
Source: J Med Internet Res. 2020 Aug 12;22(8):e18912. doi: 10.2196/18912 (PMC7450372; doi:10.2196/18912)
Supplement: Multimedia Appendix 4 [file jmir_v22i8e18912_app4.docx]

# **Appendix 4 - Model Evaluations – Performance of the Models for Each Patient Year**

The one-class classifier models presented in this section consists of three categories; boundary and domain based, density based, and reconstruction based. The model evaluation is carried on a dataset that represent a daily and hourly scope. The models were evaluated using twenty times fivefold stratified cross-validation. During training phase, only the regular/normal day measurements were used to train the models. During testing phase, a dataset containing both regular/normal and infection day measurements were used. The performances are reported as average and standard deviation of the twenty rounds. Performance metrics like area under the ROC curve (AUC), specificity, and F1-score were used to evaluate the model performances. Two version of the same data was used to assess the performance; raw data and smoothed data. The smoothed data is the filtered version of the raw data using a moving average filter of 2-days window size. The models were compared considering required sample sizes to obtain satisfactory performance. The performance of the models is given in the Table 1-7 below for different individuals and infection years.

## **Daily**

##### The First Case of Infection (Flu)

**Table 1 (a)**: Average and standard deviation of AUC, specificity, F1-score for the raw dataset (without smoothing) and different sample size.

| **Fraction = 0.01** | | | | | | | | | | | | |
| --- | --- | --- | --- | --- | --- | --- | --- | --- | --- | --- | --- | --- |
| Models | **Boundary and Domain-Based Method** | | | | | | | | | | | |
|  | 1 Month | | | 2 Months | | | 3 Months | | | 4 Months | | |
|  | **AUC** | **Specificity** | **F1** | **AUC** | **Specificity** | **F1** | **AUC** | **Specificity** | **F1** | **AUC** | **Specificity** | **F1** |
| SVDD | 90.7 (8.8) | 71.7 (7.7) | **73.6 (5.5)** | 93.4 (6.2) | 81.7 (5.0) | 87.4 (8.1) | 96.4 (2.9) | 87.8 (3.3) | 91.3 (6.0) | 94.6 (3.7) | 81.7 (5.0) | 90.0 (4.6) |
| incsvdd | 90.4 (8.9) | 66.7 (7.5) | 72.7 (4.9) | 91.8 (5.9) | 66.7 (7.5) | 84.4 (3.2) | 95.8 (2.9) | 70.0 (7.1) | 85.4 (1.2) | 93.7 (3.6) | 55 (10.7) | 81.0 (2.7) |
| $\boldsymbol{\upsilon}$**-**$\boldsymbol{SVM}$ | 93.1 (6.0) | 63 (10.6) | 78.9 (6.2) | 96.5 (2.3) | 81.9 (4.7) | **90.7 (3.4)** | 97.9 (1.5) | 88.9 (0.0) | **94.1 (2.0)** | 96.2 (2.3) | 83.3 (0.0) | **91.7 (1.4)** |
| Nearest Neighbour | 74.2 (9.3) | 38.3 (7.7) | 61.0 (4.7) | 89.5 (9.3) | 20.0 (6.7) | 70.0 (4.6) | 90.1 (6.6) | 11.1 (18) | 69.2 (3.8) | 92.8 (3.3) | 33.3 (0.0) | 75.1 (0.4) |
| MST | 89.4 (8.1) | 50.0 (0.0) | 62.7 (6.6) | 95.4 (5.6) | 61.7 (7.7) | 82.3 (5.9) | 96.6 (2.7) | 68.9 (4.5) | 83.6 (4.7) | 94.1 (2.8) | 55.0 (7.7) | 80.6 (2.3) |
|  | **Density-Based Method** | | | | | | | | | | | |
| Gaussian | 90.6 (7.1) | 60.0 (8.2) | 68.8 (8.4) | 95.4 (4.6) | 70.0 (6.7) | 85.3 (4.6) | 97.3 (2.5) | 80.0 (4.5) | 89.2 (3.3) | 95.5 (3.2) | 66.7 (0.0) | 84.5 (2.0) |
| MoG | 88.1 (9.9) | 80.1 (17.3) | 67.8 (16.4) | 93.1 (7.1) | 75.8 (14.8) | 82.5 (10.1) | 95.6 (3.4) | 80.2 (7.5) | 86.0 (6.7) | 93.7 (3.9) | 68.7 (11.6) | 84.2 (5.7) |
| MCD Gaussian | 89.0 (8.5) | 55.0 (7.7) | 66.4 (9.0) | 94.0 (4.6) | 68.3 (5.0) | 84.6 (6.3) | 97.0 (2.7) | 80.0 (4.5) | 89.9 (2.4) | 94.5 (3.2) | 65.0 (5.0) | 84.0 (3.2) |
| Parzen | 89.0 (9.2) | 70.0 (6.7) | 70.7 (5.9) | 94.6 (4.9) | 83.3 (0.0) | 87.9 (6.3) | 97.2 (2.4) | 88.9 (0.0) | 90.5 (5.9) | 95.2 (2.9) | 83.3 (0.0) | 88.9 (3.3) |
| Naive Parzen | 90.1 (7.6) | 55 (10.7) | 65.0 (5.0) | 95.7 (3.9) | 76.7 (8.2) | 87.2 (3.5) | 98.3 (1.4) | 88.9 (0.0) | **93.6 (2.4)** | 96.8 (2.1) | 83.3 (0.0) | 90.7 (2.0) |
| k-NN | 91.8 (6.9) | 50.0 (0.0) | 66.0 (2.0) | 95.6 (3.1) | 81.7 (5.0) | **90.9 (3.2)** | 97.9 (1.6) | 88.9 (0.0) | 93.5 (3.7) | 97.0 (2.2) | 83.3 (0.0) | **92.0 (1.0)** |
| LOF | 88.5 (6.1) | 66.7 (7.5) | **72.7 (4.9)** | 97.0 (1.9) | 71.7 (7.7) | 86.1 (2.4) | 96.8 (2.8) | 78.9 (3.3) | 88.7 (2.8) | 92.6 (4.8) | 50.0 (0.0) | 79.3 (2.6) |
|  | **Reconstruction-Based Method** | | | | | | | | | | | |
| PCA | 87.8 (11.9) | 50.0 (7.5) | 62.4 (8.5) | 93.5 (6.2) | 51.7 (5.0) | 78.2 (4.1) | 93.6 (4.7) | 60 (10.2) | 81.8 (4.4) | 91.3 (5.2) | 46.7 (6.7) | 78.7 (2.3) |
| Auto – encoder | 82.2 (12.0) | 57.9 (15.3) | 64.7 (12.0) | 88.2 (9.5) | 61.6 (14.0) | 81.4 (7.1) | 93.4 (5.7) | 74.4 (11) | 86.4 (5.9) | 88.4 (8.8) | 61.3 (14.3) | 82.7 (5.7) |
| SOM | 86.9 (9.4) | 78.3 (13.3) | 66.7 (16.9) | 92.8 (7.3) | 64.2 (12.4) | 80.9 (7.0) | 95.8 (3.7) | 80.1 (6.3) | 86.9 (5.5) | 92.2 (4.1) | 76.5 (9.0) | 87.5 (4.5) |
| K-means | 91.8 (6.9) | 65.0 (9.0) | **71.8 (5.1)** | 96.0 (2.4) | 83.3 (0.0) | **91.5 (2.8)** | 97.6 (1.6) | 88.9 (0.0) | **93.5 (3.7)** | 96.2 (2.2) | 83.3 (0.0) | **91.5 (1.6)** |

**Table 1 (b):** Average and standard deviation of AUC, specificity, F1-score for smoothed version of the data with a two-days moving average filter and different sample size.

| **Fraction = 0.01** | | | | | | | | | | | | |
| --- | --- | --- | --- | --- | --- | --- | --- | --- | --- | --- | --- | --- |
| Models | **Boundary and Domain-Based Method** | | | | | | | | | | | |
|  | 1 Month | | | 2 Months | | | 3 Months | | | 4 Months | | |
|  | **AUC** | **Specificity** | **F1** | **AUC** | **Specificity** | **F1** | **AUC** | **Specificity** | **F1** | **AUC** | **Specificity** | **F1** |
| SVDD | 99.6 (1.3) | 100 (0.0) | 93.6 (15.2) | 100 (0.0) | 100 (0.0) | 94.8 (10.1) | 100 (0.0) | 100 (0.0) | 97.0 (4.1) | 100 (0.0) | 100 (0.0) | 96.9 (4.0) |
| incsvdd | 99.6 (1.3) | 100 (0.0) | 93.6 (15.2) | 100 (0.0) | 100 (0.0) | 97.1 (6.3) | 100 (0.0) | 100 (0.0) | 97.6 (4.1) | 100 (0.0) | 100 (0.0) | 98.3 (2.8) |
| $\boldsymbol{\upsilon}$**-**$\boldsymbol{SVM}$ | 100 (0.0) | 99.5 (2.9) | **98.9 (3.2)** | 100 (0.0) | 100 (0.0) | **99.1 (2.6)** | 100 (0.0) | 100 (0.0) | **99.4 (1.7)** | 100 (0.0) | 100 (0.0) | **99.6 (1.2)** |
| Nearest Neighbour | 98.1 (3.9) | 58.3 (15.4) | 72.3 (9.9) | 86.9 (12.5) | 16.7 (22.4) | 70.5 (5.3) | 88.1 (6.5) | 54.4 (22.5) | 80.0 (8.6) | 92.4 (5.3) | 8.3 (17.1) | 69.0 (4.8) |
| MST | 98.5 (2.4) | 85.0 (5.0) | 85.5 (2.1) | 99.7 (0.8) | 100 (0.0) | 97.1 (6.3) | 99.9 (0.4) | 97.8 (4.5) | 97.2 (4.0) | 99.7 (0.8) | 100 (0.0) | 97.0 (7.9) |
|  | **Density-Based Method** | | | | | | | | | | | |
| Gaussian | 100 (0.0) | 98.3 (5.0) | 92.1 (15.2) | 100 (0.0) | 100 (0.0) | 97.1 (6.3) | 99.8 (0.7) | 100 (0.0) | 97.6 (4.1) | 99.4 (1.7) | 100 (0.0) | 97.0 (7.9) |
| MoG | 98.6 (3.2) | 99.8 (1.7) | 88.5 (16.8) | 99.6 (1.2) | 100 (0.0) | 92.2 (11.1) | 99.7 (0.7) | 99.8 (1.4) | 94 (10.3) | 99.3 (2.0) | 99.9 (1.2) | 94.4 (11.8) |
| MCD Gaussian | 98.9 (2.2) | 91.7 (8.4) | **90.9 (7.7)** | 100 (0.0) | 100 (0.0) | **98.0 (6.0)** | 99.5 (1.1) | 96.7 (5.1) | 96.6 (5.9) | 99.4 (1.7) | 88.3 (7.7) | 92.0 (6.8) |
| Parzen | 99.6 (1.3) | 100 (0.0) | 87.7 (17.0) | 100 (0.0) | 100 (0.0) | 95.1 (8.0) | 100 (0.0) | 100 (0.0) | 94.6 (9.8) | 99.9 (0.4) | 100 (0.0) | 94.6 (12.3) |
| Naive Parzen | 99.2 (2.5) | 100 (0.0) | 94.7 (11.1) | 100 (0.0) | 100 (0.0) | 93.8 (11.0) | 99.6 (1.1) | 100 (0.0) | 97.5 (5.0) | 100 (0.0) | 100 (0.0) | **98.7 (2.7)** |
| k-NN | 98.1 (3.9) | 68.3 (5.0) | 75.2 (4.3) | 100 (0.0) | 100 (0.0) | **98.0 (6.0)** | 100 (0.0) | 100 (0.0) | **98.8 (3.8)** | 100 (0.0) | 100 (0.0) | 97.7 (4.7) |
| LOF | 98.6 (2.9) | 75.0 (13.5) | 80.2 (10.8) | 100 (0.0) | 100 (0.0) | **98.0 (6.0)** | 100 (0.0) | 100 (0.0) | 96.9 (5.0) | 99.7 (0.8) | 100 (0.0) | 97.4 (7.9) |
|  | **Reconstruction-Based Method** | | | | | | | | | | | |
| PCA | 98.9 (2.2) | 85.0 (5.0) | **85.5 (2.1)** | 99.2 (1.3) | 85.0 (5.0) | **91.4 (2.7)** | 98.6 (1.9) | 88.9 (0.0) | 92.2 (6.0) | 97.8 (2.2) | 83.3 (0.0) | 89.1 (9.7) |
| Auto – encoder | 97.4 (6.0) | 89.1 (13.0) | 86.0 (14.2) | 98.5 (3.2) | 94.5 (9.6) | 91.8 (9.4) | 99.2 (2.4) | 93.7 (10.2) | 93.7 (8.3) | 98.6 (3.8) | 94.4 (9.5) | 93.7 (9.7) |
| SOM | 99.3 (1.9) | 99.9 (1.2) | 84.7 (19.8) | 99.8 (0.7) | 100 (0.0) | 91.4 (9.6) | 99.9 (0.3) | 100 (0.0) | 95.2 (7.9) | 99.6 (1.3) | 100 (0.0) | 93.4 (12.1) |
| K-means | 99.2 (2.5) | 85.0 (11.7) | 87.0 (10.4) | 100 (0.0) | 100 (0.0) | 97.1 (6.3) | 100 (0.0) | 100 (0.0) | **98.8 (3.8)** | 100 (0.0) | 100 (0.0) | **99.2 (2.5)** |

1. The Second Case of Infection (Flu)

**Table 2 (a)**: Average and standard deviation of AUC, specificity, F1-score for the raw dataset (without smoothing) and different sample size.

| **Fraction = 0.01** | | | | | | | | | | | | |
| --- | --- | --- | --- | --- | --- | --- | --- | --- | --- | --- | --- | --- |
| Models | **Boundary and Domain-Based Method** | | | | | | | | | | | |
|  | 1 Month | | | 2 Months | | | 3 Months | | | 4 Months | | |
|  | **AUC** | **Specificity** | **F1** | **AUC** | **Specificity** | **F1** | **AUC** | **Specificity** | **F1** | **AUC** | **Specificity** | **F1** |
| SVDD | 78.1 (19.2) | 25 (25.1) | 53.3 (5.2) | 90 (11.4) | 23.3 (20.1) | 69.5 (3.7) | 93.5 (7.0) | 28.9 (20.1) | **72.7 (3.7)** | 87.0 (5.6) | 23.3 (20.1) | **71.6 (4.1)** |
| incsvdd | 67.1 (18.0) | 18.3 (5.0) | 52.3 (9.4) | 87.5 (14.2) | 23.3 (20.1) | **70.3 (2.8)** | 91.9 (8.4) | 28.9 (20.1) | **72.7 (3.7)** | 86.1 (5.8) | 23.3 (20.1) | **71.6 (4.1)** |
| $\boldsymbol{\upsilon}$**-**$\boldsymbol{SVM}$ | 91.7 (11.4) | 18.2 (8.6) | **61.4 (7.5)** | 93.0 (6.8) | 19.0 (8.2) | 65.7 (5.7) | 96.5 (5.1) | 26.4 (11.9) | 69.1 (5.7) | 96.5 (3.8) | 27.6 (23.2) | 69.7 (9.5) |
| Nearest Neighbour | 39.4 (11.8) | 16.7 (0.0) | 54.1 (1.4) | 32.8 (14.9) | 6.7 (11.1) | 66.2 (3.2) | 38.3 (9.5) | 8.6 (9.4) | 66.4 (0.7) | 29.7 (9.5) | 4.5 (13.7) | 66.5 (0.5) |
| MST | 45.3 (12.7) | 16.7 (0.0) | 53.8 (5.1) | 42.1 (16.2) | 18.3 (5.0) | 69.6 (4.2) | 52.1 (10.6) | 23.3 (3.3) | 70.8 (3.0) | 52 (11.7) | 18.3 (5.0) | 71.4 (1.3) |
|  | **Density-Based Method** | | | | | | | | | | | |
| Gaussian | 90.0 (15.9) | 18.3 (5.0) | 54.2 (3.9) | 93.9 (8.2) | 23.3 (20.1) | 70.3 (2.8) | 96.2 (5.6) | 28.9(20.1) | 73.2 (3.1) | 97.4 (3.9) | 23.3 (20.1) | 72.0 (3.8) |
| MoG | 81.6 (19.7) | 30.7 (10.6) | 52.2 (10.1) | 89.0 (7.9) | 23.2 (19.4) | 69.4 (3.8) | 93.0 (5.0) | 28.9 (19.7) | 71.5 (4.4) | 94.8 (3.7) | 23.6 (17.6) | 70.7 (3.7) |
| MCD Gaussian | 89.4 (13.0) | 16.7 (0.0) | **53.8 (5.1)** | 93.1 (8.0) | 21.7 (15.0) | 70.5 (1.6) | 97.1 (5.0) | 28.9 (20.1) | 73.2 (3.1) | 97.5 (4.0) | 25.0 (20.1) | 72.5 (3.9) |
| Parzen | 87.2 (16.3) | 21.7 (10.7) | **50.9 (6.8)** | 90.9 (8.1) | 23.3 (20.1) | 70.3 (2.8) | 94.2 (5.8) | 32.2 (21.4) | 73.6 (4.9) | 95.5 (4.3) | 26.7 (21.4) | 71.7 (4.2) |
| Naive Parzen | 88.9 (11.9) | 20.0 (6.7) | **53.2 (4.9)** | 91.2 (8.2) | 23.3 (20.1) | **71.1 (1.1)** | 95.4 (5.7) | 33.3 (21.1) | **74.0 (3.4)** | 95.9 (4.3) | 38.3 (16.8) | **76.0 (3.4)** |
| k-NN | 92.2 (10.6) | 20.0 (10.0) | **54.8 (2.6)** | 93.9 (6.7) | 23.3 (20.1) | **71.1 (1.1)** | 97.1 (5.3) | 28.9 (20.1) | 73.2 (3.1) | 97.4 (3.9) | 23.3 (20.1) | 72.0 (3.8) |
| LOF | 91.1 (10.9) | 16.7 (0.0) | 53.8 (5.1) | 94.5 (8.4) | 23.3 (20.1) | 70.3 (2.8) | 95.9 (5.5) | 28.9 (20.1) | 73.2 (3.1) | 97.2 (4.0) | 23.3 (20.1) | 72.0 (3.8) |
|  | **Reconstruction-Based Method** | | | | | | | | | | | |
| PCA | 71.0 (9.6) | 18.3 (5.0) | 52.3 (9.4) | 74.2 (9.5) | 16.7 (0.0) | 70.1 (2.7) | 79.0 (7.7) | 23.3 (3.3) | 71.4 (1.9) | 77.8 (6.0) | 16.7 (0.0) | 70.4 (1.3) |
| Auto – encoder | 70.2 (19.2) | 25.3 (10.8) | 54.3 (7.8) | 73.4 (16.3) | 21.8 (14.5) | 70.2 (3.8) | 81.0 (13.0) | 28.3 (15.0) | 72.1 (3.3) | 84 (13.0) | 22.3 (13.9) | 71.2 (3.1) |
| SOM | 61.5 (19.9) | 24.0 (8.3) | 54.2 (6.6) | 67.3 (10.4) | 21.8 (13.2) | 67.8 (4.5) | 86.2 (8.3) | 27.3 (13.1) | 71.7 (2.2) | 94.1 (4.8) | 17.7 (6.7) | 70.1 (2.1) |
| K-means | 93.3 (9.6) | 18.3 (5.0) | **54.2 (3.9)** | 92.5 (6.3) | 23.3 (20.1) | **71.1 (1.1)** | 96.2 (5.5) | 28.9 (20.1) | **73.2 (3.1)** | 96.0 (4.1) | 23.3 (20.1) | **72.0 (3.8)** |

**Table 2 (b)**: Average and standard deviation of AUC, specificity, F1-score for smoothed version of the data with a two-days moving average filter and different sample size.

| **Fraction = 0.01** | | | | | | | | | | | | |
| --- | --- | --- | --- | --- | --- | --- | --- | --- | --- | --- | --- | --- |
| Models | **Boundary and Domain-Based Method** | | | | | | | | | | | |
|  | 1 Month | | | 2 Months | | | 3 Months | | | 4 Months | | |
|  | **AUC** | **Specificity** | **F1** | **AUC** | **Specificity** | **F1** | **AUC** | **Specificity** | **F1** | **AUC** | **Specificity** | **F1** |
| SVDD | 100 (0.0) | 100 (0.0) | 96.0 (8.0) | 100 (0.0) | 100 (0.0) | 98.2 (3.6) | 100 (0.0) | 100 (0.0) | 96.0 (8.5) | 100 (0.0) | 100 (0.0) | 98.7 (2.0) |
| incsvdd | 100 (0.0) | 100 (0.0) | 96.0 (8.0) | 100 (0.0) | 100 (0.0) | 98.2 (3.6) | 100 (0.0) | 100 (0.0) | 96.6 (8.6) | 100 (0.0) | 100 (0.0) | 98.7 (2.0) |
| $\boldsymbol{\upsilon}$**-**$\boldsymbol{SVM}$ | 100 (0.0) | 100 (0.0) | **99.1 (3.1)** | 100 (0.0) | 100 (0.0) | **99.2 (2.3)** | 100 (0.0) | 100 (0.0) | **99.5 (1.6)** | 100 (0.0) | 100 (0.0) | **99.5 (1.3)** |
| Nearest Neighbour | 76.7 (21.3) | 13.3 (25.7) | 55.7 (10.6) | 79 (12.0) | 25.0 (26.2) | 72.4 (9.8) | 77 (11.4) | 17 (13.4) | 70.5 (4.3) | 79.7 (8.5) | 20 (27.8) | 71.7 (8.2) |
| MST | 97.2 (6.7) | 55 (10.7) | 68.3 (8.5) | 99.4 (1.1) | 86.7 (6.7) | 92.1 (0.7) | 100 (0.0) | 97.8 (6.7) | 97.2 (4.6) | 99.7 (0.6) | 100 (0.0) | 98.7 (2.9) |
|  | **Density-Based Method** | | | | | | | | | | | |
| Gaussian | 96.7 (10.0) | 72 (16.8) | 74.9 (12.4) | 98.1 (5.0) | 87 (14.6) | 91.3 (9.2) | 100 (0.0) | 100 (0.0) | 98.8 (2.4) | 100 (0.0) | 100 (0.0) | **99.6 (1.3)** |
| MoG | 96.7 (10.0) | 72 (16.8) | 74.9 (12.4) | 98.1 (5.0) | 88 (10.7) | 91.9 (8.6) | 100 (0.0) | 100 (0.0) | 98.2 (4.0) | 100 (0.0) | 100 (0.0) | **99.6 (1.3)** |
| MCD Gaussian | 95.0 (10.7) | 55 (15.0) | 65.6 (5.5) | 97.2 (5.3) | 60 (17.0) | 82.0 (8.5) | 100 (0.0) | 100 (0.0) | **99.4 (1.8)** | 100 (0.0) | 100 (0.0) | **99.6 (1.3)** |
| Parzen | 97.2 (6.7) | 100 (0.0) | 93.0 (15.6) | 99.7 (0.8) | 100 (0.0) | 96.2 (6.5) | 100 (0.0) | 100 (0.0) | 94.1 (8.6) | 99.9 (0.4) | 100 (0.0) | 96.4 (4.5) |
| Naive Parzen | 100.0 (0.0) | 100 (0.0) | 95.0 (15.0) | 100 (0.0) | 100 (0.0) | 98.2 (3.6) | 100 (0.0) | 100 (0.0) | 97.6 (4.1) | 100 (0.0) | 100 (0.0) | 98.1 (4.3) |
| k-NN | 100.0 (0.0) | 98.3 (5.0) | **96.6 (7.0)** | 99.7 (0.8) | 100 (0.0) | **99.1 (2.7)** | 100 (0.0) | 100 (0.0) | 98.8 (3.8) | 100 (0.0) | 100 (0.0) | **99.6 (1.3)** |
| LOF | 100.0 (0.0) | 98.3 (5.0) | **96.6 (7.0)** | 99.4 (1.1) | 96.7 (6.7) | 96.7 (5.6) | 99.9 (0.4) | 94.4 (5.6) | 96.2 (3.5) | 100 (0.0) | 100 (0.0) | 99.1 (1.7) |
|  | **Reconstruction-Based Method** | | | | | | | | | | | |
| PCA | 76.5 (13.0) | 43.3 (13.4) | 60.8 (10.0) | 66.3 (8.3) | 26.7 (13.4) | 71.9 (7.1) | 56.9 (11.2) | 22.2 (12.2) | 70.7 (5.1) | 65.4 (6.8) | 25 (13.5) | 71.8 (4.6) |
| Auto – encoder | 93.6 (11.6) | 83.2 (19.6) | 81.8 (17.5) | 96.5 (7.2) | 86.2 (19.3) | 89.9 (10.4) | 97.4 (5.9) | 86.7 (19.4) | 91.1 (9.5) | 98.2 (4.3) | 89.4 (16.6) | 93.7 (7.1) |
| SOM | 97.1 (6.1) | 100 (0.0) | 82.1 (19.6) | 99.7 (1.2) | 100 (0.0) | 90.4 (13.2) | 100 (0.0) | 100 (0.0) | 97.3 (4.1) | 99.9 (0.4) | 100 (0.0) | 97.4 (4.6) |
| K-means | 100 (0.0) | 100 (0.0) | **98.0 (6.0)** | 100 (0.0) | 100 (0.0) | **99.1 (2.7)** | 100 (0.0) | 100 (0.0) | **98.0 (6.0)** | 100 (0.0) | 100 (0.0) | **99.6 (1.3)** |

1. The Third Case of Infection (Flu)

**Table 3 (a)**: Average and standard deviation of AUC, specificity, F1-score for the raw dataset (without smoothing) and different sample size.

| **Fraction = 0.01** | | | | | | | | | | | | |
| --- | --- | --- | --- | --- | --- | --- | --- | --- | --- | --- | --- | --- |
| Models | **Boundary and Domain-Based Method** | | | | | | | | | | | |
|  | 1 Month | | | 2 Months | | | 3 Months | | | 4 Months | | |
|  | **AUC** | **Specificity** | **F1** | **AUC** | **Specificity** | **F1** | **AUC** | **Specificity** | **F1** | **AUC** | **Specificity** | **F1** |
| SVDD | 79.7 (6.8) | 66.7 (0.0) | 78.2 (14.2) | 86.4 (4.7) | 66.7 (0.0) | 90.6 (3.7) | 84.6 (4.4) | 66.7 (0.0) | 88.3 (3.9) | 85.4 (4.7) | 66.7 (0.0) | 90.5 (4.3) |
| incsvdd | 83.1 (9.2) | 66.7 (0.0) | 79.6 (14.5) | 85.9 (4.7) | 66.7 (0.0) | 90.6 (3.7) | 84.9 (4.0) | 66.7 (0.0) | 88.3 (3.9) | 85.6 (4.9) | 66.7 (0.0) | 90.5 (4.3) |
| $\boldsymbol{\upsilon}$**-**$\boldsymbol{SVM}$ | 83.1 (7.8) | 66.7 (0.0) | 83.7 (5.8) | 82.9 (5.1) | 66.7 (0.0) | 86.3 (4.2) | 84.1 (4.8) | 66.7 (0.0) | 86.0 (2.6) | 84.7 (5.9) | 66.7 (0.0) | 87.1 (2.4) |
| Nearest Neighbour | 89.4 (11.8) | 70.0 (10.0) | 85.6 (7.6) | 96.7 (5.1) | 73.3 (13.4) | **92.1 (3.8)** | 81.2 (13.7) | 3.3 (10.0) | 75.9 (2.1) | 79.3 (9.6) | 3.3 (10.0) | 80.3 (0.6) |
| MST | 95.6 (7.4) | 66.7 (0.0) | **84.1 (5.9)** | 94.0 (3.7) | 66.7 (0.0) | 90.5 (5.9) | 89.0 (4.7) | 66.7 (0.0) | **89.5 (1.8)** | 92.0 (4.1) | 66.7 (0.0) | **91.5 (1.8)** |
|  | **Density-Based Method** | | | | | | | | | | | |
| Gaussian | 87.2 (7.9) | 66.7 (0.0) | **82.2 (7.9)** | 87.5 (6.7) | 66.7 (0.0) | 89.7 (4.2) | 87.7 (5.7) | 66.7 (0.0) | 88.9 (3.7) | 88.2 (4.3) | 66.7 (0.0) | 90.5 (4.3) |
| MoG | 90.6 (8.8) | 68.7 (7.9) | 76.3 (14.8) | 85.6 (5.1) | 66.7 (0.0) | 87.9 (5.9) | 88.6 (5.5) | 66.7 (0.0) | 88.2 (4.0) | 90.5 (3.8) | 66.7 (0.0) | 90.7 (3.9) |
| MCD Gaussian | 87.2 (7.9) | 66.7 (0.0) | **82.2 (7.9)** | 87.5 (6.7) | 66.7 (0.0) | 90.6 (3.7) | 88.0 (5.9) | 66.7 (0.0) | 88.9 (3.7) | 88.2 (4.3) | 66.7 (0.0) | 91.0 (2.9**)** |
| Parzen | 86.4 (8.7) | 66.7 (0.0) | 77.7 (14.8) | 85.4 (6.4) | 66.7 (0.0) | 87.8 (6.5) | 85.8 (7.1) | 66.7 (0.0) | 86.0 (4.0) | 87.4 (5.0) | 66.7 (0.0) | 90.6 (3.0) |
| Naive Parzen | 79.7 (6.8) | 66.7 (0.0) | 77.7 (14.8) | 80.3 (3.6) | 66.7 (0.0) | 87.9 (6.3) | 77.7 (6.3) | 66.7 (0.0) | 88.9 (2.4) | 82.2 (6.2) | 66.7 (0.0) | 91.0 (4.2) |
| k-NN | 81.9 (7.6) | 66.7 (0.0) | 81.5 (13.9) | 82.1 (4.4) | 66.7 (0.0) | 90.6 (3.7) | 86.2 (7.0) | 66.7 (0.0) | 88.9 (2.4) | 87.9 (5.0) | 66.7 (0.0) | 91.0 (4.2) |
| LOF | 81.1 (11.2) | 66.7 (0.0) | 81.5 (13.9) | 82.1 (6.1) | 66.7 (0.0) | **91.5 (2.8)** | 84.7 (6.7) | 66.7 (0.0) | **89.5 (1.8)** | 86.3 (4.9) | 66.7 (0.0) | **91.9 (1.3)** |
|  | **Reconstruction-Based Method** | | | | | | | | | | | |
| PCA | 92.5 (7.1) | 66.7 (0.0) | **84.1 (5.9)** | 94.1 (4.8) | 66.7 (0.0) | **89.7 (4.2)** | 93.4 (2.6) | 66.7 (0.0) | **89.5 (1.8)** | 93.4 (3.5) | 66.7 (0.0) | **91.0 (2.9)** |
| Auto – encoder | 86.4 (13.3) | 68.8 (13.8) | 81.0 (13.3) | 84.2 (10.0) | 65.2 (8.4) | 89.1 (6.2) | 83.3 (10.7) | 60 (16.0) | 86.5 (5.3) | 85.4 (9.6) | 59.7 (16.2) | 89.7 (4.4) |
| SOM | 94.7 (7.8) | 66.8 (2.4) | 80.5 (11.9) | 93.6 (4.8) | 67.5 (5.2) | 88.4 (6.3) | 93.4 (4.8) | 66.7 (0.0) | 87.7 (3.7) | 94.2 (3.9) | 66.7 (0.0) | **91.3 (2.6)** |
| K-means | 94.6 (8.1) | 70.5 (10.7) | 82.2 (10.7) | 90.4 (8.8) | 67.0 (3.3) | 88.5 (6.7) | 92.2 (5.8) | 66.7 (0.0) | 88.5 (3.0) | 94.4 (5.0) | 66.7 (0.0) | **91.1 (2.8)** |

**Table 3 (b)**: Average and standard deviation of AUC, specificity, F1-score for smoothed version of the data with a two-days moving average filter and different sample size.

| **Fraction = 0.01** | | | | | | | | | | | | |
| --- | --- | --- | --- | --- | --- | --- | --- | --- | --- | --- | --- | --- |
| Models | **Boundary and Domain-Based Method** | | | | | | | | | | | |
|  | 1 Month | | | 2 Months | | | 3 Months | | | 4 Months | | |
|  | **AUC** | **Specificity** | **F1** | **AUC** | **Specificity** | **F1** | **AUC** | **Specificity** | **F1** | **AUC** | **Specificity** | **F1** |
| SVDD | 100 (0.0) | 100 (0.0) | 93.6 (15.2) | 100 (0.0) | 100 (0.0) | 96.2 (6.5) | 100 (0.0) | 100 (0.0) | 96.8 (6.1) | 100 (0.0) | 100 (0.0) | 98.1 (4.3) |
| incsvdd | 100 (0.0) | 100 (0.0) | 93.6 (15.2) | 100 (0.0) | 100 (0.0) | 96.2 (6.5) | 100 (0.0) | 100 (0.0) | 96.8 (6.1) | 100 (0.0) | 100 (0.0) | 98.1 (4.3) |
| $\boldsymbol{\upsilon}$**-**$\boldsymbol{SVM}$ | 100 (0.0) | 100 (0.0) | **99.6 (2.5)** | 100 (0.0) | 100 (0.0) | **98.9 (3.5)** | 100 (0.0) | 100 (0.0) | **99.3 (2.2)** | 100 (0.0) | 100 (0.0) | **99.4 (1.9)** |
| Nearest Neighbour | 95.6 (13.4) | 73.3 (41.7) | 84.3 (18.7) | 96.2 (3.5) | 43.3 (21.4) | 87.9 (4.6) | 93.7 (7.8) | 3.3 (10.0) | 74.8 (3.3) | 92.5 (8.3) | 3.3 (10.0) | 79.8 (3.3) |
| MST | 100 (0.0) | 100 (0.0) | 95.0 (15.0) | 100 (0.0) | 100 (0.0) | 95.1 (8.0) | 100 (0.0) | 100 (0.0) | 98.2 (2.7) | 100 (0.0) | 100 (0.0) | **99.1 (1.7)** |
|  | **Density-Based Method** | | | | | | | | | | | |
| Gaussian | 100 (0.0) | 100 (0.0) | 93.0 (15.6) | 100 (0.0) | 100 (0.0) | 96.0 (8.0) | 100 (0.0) | 100 (0.0) | 98.0 (6.0) | 100 (0.0) | 100 (0.0) | **99.1 (2.7)** |
| MoG | 100 (0.0) | 100 (0.0) | **92.8 (12.9)** | 100 (0.0) | 100 (0.0) | 92.5 (13.0) | 100 (0.0) | 100 (0.0) | 96.0 (6.6) | 100 (0.0) | 100 (0.0) | 97.5 (4.4) |
| MCD Gaussian | 100 (0.0) | 100 (0.0) | 95.0 (15.0) | 100 (0.0) | 100 (0.0) | 97.1 (6.3) | 100 (0.0) | 100 (0.0) | 98.0 (6.0) | 100 (0.0) | 100 (0.0) | **99.1 (2.7)** |
| Parzen | 100 (0.0) | 100 (0.0) | 91.6 (15.5) | 100 (0.0) | 100 (0.0) | 93.1 (9.0) | 100 (0.0) | 100 (0.0) | 92.9 (8.5) | 100 (0.0) | 100 (0.0) | 97.3 (4.3) |
| Naive Parzen | 100 (0.0) | 100 (0.0) | 95.0 (15.0) | 100 (0.0) | 100 (0.0) | 97.1 (6.3) | 100 (0.0) | 100 (0.0) | 98.0 (6.0) | 100 (0.0) | 100 (0.0) | 98.6 (4.3) |
| k-NN | 100 (0.0) | 100 (0.0) | 95.0 (15.0) | 100 (0.0) | 100 (0.0) | **98.0 (6.0)** | 100 (0.0) | 100 (0.0) | 98.0 (6.0) | 100 (0.0) | 100 (0.0) | **99.1 (2.7)** |
| LOF | 100 (0.0) | 100 (0.0) | 95.0 (15.0) | 100 (0.0) | 100 (0.0) | **98.0 (6.0)** | 100 (0.0) | 100 (0.0) | **98.8 (2.4)** | 100 (0.0) | 100 (0.0) | **99.1 (2.7)** |
|  | **Reconstruction-Based Method** | | | | | | | | | | | |
| PCA | 100 (0.0) | 100 (0.0) | **96.0 (8.0)** | 100 (0.0) | 100 (0.0) | **99.1 (2.7)** | 100 (0.0) | 100 (0.0) | **99.4 (1.8)** | 100 (0.0) | 96.7 (10.0) | 97.9 (2.7) |
| Auto – encoder | 99.2 (4.9) | 99.5 (4.1) | 93.2 (14.5) | 98.5 (5.3) | 94.3 (15.4) | 93.7 (9.9) | 98.8 (5.2) | 94.3 (15.7) | 95.5 (7.3) | 99.1 (3.8) | 93.7 (16.8) | 97.0 (4.8) |
| SOM | 100 (0.0) | 100 (0.0) | 90.3 (17.8) | 100 (0.0) | 100 (0.0) | 91.3 (13.5) | 100 (0.0) | 100 (0.0) | 93.5 (9.3) | 100 (0.0) | 100 (0.0) | 97.3 (3.8) |
| K-means | 100 (0.0) | 100 (0.0) | 95.0 (15.0) | 100 (0.0) | 100 (0.0) | 98.0 (6.0) | 100 (0.0) | 100 (0.0) | 98.0 (6.0) | 100 (0.0) | 100 (0.0) | **99.1 (2.7)** |

1. The Fourth Case of Infection (Flu)

**Table 4 (a)**: Average and standard deviation of AUC, specificity, F1-score for the raw dataset (without smoothing) and different sample size.

| **Fraction = 0.01** | | | | | | | | | | | | |
| --- | --- | --- | --- | --- | --- | --- | --- | --- | --- | --- | --- | --- |
| Models | **Boundary and Domain-Based Method** | | | | | | | | | | | |
|  | 1 Month | | | 2 Months | | | 3 Months | | | 4 Months | | |
|  | **AUC** | **Specificity** | **F1** | **AUC** | **Specificity** | **F1** | **AUC** | **Specificity** | **F1** | **AUC** | **Specificity** | **F1** |
| SVDD | 100 (0.0) | 100 (0.0) | **94.0 (9.2)** | 97.1 (3.3) | 75.0 (0.0) | 88.8 (4.5) | 98.7 (1.4) | 85.7 (0.0) | 92.5 (2.8) | 98.4 (1.8) | 75.0 (0.0) | 90.6 (2.9) |
| incsvdd | 100 (0.0) | 100 (0.0) | **94.0 (9.2)** | 96.7 (4.1) | 75.0 (0.0) | **89.7 (4.2)** | 98.6 (1.3) | 85.7 (0.0) | **93.0 (2.7)** | 97.9 (2.1) | 75.0 (0.0) | **91.0 (2.9)** |
| $\boldsymbol{\upsilon}$**-**$\boldsymbol{SVM}$ | 97.9 (3.6) | 75.0 (0.0) | 85.7 (5.4) | 96.5 (4.7) | 74.8 (2.5) | 88.6 (3.8) | 98.4 (2.0) | 85.7 (0.0) | **93.2 (2.1)** | 97.6 (3.0) | 75.0 (0.0) | 89.5 (1.8) |
| Nearest Neighbour | 90.6 (13) | 5.9 (13.3) | 59.1 (5.3) | 69.5 (14.1) | 8.5 (10.4) | 74.4 (2.7) | 73.5 (12.4) | 20.0 (27.3) | 76.5 (8.1) | 78.1 (10.7) | 27.5 (26.2) | 81.1 (6.6) |
| MST | 99.2 (2.5) | 75.0 (0.0) | 84.1 (5.9) | 98.8 (2.7) | 55.0 (15.0) | 85.5 (3.3) | 98.2 (3.3) | 65.7 (17.2) | 87.5 (3.5) | 98.8 (2.1) | 60.0 (20.1) | 87.7 (4.1) |
|  | **Density-Based Method** | | | | | | | | | | | |
| Gaussian | 98.3 (3.3) | 82.5 (11.5) | 86.4 (9.0) | 97.5 (2.8) | 75.0 (0.0) | 90.6 (3.7) | 99.1 (1.0) | 85.7 (0.0) | **93.7 (2.1)** | 98.8 (1.7) | 75.0 (0.0) | 91.5 (1.6) |
| MoG | 99.1 (3.4) | 97.1 (8.0) | 88.8 (13.4) | 99.1 (2.1) | 90.5 (12.2) | 95.2 (4.8) | 98.7 (2.5) | 92.1 (7.1) | 94.4 (6.1) | 98.8 (2.7) | 87.4 (12.5) | 93.9 (6.0) |
| MCD Gaussian | 97.7 (3.6) | 77.5 (7.5) | **85.5 (2.1)** | 97.9 (2.8) | 75.0 (0.0) | 90.6 (3.7) | 99.1 (1.0) | 85.7 (0.0) | **93.7 (2.1)** | 99.0 (1.7) | 75.0 (0.0) | **92.0 (1.0)** |
| Parzen | 96.0 (4.0) | 80.0 (10.0) | 83.0 (6.0) | 95.8 (5.6) | 75.0 (0.0) | 89.7 (4.2) | 98.2 (2.6) | 87.1 (4.3) | 92.2 (6.0) | 99.0 (1.6) | 87.5 (12.5) | 93.0 (4.0) |
| Naive Parzen | 93.5 (6.2) | 75.0 (0.0) | 80.3 (9.0) | 93.3 (9.7) | 75.0 (0.0) | 88.7 (6.4) | 96.1 (6.3) | 77.1 (13.1) | 89.9 (7.0) | 96.3 (4.6) | 70.0 (10.0) | 89.8 (3.3) |
| k-NN | 98.3 (3.3) | 75.0 (0.0) | 84.1 (5.9) | 94.6 (4.6) | 72.5 (7.5) | 90.9 (3.2) | 98.0 (2.7) | 82.9 (8.6) | 92.7 (3.3) | 97.5 (3.1) | 75.0 (0.0) | 91.1 (2.8) |
| LOF | 96.0 (4.0) | 75.0 (0.0) | 84.1 (5.9) | 95.8 (4.9) | 75.0 (0.0) | **91.5 (2.8)** | 98.5 (2.1) | 82.9 (8.6) | 93.4 (2.9) | 98.6 (2.4) | 75.0 (0.0) | **92.0 (1.0)** |
|  | **Reconstruction-Based Method** | | | | | | | | | | | |
| PCA | 99.2 (2.5) | 100 (0.0) | **96.0 (8.0)** | 98.8 (1.9) | 85.0 (12.3) | **92.8 (5.8)** | 99.4 (1.0) | 88.6 (5.7) | 93.5 (3.5) | 99.4 (1.3) | 85.0 (12.3) | **94.1 (4.0)** |
| Auto – encoder | 95.7 (9.1) | 81.3 (25.4) | 83.7 (14.5) | 92.6 (11.7) | 63.9 (32.3) | 87.6 (9.0) | 95.9 (8.0) | 68.1 (33.9) | 88 (10.1) | 96.2 (9.2) | 66.0 (31.7) | 89.2 (8.1) |
| SOM | 99.2 (2.5) | 84.4 (12.1) | 89.4 (6.9) | 96.8 (3.8) | 74.9 (6.4) | 89.4 (4.3) | 98.6 (1.8) | 83.3 (8.0) | 92.7 (3.1) | 98.4 (2.6) | 75.0 (0.0) | 91.0 (2.3) |
| K-means | 96.9 (3.9) | 75.0 (0.0) | 84.1 (5.9) | 95.8 (4.9) | 75.0 (0.0) | 90.6 (3.7) | 98.0 (2.3) | 85.7 (0.0) | **94.2 (1.8)** | 96.7 (3.5) | 75.0 (0.0) | 91.5 (2.7) |

**Table 4 (b)**: Average and standard deviation of AUC, specificity, F1-score for smoothed version of the data with a two-days moving average filter and different sample size.

| **Fraction = 0.01** | | | | | | | | | | | | |
| --- | --- | --- | --- | --- | --- | --- | --- | --- | --- | --- | --- | --- |
| Models | **Boundary and Domain-Based Method** | | | | | | | | | | | |
|  | 1 Month | | | 2 Months | | | 3 Months | | | 4 Months | | |
|  | **AUC** | **Specificity** | **F1** | **AUC** | **Specificity** | **F1** | **AUC** | **Specificity** | **F1** | **AUC** | **Specificity** | **F1** |
| SVDD | 100 (0.0) | 100 (0.0) | 93 (15.6) | 100 (0.0) | 100 (0.0) | 95.1 (8.0) | 100 (0.0) | 100 (0.0) | 96.2 (6.0) | 100 (0.0) | 100 (0.0) | 97.7 (4.4) |
| incsvdd | 100 (0.0) | 100 (0.0) | 93.0 (15.6) | 100 (0.0) | 100 (0.0) | 96.0 (8.0) | 100 (0.0) | 100 (0.0) | 98.2 (2.7) | 100 (0.0) | 100 (0.0) | **99.1 (1.7)** |
| $\boldsymbol{\upsilon}$**-**$\boldsymbol{SVM}$ | 100 (0.0) | 100 (0.0) | **98.9 (3.7)** | 100 (0.0) | 100 (0.0) | **99.0 (3.0)** | 100 (0.0) | 100 (0.0) | **99.3 (2.1)** | 100 (0.0) | 100 (0.0) | **99.5 (1.7)** |
| Nearest Neighbour | 90.0 (12.3) | 15 (30.1) | 65.8 (10.2) | 93.3 (7.5) | 47.5 (17.5) | 82.8 (5.6) | 97.8 (2.6) | 60.0 (5.7) | 85.5 (2.3) | 97.5 (3.1) | 62.5 (12.5) | 87.8 (4.7) |
| MST | 100 (0.0) | 100 (0.0) | 98.0 (6.0) | 100 (0.0) | 100 (0.0) | 96.2 (6.5) | 100 (0.0) | 100 (0.0) | **99.4 (1.8)** | 100 (0.0) | 100 (0.0) | 97.1 (6.0) |
|  | **Density-Based Method** | | | | | | | | | | | |
| Gaussian | 100 (0.0) | 100 (0.0) | 89.0 (15.8) | 100 (0.0) | 100 (0.0) | 95.0 (15.0) | 100 (0.0) | 100 (0.0) | **98.0 (6.0)** | 100 (0.0) | 100 (0.0) | 97.4 (7.9) |
| MoG | 100 (0.0) | 100 (0.0) | 84.5 (22.6) | 100 (0.0) | 100 (0.0) | 91.8 (12.3) | 99.9 (0.6) | 100 (0.0) | 93.7 (8.2) | 100 (0.3) | 100 (0.0) | 95.7 (8.1) |
| MCD Gaussian | 100 (0.0) | 100 (0.0) | 95.0 (15.0) | 100 (0.0) | 100 (0.0) | 96.7 (10.0) | 100 (0.0) | 100 (0.0) | **98.0 (6.0)** | 100 (0.0) | 100 (0.0) | 97.4 (7.9) |
| Parzen | 100 (0.0) | 100 (0.0) | 91.0 (15.8) | 100 (0.0) | 100 (0.0) | 90.4 (13.4) | 100 (0.0) | 100 (0.0) | 92.9 (8.0) | 100 (0.0) | 100 (0.0) | 95.1 (8.0) |
| Naive Parzen | 100 (0.0) | 100 (0.0) | 93.0 (15.6) | 100 (0.0) | 100 (0.0) | 95.1 (8.0) | 100 (0.0) | 100 (0.0) | 96.8 (6.7) | 100 (0.0) | 100 (0.0) | 97.2 (4.8) |
| k-NN | 100 (0.0) | 100 (0.0) | 96.0 (8.0) | 100 (0.0) | 100 (0.0) | **98.2 (3.6)** | 100 (0.0) | 100 (0.0) | **98.0 (6.0)** | 100 (0.0) | 100 (0.0) | **98.6 (4.3)** |
| LOF | 100 (0.0) | 100 (0.0) | **98.0 (6.0)** | 100 (0.0) | 100 (0.0) | 95.8 (10.1) | 100 (0.0) | 100 (0.0) | **98.0 (6.0)** | 100 (0.0) | 100 (0.0) | 97.4 (7.9) |
|  | **Reconstruction-Based Method** | | | | | | | | | | | |
| PCA | 100 (0.0) | 100 (0.0) | **93.0 (15.6)** | 100 (0.0) | 100 (0.0) | 95.0 (15.0) | 99.4 (1.9) | 94.3 (11.5) | 96.0 (6.6) | 99.8 (0.6) | 100 (0.0) | 97.4 (7.9) |
| Auto – encoder | 97.9 (7.5) | 94.8 (15.6) | 90.1 (15.9) | 98.9 (4.8) | 95.4 (14.0) | 92.8 (12.1) | 99.0 (3.6) | 96.5 (10.6) | 95.5 (7.3) | 99.0 (3.8) | 93.5 (17.6) | 95.4 (7.7) |
| SOM | 100 (0.0) | 100 (0.0) | 83.8 (22.5) | 100 (0.0) | 100 (0.0) | 82.8 (23.3) | 100 (0.0) | 100 (0.0) | 92.4 (9.1) | 100 (0.0) | 100 (0.0) | 95.5 (8.3) |
| K-means | 100 (0.0) | 100 (0.0) | **93.0 (15.6)** | 100 (0.0) | 100 (0.0) | **97.1 (6.3)** | 100 (0.0) | 100 (0.0) | **97.5 (6.0)** | 100 (0.0) | 100 (0.0) | **98.2 (4.3)** |

## **Hourly**

1. The First Case of Infection (Flu)

**Table 5:** Average and standard deviation of AUC, specificity, F1-score for smoothed version of the data with 48 hours moving average filter and different sample size.

| **Fraction = 0.01** | | | | | | | | | | | | |
| --- | --- | --- | --- | --- | --- | --- | --- | --- | --- | --- | --- | --- |
| Models | **Boundary and Domain-Based Method** | | | | | | | | | | | |
|  | 1 Month | | | 2 Months | | | 3 Months | | | 4 Months | | |
|  | **AUC** | **Specificity** | **F1** | **AUC** | **Specificity** | **F1** | **AUC** | **Specificity** | **F1** | **AUC** | **Specificity** | **F1** |
| SVDD | 97.6 (1.9) | 83.2 (3.4) | 85.8 (1.7) | 97.8 (1.2) | 85.7 (5.0) | 90.5 (9.6) | 97.7 (1.2) | 90.4 (5.1) | 94.2 (2.9) | 98.1 (0.9) | 91.0 (3.7) | **96.8 (0.9)** |
| incsvdd | 97.4 (1.9) | 84.5 (2.8) | 86.8 (1.9) | 97.7 (1.2) | 86.7 (2.0) | 93.9 (1.0) | 97.5 (1.2) | 88.5 (1.5) | **96.0 (1.1)** | 97.9 (0.9) | 88.9 (1.2) | **97.0 (0.7)** |
| $\boldsymbol{\upsilon}$**-**$\boldsymbol{SVM}$ | 98.1 (2.1) | 84.5 (1.1) | **90.5 (1.1)** | 99.0 (1.1) | 92.6 (0.0) | **96.1 (1.3)** | 99.5 (0.6) | 93.8 (0.5) | **96.9 (1.4)** | 99.4 (0.4) | 94.2 (0.0) | **97.1 (1.3)** |
| Nearest Neighbour | 84.8 (6.0) | 75.9 (4.5) | 74.8 (6.0) | 89.3 (2.2) | 76.5 (4.1) | 87.1 (3.3) | 89.0 (4.0) | 77.5 (3.9) | 89.3 (4.4) | 90.2 (4.7) | 77.5 (3.8) | 91.4 (6.4) |
| MST | 90.5 (3.1) | 85.4 (3.9) | 67.6 (14.5) | 94.4 (2.0) | 85.7 (4.0) | 85.1 (7.0) | 94.7 (2.4) | 88.8 (3.5) | 87.8 (8.5) | 95.8 (2.2) | 88.8 (3.0) | 90.9 (5.9) |
|  | **Density-Based Method** | | | | | | | | | | | |
| Gaussian | 98.1 (2.2) | 79.8 (4.9) | 83.9 (2.7) | 99.5 (0.9) | 90.1 (1.7) | 95.2 (1.8) | 99.6 (0.7) | 92.9 (1.3) | **97.1 (2.5)** | 99.5 (0.5) | 92.2 (1.0) | 97.7 (1.1) |
| MoG | 95.8 (3.6) | 82.7 (4.3) | 83.7 (5.0) | 98.3 (1.5) | 86.2 (2.7) | 92.3 (2.7) | 98.7 (1.4) | 88.7 (4.6) | 94.7 (3.5) | 98.6 (1.6) | 88.2 (3.1) | 95.3 (3.2) |
| MCD Gaussian | 98.6 (2.1) | 75.3 (6.9) | 81.3 (2.5) | 99.6 (0.9) | 89.6 (1.9) | 95.0 (1.8) | 99.6 (0.7) | 92.5 (1.8) | **97.0 (2.3)** | 99.6 (0.4) | 92.0 (1.2) | 97.7 (1.1) |
| Parzen | 91.9 (2.9) | 93.6 (2.0) | 63.4 (16.5) | 96.2 (2.3) | 94.4 (2.0) | 81.6 (10.2) | 96.6 (2.6) | 94.8 (1.7) | 84.2 (9.5) | 97.4 (2.2) | 95.6 (1.2) | 87.9 (7.1) |
| Naive Parzen | 94.8 (3.7) | 76.4 (5.6) | 77.6 (7.9) | 98.7 (1.2) | 85.2 (3.3) | 91.8 (2.9) | 99.1 (1.1) | 89.1 (3.8) | 94.8 (2.5) | 98.9 (0.9) | 89.7 (2.4) | 96.2 (1.6) |
| k-NN | 97.1 (3.4) | 78.8 (2.0) | **84.2 (2.1)** | 99.1 (1.0) | 92.9 (0.7) | **96.0 (1.8)** | 99.6 (0.4) | 93.8 (0.7) | **97.3 (1.9)** | 99.5 (0.3) | 94.0 (0.6) | **98.2 (0.9)** |
| LOF | 96.9 (3.5) | 78.3 (3.0) | 84.2 (2.4) | 99.2 (1.1) | 91.9 (0.9) | **96.0 (1.8)** | 99.6 (0.5) | 93.7 (0.8) | **97.3 (2.1)** | 99.5 (0.4) | 93.1 (0.4) | 97.8 (1.2) |
|  | **Reconstruction-Based Method** | | | | | | | | | | | |
| PCA | 97.1 (3.4) | 63.9 (8.8) | 75.4 (0.3) | 99.4 (1.2) | 76.4 (6.6) | 90.2 (1.1) | 99.1 (1.3) | 75.1 (6.8) | 92.4 (1.1) | 98.9 (1.2) | 69.1 (4.1) | 93.1 (0.8) |
| Auto – encoder | 92.0 (4.8) | 79.5 (7.6) | 78.9 (8.3) | 96.2 (2.6) | 83.1 (7.2) | 91.1 (3.9) | 96.3 (3.2) | 84.3 (7.7) | 92.7 (5.0) | 96.7 (3.0) | 84.0 (8.0) | 94.6 (4.4) |
| SOM | 94.1 (2.3) | 82.2 (3.3) | 82.6 (4.9) | 95.6 (1.1) | 82.9 (3.1) | 91.6 (1.9) | 94.8 (2.3) | 83.4 (5.8) | 92.3 (4.1) | 95.5 (1.9) | 84.1 (3.8) | 94.3 (3.8) |
| K-means | 97.3 (3.2) | 80.9 (2.5) | **85.5 (2.5)** | 98.9 (1.1) | 92.6 (0.7) | **95.8 (1.8)** | 99.3 (0.6) | 92.9 (0.7) | **97.3 (1.4)** | 99.4 (0.4) | 94.1 (0.2) | **98.1 (1.1)** |

1. The Second Case of Infection (Flu)

**Table 6**: Average and standard deviation of AUC, specificity, F1-score for smoothed version of the data with 48 hours moving average filter and different sample size.

| **Fraction = 0.01** | | | | | | | | | | | | |
| --- | --- | --- | --- | --- | --- | --- | --- | --- | --- | --- | --- | --- |
| Models | **Boundary and Domain-Based Method** | | | | | | | | | | | |
|  | 1 Month | | | 2 Months | | | 3 Months | | | 4 Months | | |
|  | **AUC** | **Specificity** | **F1** | **AUC** | **Specificity** | **F1** | **AUC** | **Specificity** | **F1** | **AUC** | **Specificity** | **F1** |
| SVDD | 100 (0.0) | 100 (0.0) | 96.3 (7.7) | 100 (0.0) | 100 (0.0) | 96.9 (5.9) | 98.5 (3.0) | 76.9 (7.8) | 91.9 (2.6) | 98.8 (2.2) | 81.0 (7.2) | 94.4 (2.4) |
| incsvdd | 100 (0.0) | 100 (0.0) | 98.1 (4.4) | 100 (0.0) | 100 (0.0) | 97.4 (5.3) | 98.6 (3.3) | 65.7 (11.6) | 89.4 (2.1) | 99.0 (2.2) | 74.3 (8.6) | 93.6 (1.7) |
| $\boldsymbol{\upsilon}$**-**$\boldsymbol{SVM}$ | 100 (0.0) | 100 (0.0) | **99.6 (0.9)** | 100 (0.0) | 100 (0.0) | **99.5 (1.6)** | 99.7 (0.8) | 98.7 (0.4) | **98.8 (1.9)** | 99.8 (0.6) | 99.6 (0.4) | **99.1 (2.0)** |
| Nearest Neighbour | 97.6 (4.9) | 100 (0.0) | 85.9 (14.1) | 99.4 (0.8) | 100 (0.0) | 94.9 (4.1) | 97.6 (1.1) | 92.5 (3.4) | 94.3 (2.2) | 98.1 (0.9) | 92.1 (3.2) | 95.8 (2.5) |
| MST | 100 (0.0) | 100 (0.0) | 78.6 (16.1) | 100 (0.0) | 100 (0.0) | 87.1 (8.6) | 97.8 (1.5) | 99.3 (0.2) | 91.6 (6.1) | 98.4 (1.3) | 99.3 (0.2) | 93.0 (4.2) |
|  | **Density-Based Method** | | | | | | | | | | | |
| Gaussian | 100 (0.0) | 100 (0.0) | **97.5 (7.6)** | 100 (0.0) | 100 (0.0) | 97.8 (4.7) | 99.0 (1.6) | 79.3 (7.6) | 92.8 (1.8) | 99.6 (0.7) | 84.9 (5.8) | 95.6 (1.9) |
| MoG | 100 (0.0) | 99.4 (1.7) | 93.8 (10.9) | 100 (0.0) | 100 (0.0) | 96.7 (6.1) | 98.0 (2.8) | 78.2 (10.5) | 91.3 (3.2) | 98.7 (1.9) | 82.0 (8.7) | 94.1 (3.2) |
| MCD Gaussian | 100 (0.0) | 100 (0.0) | **97.5 (7.6)** | 100 (0.0) | 100 (0.0) | **98.1 (4.1)** | 99.2 (1.3) | 78.2 (6.1) | 92.8 (1.9) | 99.6 (0.6) | 86.1 (6.5) | 95.9 (1.8) |
| Parzen | 100 (0.0) | 100 (0.0) | 58.9 (16.4) | 100 (0.0) | 100 (0.0) | 79.8 (13.1) | 98.3 (1.7) | 99.3 (0.2) | 84.4 (10.2) | 98.9 (1.3) | 99.3 (0.2) | 87.8 (6.6) |
| Naive Parzen | 100 (0.0) | 100 (0.0) | 94.8 (10.2) | 100 (0.0) | 100 (0.0) | 95.8 (8.0) | 99.0 (2.4) | 95.0 (3.1) | **96.2 (4.0)** | 98.9 (1.9) | 98.0 (1.5) | **97.1 (3.2)** |
| k-NN | 100 (0.0) | 100 (0.0) | **97.5 (7.6)** | 100 (0.0) | 100 (0.0) | 95.8 (7.6) | 98.7 (1.4) | 95.5 (2.3) | 95.1 (4.0) | 99.2 (1.1) | 94.6 (2.3) | 96.1 (3.5) |
| LOF | 100 (0.0) | 100 (0.0) | 94.1 (11.2) | 100 (0.0) | 100 (0.0) | 95.6 (3.8) | 90.0 (4.9) | 77.9 (6.9) | 88.7 (5.6) | 91.8 (2.2) | 79.1 (6.3) | 91.4 (3.4) |
|  | **Reconstruction-Based Method** | | | | | | | | | | | |
| PCA | 95.9 (4.4) | 65.5 (6.4) | 76.5 (1.3) | 91.7 (7.3) | 54.2 (4.7) | 82.5 (4.5) | 74.4 (8.1) | 33.7 (2.9) | 82.8 (2.8) | 77.1 (6.1) | 32.1 (2.2) | 86.7 (1.0) |
| Auto – encoder | 99.5 (1.8) | 97.1 (9.2) | 92.5 (10.8) | 99.4 (2.3) | 97.7 (7.0) | 95.7 (6.3) | 93.4 (7.9) | 69.4 (19.7) | 90.0 (5.2) | 95.1 (5.9) | 71 (20.9) | 92.6 (4.1) |
| SOM | 100 (0.0) | 100 (0.0) | 94.7 (9.0) | 100 (0.0) | 100 (0.0) | 96.2 (5.4) | 93.8 (5.5) | 69.0 (18.9) | 88.9 (5.9) | 99.1 (2.3) | 95.5 (10.9) | 97.4 (3.7) |
| K-means | 100 (0.0) | 100 (0.0) | **99.3 (1.8)** | 100 (0.0) | 100 (0.0) | **98.0 (4.3)** | 99.7 (0.8) | 98.4 (0.6) | **98.5 (3.2)** | 99.8 (0.6) | 99.3 (0.3) | **99.0 (2.4)** |

1. The Third Case of Infection (Flu)

**Table 7**: Average and standard deviation of AUC, specificity, F1-score for smoothed version of the data with 48 hours moving average filter and different sample size.

| **Fraction = 0.01** | | | | | | | | | | | | |
| --- | --- | --- | --- | --- | --- | --- | --- | --- | --- | --- | --- | --- |
| Models | **Boundary and Domain-Based Method** | | | | | | | | | | | |
|  | 1 Month | | | 2 Months | | | 3 Months | | | 4 Months | | |
|  | **AUC** | **Specificity** | **F1** | **AUC** | **Specificity** | **F1** | **AUC** | **Specificity** | **F1** | **AUC** | **Specificity** | **F1** |
| SVDD | 93.5 (5.3) | 79.8 (5.3) | 84.8 (8.2) | 96.3 (2.4) | 77.7 (4.2) | 91.8 (4.6) | 95.9 (3.6) | 69.8 (4.3) | **93.3 (3.0)** | 96.3 (2.3) | 68.9 (3.0) | 95.1 (1.3) |
| incsvdd | 92.8 (5.2) | 74.4 (3.6) | 82.8 (7.3) | 95.7 (2.4) | 76.7 (4.1) | 91.4 (4.5) | 95.2 (3.4) | 67.4 (3.1) | **93.0 (2.3)** | 95.5 (2.2) | 69.6 (2.4) | 94.9 (1.5) |
| $\boldsymbol{\upsilon}$**-**$\boldsymbol{SVM}$ | 94.7 (3.3) | 64.1 (0.4) | **83.2 (1.7)** | 97.7 (2.1) | 80.2 (0.3) | **91.7 (1.6)** | 96.5 (2.4) | 67.4 (0.6) | 87.8 (2.3) | 97.4 (1.5) | 75.3 (0.2) | 90.8 (1.9) |
| Nearest Neighbour | 91.8 (12.7) | 92.1 (1.4) | 87.0 (8.8) | 96.2 (1.3) | 92.1 (0.8) | 92.9 (5.8) | 92.9 (3.1) | 81.6 (3.8) | 92.4 (5.6) | 93.6 (2.3) | 81.5 (3.4) | 94.9 (2.8) |
| MST | 94.3 (4.1) | 92.2 (1.9) | 74.6 (22.3) | 97.0 (1.8) | 94.2 (0.8) | 85.9 (14.8) | 94.0 (3.0) | 89.4 (1.1) | 91.1 (6.5) | 95.6 (1.3) | 89.4 (1.1) | **93.7 (4.2)** |
|  | **Density-Based Method** | | | | | | | | | | | |
| Gaussian | 96.0 (2.1) | 88.3 (1.1) | 86.0 (18.6) | 98.3 (1.5) | 89.4 (1.5) | **94.8 (6.5)** | 97.1 (1.7) | 87.0 (0.6) | **96.5 (1.8)** | 97.9 (1.2) | 88.0 (0.6) | **97.7 (1.0)** |
| MoG | 95.0 (4.5) | 85.1 (4.3) | 84.1 (16.2) | 98.1 (1.6) | 88.2 (2.5) | 93.1 (7.9) | 96.6 (2.5) | 82.1 (5.5) | 94.7 (4.1) | 97.2 (1.7) | 87.1 (1.6) | 96.6 (2.5) |
| MCD Gaussian | 95.9 (2.1) | 88.1 (1.3) | 85.9 (18.6) | 98.7 (1.4) | 89.4 (1.5) | **94.9 (6.2)** | 97.0 (1.6) | 86.9 (0.6) | **96.5 (1.7)** | 98.0 (1.2) | 88.1 (0.6) | **97.8 (0.9)** |
| Parzen | 94.1 (4.2) | 97.8 (0.5) | 58.8 (24.5) | 97.2 (1.9) | 96.5 (0.5) | 78.0 (20.0) | 95.1 (3.2) | 91.6 (1.7) | 86.2 (9.3) | 96.7 (1.8) | 92.0 (1.7) | 89.4 (7.1) |
| Naive Parzen | 93.4 (4.2) | 80.5 (3.4) | 83.9 (10.9) | 97.3 (2.0) | 81.2 (4.2) | 90.5 (8.9) | 95.2 (3.4) | 80.1 (0.4) | 93.8 (4.2) | 95.9 (2.3) | 80.2 (0.0) | 94.9 (2.5) |
| k-NN | 94.0 (4.5) | 85.7 (2.4) | 84.1 (17.7) | 97.2 (2.4) | 88.0 (2.7) | 92.5 (7.7) | 95.7 (2.4) | 85.4 (0.5) | 93.9 (6.4) | 96.5 (1.8) | 85.6 (0.6) | 96.7 (2.6) |
| LOF | 91.9 (4.3) | 86.7 (1.2) | **80.9 (11.2)** | 93.5 (2.8) | 84.0 (7.1) | 90.9 (4.9) | 92.1 (3.0) | 84.4 (2.3) | 92.3 (5.4) | 93.8 (2.2) | 83.7 (4.5) | 95.4 (3.3) |
|  | **Reconstruction-Based Method** | | | | | | | | | | | |
| PCA | 97.5 (2.6) | 87.5 (1.4) | **88.4 (14.5)** | 98.6 (1.2) | 89.1 (1.2) | **94.5 (5.2)** | 97.2 (1.4) | 85.1 (0.9) | **96.7 (1.5)** | 97.9 (1.1) | 86.7 (0.5) | **97.5 (1.2)** |
| Auto – encoder | 91.6 (8.2) | 81.7 (12.4) | 84.4 (14.0) | 95.4 (3.5) | 84.5 (8.9) | 92.1 (8.0) | 93.6 (4.0) | 79.4 (10.9) | 94.4 (4.1) | 94.4 (4.0) | 80.0 (12.9) | 96.2 (2.4) |
| SOM | 90.7 (4.8) | 84.3 (4.0) | 82.1 (18.1) | 93.9 (2.4) | 83.6 (5.1) | 91.6 (7.6) | 93.1 (2.7) | 79.6 (5.7) | 94.9 (2.8) | 94.3 (2.2) | 82.4 (4.3) | 96.5 (1.8) |
| K-means | 91.9 (6.9) | 78.7 (12.8) | 81.8 (14.7) | 97.1 (2.7) | 86.3 (6.4) | 93.8 (5.8) | 96.0 (2.6) | 84.3 (4.1) | 95.6 (3.4) | 96.7 (1.8) | 86.4 (0.9) | **96.9 (1.9)** |

1. The Fourth Case of Infection (Flu)

**Table 8**: Average and standard deviation of AUC, specificity, F1-score for smoothed version of the data with 48 hours moving average filter and different sample size.

| **Fraction = 0.01** | | | | | | | | | | | | |
| --- | --- | --- | --- | --- | --- | --- | --- | --- | --- | --- | --- | --- |
| Models | **Boundary and Domain-Based Method** | | | | | | | | | | | |
|  | 1 Month | | | 2 Months | | | 3 Months | | | 4 Months | | |
|  | **AUC** | **Specificity** | **F1** | **AUC** | **Specificity** | **F1** | **AUC** | **Specificity** | **F1** | **AUC** | **Specificity** | **F1** |
| SVDD | 98.8 (1.3) | 93.1 (2.4) | 91.0 (8.0) | 95.7 (2.4) | 83.7 (5.7) | 86.9 (17.4) | 96.9 (2.0) | 83.5 (3.3) | 94.6 (4.5) | 97.4 (1.1) | 86.3 (5.7) | 92.3 (11.4) |
| incsvdd | 98.4 (1.6) | 92.0 (2.7) | 90.5 (7.6) | 95.5 (2.4) | 82.3 (3.0) | 91.9 (6.3) | 96.6 (2.2) | 83.2 (2.3) | 94.7 (4.0) | 97.3 (1.0) | 83.3 (2.6) | **96.1 (2.9)** |
| $\boldsymbol{\upsilon}$**-**$\boldsymbol{SVM}$ | 99.6 (0.6) | 93.4 (0.0) | **96.0 (1.4)** | 99.1 (1.5) | 86.7 (0.3) | **94.1 (2.0)** | 99.2 (1.2) | 85.7 (0.1) | **94.2 (2.3)** | 99.4 (0.8) | 87.5 (0.5) | 94.9 (2.8) |
| Nearest Neighbour | 98.7 (3.3) | 100 (0.0) | 88.0 (15.5) | 92.8 (4.1) | 84.9 (5.2) | 88.8 (6.9) | 93.8 (1.9) | 80.5 (3.6) | 92.3 (3.6) | 94.3 (1.7) | 80.8 (3.7) | 94.2 (2.5) |
| MST | 99.6 (0.8) | 100 (0.0) | 70.8 (18.2) | 97.8 (0.8) | 97.0 (1.0) | 86.3 (11.1) | 98.1 (0.8) | 96.8 (1.2) | 90.5 (7.3) | 98.4 (0.5) | 97.0 (1.0) | 92.9 (5.1) |
|  | **Density-Based Method** | | | | | | | | | | | |
| Gaussian | 99.5 (1.0) | 96.8 (1.5) | 91.0 (13.7) | 99.5 (0.8) | 89.9 (2.6) | 95.0 (5.1) | 99.7 (0.7) | 93.2 (1.6) | **97.2 (4.0)** | 99.8 (0.4) | 94.4 (1.4) | **97.9 (2.6)** |
| MoG | 99.5 (1.1) | 99.9 (0.6) | 89.7 (16.5) | 99.3 (1.1) | 89.3 (3.9) | 94.0 (6.7) | 99.5 (0.8) | 92.8 (2.8) | 96.4 (5.0) | 99.7 (0.5) | 94.7 (2.5) | **97.8 (3.2)** |
| MCD Gaussian | 99.5 (0.9) | 96.3 (1.9) | **91.7 (11.2)** | 99.7 (0.8) | 89.8 (2.3) | **95.2 (4.3)** | 99.7 (0.6) | 94.1 (1.4) | **97.5 (3.7)** | 99.8 (0.4) | 95.6 (1.4) | **98.1 (2.6)** |
| Parzen | 99.6 (0.8) | 100 (0.0) | 58.8 (14.8) | 98.6 (0.9) | 100 (0.0) | 78.8 (11.4) | 98.9 (0.9) | 100 (0.0) | 85.9 (10.0) | 99.3 (0.6) | 100.0 (0.0) | 89.5 (7.0) |
| Naive Parzen | 97.6 (1.9) | 93.3 (2.4) | 84.1 (13.1) | 98.9 (1.9) | 90.5 (1.4) | 93.3 (8.0) | 98.9 (1.4) | 91.3 (1.3) | 95.2 (5.1) | 99.1 (1.0) | 92.2 (2.1) | 96.6 (3.4) |
| k-NN | 99.3 (1.5) | 100 (0.0) | 84.7 (18.3) | 99.8 (0.6) | 88.9 (3.8) | 94.0 (6.8) | 99.7 (0.5) | 95.9 (1.4) | 96.8 (5.4) | 99.8 (0.4) | 99.1 (0.4) | **98.0 (3.4)** |
| LOF | 99.1 (1.4) | 100 (0.0) | 85.7 (19.6) | 96.8 (1.8) | 83.5 (6.1) | 90.1 (7.1) | 98.3 (1.6) | 84.9 (5.2) | 93.5 (4.8) | 98.3 (1.3) | 85.4 (5.0) | 94.2 (3.9) |
|  | **Reconstruction-Based Method** | | | | | | | | | | | |
| PCA | 99.3 (1.1) | 96.2 (4.5) | **89.8 (15.5)** | 99.7 (0.6) | 90.7 (3.2) | **96.3 (1.5)** | 99.8 (0.4) | 95.9 (1.4) | **98.3 (1.6)** | 99.9 (0.3) | 96.3 (1.5) | **98.6 (1.4)** |
| Auto – encoder | 98.6 (3.6) | 96.6 (7.0) | 88.6 (16.3) | 96.6 (4.1) | 83 (14.1) | 92.4 (6.1) | 96.8 (4.6) | 84.3 (15.5) | 95.1 (4.4) | 97.5 (3.2) | 86.1 (11.3) | 96.6 (2.8) |
| SOM | 99.1 (1.9) | 100 (0.2) | 84.9 (18.9) | 93.5 (2.2) | 84.1 (5.4) | 91.4 (5.9) | 93.9 (2.4) | 84.6 (5.3) | 93.2 (4.4) | 95.2 (3.4) | 88.3 (5.9) | 96.2 (3.1) |
| K-means | 99.3 (1.4) | 99.4 (1.7) | 88.4 (18.2) | 98.7 (1.9) | 85.6 (5.2) | **93.2 (4.6)** | 99.3 (1.1) | 93.7 (3.8) | 96.5 (4.3) | 99.8 (0.4) | 96.1 (2.4) | - 1. **(2.9)** |
